# Supplementary material for: Unraveling the complexities of diet induced obesity and glucolipid dysfunction in metabolic syndrome
Source: Diabetol Metab Syndr. 2025 Jul 22;17:292. doi: 10.1186/s13098-025-01837-y (PMC12281772; doi:10.1186/s13098-025-01837-y)
Supplement: Supplementary file 1 — Additional file 1. [file 13098_2025_1837_MOESM1_ESM.pdf]

MS REVIEW PAPER Revision 2

by Babi Dutta MAHE

Submission date: 21-Jun-2025 02:24AM (UTC+0800)
Submission ID: 2702996044
File name: MS_REVIEW_PAPER_REVISION_2.pdf (898.2K)
Word count: 33597
Character count: 209972

Unraveling the complexities of diet induced obesity and glucolipid dysfunction in metabolic syndrome

Babi Dutta[1], Aparna Tripathy[2] , Archana PR[3], Shobha U Kamath[4*]

Abstract

The consumption of a high-fat high-calorie diet with or without fructose (western or cafeteria diet) increases body mass due to calorie excess, inducing glucolipid metabolism dysfunctions culminating in development of unhealthy obesity and metabolic syndrome. Understanding the sequelae of events that translates caloric excess to the development of metabolic syndrome symptoms interlinking metabolic interrelationship between organs is paramount in the development of new treatment strategies. This review aims to create a compendium of evidence from mammalian studies (rodents, humans) to elucidate the metabolic changes induced by overnutrition. This review explores gut microbiome alterations, gut barrier dysfunctions, and immune dysregulation induced by a high-fat diet that changes gut tryptophan and biliary metabolism, which, with concomitant elevations in free fatty acids and ceramides, promote insulin insensitivity. Immunometabolic alteration induce adipose tissue dysfunction, which alters the secretion of adipokines and lipid metabolites that contribute to dyslipidemia, hepatosteatosis,

cardiovascular dysfunction, and endocrine disruption. This review provides insights into the mechanism underlying unhealthy adipose expansion, shedding light on some of the exosome-mediated epigenomic alterations affecting obesity or metabolic syndrome pathogenesis, which may help in the future design of microRNA biomarkers. The review also highlights areas where more supportive evidence may be needed to elucidate metabolic syndrome pathogenesis.

Key Words: Epigenomics, High-fat high-calorie diet, Insulin insensitivity, Lipid alterations, Metabolic syndrome, Adipose tissue dysfunction

Abbreviations: High fat diet (HFD), high-calorie high fat diet (HCHF), insulin receptor substrate (IRS), mammalian target of rapamycin (mTOR), AMP-activated protein kinase (AMPK)

Introduction

Eating to live emphasizes the most basic definition of food providing nutrients for survival and maintenance. Food or diet has few essential constituents such as carbohydrates, lipids, and proteins ingested each day in their recommended proportions constituting daily energy intake are reduced to their most basic monomeric units throughout the digestive process. The digestive end products are absorbed, transported and utilized through two distinct channels: the oxidative route generating energy (ATP) and reconversion to complex forms comprising anabolism, stored as glycogen or lipids, or converted into tissue proteins. Energy expenditure involves maintaining a resting metabolic rate, specific dynamic action, and physical activity. Energy intake and expenditure are nearly in balance in healthy persons, but if energy acquisition (overnutrition) exceeds energy expenditure, this results in an imbalance, with acute or long-term imbalances manifesting as harmful effects on health. Different open- and closed-feedback loop systems govern the body's systems, with many systems having thresholds values depending on

physiological variables. Homeostatic systems like body temperature have a set-point where corrective actions are initiated with any significant variations from set-point. Body weight regulation is a dynamic system as opposed to others and is sensitive to a variety of external and internal stimuli. Body weight set-point is also variable, with corrective action being less severe in overweight or more adiposity-prone individuals [1] and more severe if it falls below the set point, depending on the circumstances. One of the major complications of overnutrition is obesity, where excess energy is stored as fats, initiating a sequence of events ultimately leading to obesity and associated metabolic pathologies like diabetes, hypertension (increased systolic blood pressure (SBP) and diastolic blood pressure (DBP), cardiovascular issues, and metabolic syndrome. The problem of obesity or overweight is a potent healthcare challenge with increasing obesity incidence affecting both adults and children worldwide. According to the WHO, obesity affects 650 million adults, 340 million adolescents and 39 million children, resulting in an excess of 1 billion obese individuals which by 2025 will affect 167 million people [2]. In developing countries such as India, there is a worsening of abdominal obesity, with 40% of women and 12% of men exhibiting abdominal obesity, 23% of women, and 22.1% of men being overweight or obese, according to body mass index (BMI) [3].

Multiple factors contribute to the increasing prevalence of obesity, such as changes in dietary habits and decreased physical activity, with concomitant changes in socioeconomic factors, such as easy availability of palatable energy-dense foods and energy-saving appliances. Systems biology approaches toward obesity include global per- capita income changes, increased urbanization or modernization, changes in government policies that attract large foreign conglomerates that provide greater food choices, and the expansion of fast-food chains with increasing frequency, offering easy-palatable foods [4]. One of the most damaging consequences of modern-day altered dietary habits is an increase in the consumption of free sugars in the form of fructose or sucrose, which has greater health implications. The primary forms of sugar or fructose consumed come from sweets, soft drinks, juices, high-fructose corn syrup, etc. Studies have indicated an increase in fructose consumption in adults and children, with one study conducted in German children demonstrating fructose consumption three times more than the

recommended dietary allowance [5]. Fructose or sucrose consumption is positively corelated with increasing body weight, increased risk of endocrine-metabolic disturbances, cardiovascular diseases, and even cancers [6]. Western or cafeteria-based diets with a large proportion of their energy contributed by fats (primarily monounsaturated fats) and sugars also contribute to growing problems of overnutrition and obesity.

One of the common metabolic disturbances accompanying obesity is metabolic syndrome (MetS), which according to NCEP ATPIII guidelines, is the presence of any of three out of five criteria which include obesity with a waist circumference > 40 inches in males or > 35 inches in females; fasting glucose levels ≥ 100 mg/dL, dyslipidemia with triglycerides (TAG) ≥ 150 mg/dL; high-density lipoprotein cholesterol (HDL-C) < 40 mg/dL in males or < 50 mg/dL in females, hypertension with SBP > 130mmHg and DBP >85 mmHg and other criteria such as hyperinsulinemia or insulin resistance, and reduced serum adiponectin levels.

Review Aim

The critical concepts highlighted by ongoing research reflect the dynamic nature of our understanding of MetS and evaluating individual components such as insulin resistance and impaired insulin signaling, abdominal obesity, oxidative stress, and defective mitochondrial biogenesis is important. However, a multifaceted approach towards the pathogenesis of MetS is needed. It thus stems the need for a review that compiles the current multiple pieces of evidence from mammalian studies, mainly from rodents and humans, interlaced with what is already known to present a probabilistic sequence of events in the pathophysiology of metabolic syndrome.

In line with this aim, a literature search was performed in the PubMed, Google Scholar and Scopus databases, with key words such-as high-fat diet, fructose, metabolic syndrome, obesity, lipid metabolism alterations, and immunometabolism shortlisting articles on the basis of relevance, abstracts, and full texts. Emphasis on the literature published in last six years was given to infusing new evidence into the pathophysiological framework of metabolic syndrome.

1. The act of consumption and metabolic regulation

1.1 Process of digestion: The dietary components undergo a rigorous breakdown process, breaking down complex dietary components into their simplest forms. Dietary carbohydrates are broken down by salivary, pancreatic, and intestinal brush border enzymes, producing monosaccharide units such as glucose, galactose, and fructose. Intestinal bacteria metabolize undigestible carbohydrates to produce beneficial short-chain fatty acids (SCFAs) (namely acetate, propionate, and butyrate) with minimal contribution from amino acid metabolism [7], which can enter epithelial cells using transporters. Dietary proteins are broken down into constituent amino acids by digestive proteolytic enzymes which are then absorbed. Gut microbial can also degrade some dietary amino acids such as tryptophan, which can be metabolized into indole and their derivative such as indole-acetic acid, acetaldehyde, indole-propionic acid, skatoles, or kynurenine metabolites or serotonin or tryptamine [8]. The dietary lipids include triglycerides (TAGs), cholesterol, cholesterol esters, and free fatty acids (FFA), which are large lipid droplets, are emulsified by bile acids/salts. After emulsification, the lipids are hydrolyzed by pancreatic enzymes into 2-monoacyl glycerol (2-MAG), FFAs, and cholesterol which are then absorbed via transporters such as CD36 and fatty acid transport protein's (FATPs) and Niemann Pick C1-Like-1 (NPC1L1). The MAG transporter lipid transporter CD36 is important in diet-induced metabolic syndrome since it can regulate fat intake via oleoyl ethanolamide (OEA) production, and glucagon like peptide-1 (GLP-1) secretion (also influenced by SCFAs) [7, 9, 10].

1.2 Absorptive process and its regulation: The termination of the digestive process marks the beginning of the absorptive process, that allows the digestive end-products to reach tissue for utilization and storage. The intestinal epithelial cells (enterocytes) possess transporters that helps in the entry of digestive end-products into epithelial cells.

Monosaccharides such as glucose, fructose and others are absorbed via a sodium-dependent glucose transporter 1 (SGLT-1) system or via the glucose transporter-5 (GLUT-5) in the case of fructose and then transported into blood via GLUT-2. The absorbed carbohydrate travel via blood and are ultimately taken up by all tissue using different glucose transporters which are both insulin-

independent and insulin-dependent. Short–chain fatty acids (SCFAs), are absorbed by nonionic diffusion, SCFA/HCO_3^- exchangers, active transport, hydrogen-coupled monocarboxylate transporter 1 (MCT1) or sodium-coupled monocarboxylate transporter 1 (SMCT1) [11]. Gut luminal SCFA also can induce release of gut-derived chemical signals like peptide YY (PYY) and GLP-1 which promote satiety and regulate food intake (discussed in greater detail under incretins section). SCFA like propionate and butyrate promote intestinal gluconeogenesis and fatty acid oxidation (FAO) providing energy, exerting antiobesogenic effects [8, 12-15]. SCFAs such as butyrate and propionate also help to support the gut epithelium cells by inhibiting apoptosis and inter-cellular connections by stabilizing tight junctions [16-18] (Refer figure 1).

Amino acids also enter the intestinal epithelial cells via sodium-dependent co-transport mechanism after which they are transferred into blood using facilitated transporters with different amino acids using different transporters. Amino acid derived products such as indoles can enter the blood stream via diffusion through lipid membrane of intestinal cells. The indoles and their derivative also interact with aryl hydrocarbon receptors (AhR) present in epithelial and immune cells to maintain gut epithelium [19-22].

Lipid digestion products, upon entry into enterocytes, are shuttled into different pathways with FFA and MAGs intracellularly bound to fatty acid-binding proteins (FABPs) and retinol-binding protein 2 (RBP2) with minor quantities of MAGs hydrolyzed by monoglyceride lipase (MGL) to facilitate endocannabinoid production affecting food consumption and gut junctional integrity [23]. The majority of absorbed MAGs are re-esterified to TAG by acyl transferases (monoacylglycerol and diacylglycerol acyltransferases MGAT1, 2 & DGAT1, 2), with DGAT subtypes preferentially allocating TAGs either for incorporation into chylomicrons (CMs) or cytoplasmic lipid droplets (CLDs) [24]. DGAT1 is involved primarily in TAG allocation for chylomicron synthesis, with DGAT2 playing a secondary or compensatory role [24, 25]. The functionality of DGAT2 becomes significant in cases of a high-fat diet since a high-fat diet can stimulate the RNA-binding protein HuR (ELAVL1)y [26]. HuR stabilizes DGAT 2, which allows for greater absorption of dietary lipids and

packaging into TAGs for increased absorption, which was ablated by HuR knockout models or DGAT1/DGAT2 inhibitors [25, 26]. TAGs destined for CMs are transported to the endoplasmic reticulum (ER) and then loaded into apolipoprotein B48 (Apo B48) by microsomal transfer protein (MTTP), followed by Golgi transport and secretion into the lymph [24], which are regulated by lymphatic endothelial-cellular junctions. The lipid transporter CD36 acts as a lipid sensor, promoting CM formation via induction of ApoB-48 and MTTP and promoting vesicle budding for CM synthesis [9]. HFD increases jejunal CD36 expression and thus can regulate CM production [9]. The chylomicron is secreted into the intestinal lacteals which carry them into the systemic circulation, are serve as important control points for absorption of dietary TAG-laden chylomicrons. Lacteal junctions are generally of two types: "button-like" junctions that facilitate CM secretion, and "zipper-like" junctions that inhibit CM entry and prevent obesity [24]. The gut microbiome can also regulate intestinal lacteal junctional integrity and transition via vascular endothelial growth factor-C (VEGF-C) expression; however, it is still unclear which microbial species regulate this process [27]. The lacteal released chylomicron enter systemic circulation from where they deliver TAGs to extra-hepatic tissues (adipose tissues) and the reminant lipid absorptive products are finally delivered to liver for utilization.

All of the absorbed TAGs are not transported via CM, some of the TAGs are diverted toward lipid droplet (CLD) synthesis, which requires components such as phospholipids and coating proteins like perilipins (Plin) (major murine intestinal perilipins are 2 & 3). Lipid droplet synthesis is protective, delaying excess TAG absorption during high-fat diet consumption and protecting cells from FFA lipotoxicity [28]. CLD-resident TAGs are hydrolyzed by lipases namely adipose triglyceride lipase (ATGL), hormone-sensitive lipase (HSL), monoacylglycerol lipase (MGL) or carboxylesterase 2c (CES2c), which provide fatty acids for fatty acid oxidation (FAO) via oxygen, creating luminal hypoxia [29, 30]. CES2c-mediated lipolysis can provide DAG for chylomicron (CM) synthesis with human obesity-linked IBD associated with a reduction in CES2 [29, 30].

The absorptive process is thus one of the earliest steps which if increases, can cause excessive absorption of dietary components.

1.3 High-fat diet and altered absorption: High-fat diet consumption can significantly alter the lipid absorption rates. Chronic high-fat intake in mice increases jejunal fatty acid uptake by upregulating key genes involved in fat absorption, such as FATPs (FATP-4), CD36, FABPs (FABP-1), MTTP, Apo A-IV and Cpt1a [31, 32]. In MetS mice models, a HFD increases lipid delivery, increasing L-FABP and MTTP mRNA levels in the terminal ileum, and increasing ileal fat absorption. A HFD in mice reduces intestinal IL-25 production, which increases the expression of SGLT-1 and GLUT-2 transporters, facilitating glucose uptake [32]. HFD consumption via IL-25 reduction also increases the expression of genes involved in cholesterol uptake (*NPC1L1*) and chylomicron production (*Mogat2*, *Apobec1*, *Dgat1*, *and Acat2*) in the jejunum [32]. HFD-induced increased lipid delivery reorganizes the CLD proteome, increasing murine Plin-2 expression [33] but not Plin-3 expression and reducing choline–phosphate cytidylyltransferase A (Pcyt1a), increasing CLD size. Large CLDs store more TAG, and low TAG hydrolysis rates cause fat droplet enlargement [11, 28, 33], indicating a greater epithelial lipid storage capacity which can then be repackaged into chylomicrons, initiating greater lipid delivery to tissues.A single day of HFD intake increases the levels of cellular stress proteins and unfolded protein responses in the Paneth cell endoplasmic reticulum [33] which might affect Paneth cell functionality, and alter microbial populations, which might reduce populations of those bacteria's such as *Lactobacillus* (regulates chylomicron release to lymphatics), thus prompting greater lipid transport (Refer figure 1).

High-fat diet feeding upregulates lymphatics-related genes such as Lyve-1, VEGF receptor-3, and Prox-1 in the jejunum along with VEGF-C expression, inducing button-like junctions for greater chylomicron clearance. However, high-fat diet consumption coincides with the lymphatic junctional transition from the button to the zipper. This transition indicates an inherent protective mechanism that occurs via the action of calcitonin gene-related peptide and receptor activity-modifying protein 1 (RAMP1) action [34]. RAMP1 knockout result in significant lymphatic drainage with button-like

junctions [34]. A recent study indicated that chylomicrons in newborn mice can modulate lacteal junctions via Rho-associated kinase (ROCK)-dependent inhibition of VEGF-A and downregulation of VEGF receptor-2 (VEGFR-2), both of which promote zipper-like junctional integrity [35]. Evidence of such a change in high-fat diet consumption is still lacking, given that levels of intestinal chylomicrons are elevated due to high-fat diet consumption.

Thus, HFD-induced oversized lipid droplet accumulation and elevated jejunal and ileal lipid absorption increase overall lipid delivery [33], thus highlighting the role of the small intestine in obesity and MetS etiology. In cases of HCHF diet, a greater lipid and glucose/fructose absorption is achieved that enter into all tissue, especially in adipose tissue and liver. In cases of excessive glucose consumption, it is metabolized in both adipose tissue and liver, whereas in cases of excessive fructose consumption, liver is the major metabolic organ handling fructose metabolism.

Figure 1: Dietary nutrient digestion, absorption and gut barrier integrity

Figure 1A: Digestive process of carbohydrate, lipids and amino acid tryptophan- Digestible carbohydrates are broken down to simple monosaccharides, while undigestible carbohydrates are degraded to SCFAs, which in turn regulate the microbial metabolism of tryptophan-producing indoles and inhibiting kynurenine production. Lipid digestive products are 2-MAG, FFAs and cholesterol.

Figure 1B: The absorption of lipid digestive products such as 2-MAG, and FFA upon entry into enterocytes are reesterified to TAG and packaged into chylomicrons, which involves enzymes like DGAT 1 and 2. Some TAGs are packaged into cytoplasmic lipid droplets to regulate chylomicron flux. FAO metabolism stimulated by SCFAs promotes gut luminal anaerobicity.

Figure 1C. Absorption of monosaccharides such as glucose and fructose- with glucose, 2-MAG and FFA mediated release of gut endocrine signals like GLP-1 and GIP which promotes satiety and insulin secretion from pancreatic β-cells. SCFAs contribute to intestinal epithelial cell energy metabolism and gluconeogenesis, which exert antiobesogenic effects.

Figure1D: Maintenance of gut homeostasis by via SCFA-mediated AMPK activation which promotes epithelial survival and tight junctions' maintenance by upregulating tight junctional proteins. Indole

and its derivative also regulate intestinal barrier function, tight junction proteins, and promote epithelial regeneration via NOTCH signaling. Homeostatic gut microbial species *Akkermansia muciniphila* along with MGL promote endocannabinoid release regulating hedonic food consumption, and gut permeability. *Bifidobacterium bifidum* present in the GI lumen upregulates the expression of tight junction.

2-MAG: 2-monoacyl glycerol, TAG: triacyl glycerol, FFA: free fatty acid, DGAT: diacylglycerol acyltransferase, MGL: monoacylglycerol lipase, CES2c: carboxylesterase 2c, SCFA: short-chain fatty acids, CLD: cytoplasmic lipid droplet, AMPK: AMP-activated protein kinase, GLP-1: glucagon like peptide-1, GIP: gastric inhibitory polypeptide [Figure 1 legend ends]

1.4 Incretins and their role in eating and metabolism: Incretins are gut-derived nutrient-stimulated chemical messengers that regulate critical consummatory behaviors and systemic metabolism. Glucagon-like-peptide-1 (GLP-1) is one of the major incretin hormones secreted by L-cells of the distal small intestine, colon, and rectum [36-38]. GLP-1 released in the gut has a short lifespan due to its rapid degradation into smaller GLP-1 derivatives with reduced potency by dipeptidyl peptidase-4 (DPP-4) enzyme followed by renal excretion [37]. The major intestinal stimulators of GLP-1 secretion are carbohydrates (glucose and fructose), glutamate, and lipids (TAGs, 2-MAGs, SCFA), with lipid species producing a more pronounced effect than carbohydrates [36]. GLP-1 receptors are also found in other human tissues, such as pancreatic alpha-cells and acinar cells, cardiac muscles, and white adipocytes. In contrast, in non-human primates it is found in brain (hippocampus, amygdala, substantia nigra and cerebellum) [36]. GLP-1 slows gastric emptying, reduces appetite, and food intake, and improves glycemic control. GLP-1 also acts via neural circuits in rodents to regulate appetite and hedonic consumption behaviors [39, 40]. Prominent systemic effects of GLP-1 include reduced calorie intake, glucagon secretion, CM production, hepatic glucose production, increased glucose uptake, glycogen synthesis, and reduction of intrahepatic fat accumulation, inflammation and steatosis [36, 38, 39]. GLP-1 actions on brown adipocytes promote thermogenesis

and influence energy expenditure [39]. Another hypothesis suggests that GLP-1 appetite-suppressive effects may be mediated via vagus nerves, since vagus denervation abolishes appetite-suppressing effects [41].

Another important incretin is glucose-dependent insulinotropic polypeptide (GIP) released from K-cells of the duodenum and upper jejunum. The intraluminal presence of carbohydrates (glucose and fructose), saturated fatty acids promote GIP release with fatty acids producing a greater GIP release than carbohydrates [36]. GIP receptors are found in other tissue in humans and rodents such as pancreatic alpha-cells and cardiac muscles in humans while in rodents GIP receptors are in brown adipocytes, and brain (hippocampus, amygdala, substantia nigra and cerebellum) [36]. GIP promotes de novo lipogenesis in white adipocytes and induces fat/lipid storage in adipocytes with GIP knockouts in adipocytes reducing HDF-induced obesity [36, 39]. GIP actions on white adipocytes also include stimulation of lipolysis and reduction of inflammation [39]. GIP on brown adipocytes promotes glucose and amino acid catabolism [39]. GLP-1 and GIP are stimulated by luminal glucose concentrations, mainly via SGLT-1 mediated sodium and glucose uptake that promotes fusion of GLP-1 containing vesicles in enteroendocrine cells [41]. Luminal long-chain fatty acids that enter enterocytes via CD36 are activated by long-chain acyl CoA synthetase (ACSL3) which then through PKC mediated pathways, promote GLP-1 or CCK signaling [42] Long-chain fatty acids obtained via basolateral lipolysis of chylomicrons can also promotes GLP-1 and GIP release via GP40, GP119, and GP120 receptors [41]. Both the hormones GLP-1 and GIP act on pancreatic beta-cells via cognate receptors to promote the release of insulin [36]. The actions of GLP-1 and GIP on glucagon secretion are different, wherein GLP-1 reduces glucagon secretion, but GIP promotes glucagon secretion in cases of low plasma glucose concentration [36]. The number of incretins released is dependent on the amount of oral-glucose load and plays a maintaining normoglycemic limits [36]. GIP also has systemic effects, wherein it acts on adipocytes, promotes glucose uptake, TAG uptake and storage, reduces hepatic glucose production, and increases glucose uptake and glycogenesis [36].

Another gut-derived hormone, cholecystokinin (CCK), is released from EEC cells of small intestine [39]. Major dietary factors that promote CCK release include lipids (fatty acids via G-protein-coupled receptors–40 (GPR40) receptors or cluster of differentiation–36 (CD36) dependent mechanisms) or amino acids [41]. CCK via the vagal neuronal axis reduces food intake, suppresses hepatic glucose production [41]. HFD ingestion even for short durations impairs the CCK signaling via alterations in vagal afferent neurons [41].

The presence of homeostatic gut microbes modulates the number of incretin-secreting cells and their nutrient-responsive behaviors since germ-free rodent models show differences in GLP-1 secreting cell transcriptome signatures and are GLP-1 resistant [39]. The gut microbiome directly modulates EEC cell-intrinsic pathways by regulating GPR receptor expression [42] or indirectly through the modulation of enteric neurons (via lipopolysaccharide (LPS)-Toll-like receptor 4 (TLR4) or 5-HT(5-hydroxy tryptophan)) [39]. One of the predominant microbial species, Lactobacillus (L. gasseri), present in the homeostatic gut, helps to maintain SGLT-1 expression and promotes nutrient sensing by GLP-1 and CCK-secreting cells [42]. Normal bile acid profile also upregulates ACSL3 and thus promotes GLP-1 and CCK release improving metabolic homeostasis [42].

Another gut-derived hormone Peptide YY (PYY) is released from L-cells of the gut along with GLP-1, which is released in two-phases-the first one is neural phase dependent on CNS stimulations, whereas the second is arrival of food in GI tract which stimulates PYY release. PYY belongs to neuropeptide Y (NPY) category peptides [43, 44]. The release of PYY is also stimulated by SCFAs (namely propionate and butyrate) via histone deacetylase action and/or GPR32 [45]. PYY interacts with NPY receptors in CNS producing appetite-suppressing effects and plays a role in maintenance of pancreatic beta-cells, thus affecting normoglycemia in post-prandial stages [43, 44]. The importance of PYY is homeostatic maintenance can be gauged from studies that show that HFD induced pancreatic beta-cell apoptotic death which is reduced by PYY treatment [46].

HFD-induced gut microbial population changes alter the LPS concentrations, which impairs TLR4-vagal pathways and alters the incretin-appetite suppression pathways [41]. Another hypothesis

suggests that HFD consumption upregulates potassium channels in vagal afferents, impairing vagal impulse propagation via hyperpolarization [41]. HFD consumption alters the microbial profile, which may independently alter the GPR presentation on EEC cells thus reducing their lipid sensing capabilities and GLP-1 or CCK release [42]. HFD-induced LPS production downregulates SGLT-1 or ACSL3 which can impair GLP-1 or CCK release. HFD-induced reduction in the population of L. gaasseri also impacts the SGLT-1 expression in EECs. HFD-induced elevation in Bacteroides fragilis also dysregulates the bile acid profile, reducing farnesoid-X-receptor (FXR) signaling, which may also reduce GLP-1 or CCK secretion [42]. Thus, the response of GLP-1 is blunted or reduced in obesity caused by dietary modulations [47], and GLP-1 agonists such as liraglutide and semaglutide (Ozempic) have been shown to improve weight loss, induce fat mass loss, and regulate lean muscle mass in obese human subjects [47]. HFD consumption in humans and rodents increases GIP signaling, mainly in brain GIP signaling, which promotes body weight gain, adiposity due to GIP-induced central leptin resistance [41]. HFD ingestion, even for short durations, impairs the CCK signaling via alterations in vagal afferent neurons [41]. Consumption of HFD impairs the release or functionality of PYY as observed in rodents where HFD alters the population of ileal PYY positive cells and reduces ileal concentrations of GLP-1 and PYY [46]. Studies conducted in human obese subjects have yielded no significant difference in PYY levels both at protein and mRNA levels when compared to lean subjects [48].

The successful usage of GLP-1 agonists in the treatment of obesity and type 2 diabetes has sparked significant interest in translating the effects of these drugs in obesity and appetite suppression. Recently, multiple drugs like vildagliptin (DPP-4 inhibitors) have also shown promising effects in reducing central adiposity and hyperglycemia [49-51]. The role of GIP is still unclear since GIP favors lipid accumulation in white adipocytes. However, recently drugs like tirzepatide, which stimulate GIP receptors, have shown greater effectiveness in weight reduction as compared to solitary GLP-1 receptor agonists [50, 51], indicating that newer research should focus more on the functionality of GIP and effects achieved due to co-stimulation of GLP-1 and GIP receptors. Recent

studies conducted in obese rodents using non-specific PYY receptor agonist and semaglutide have shown significant weight loss [52], indicating that more clinical trials are necessary to elucidate such combinatorial drug therapy in mitigating obesity. The glucose and fat-sensing capabilities of gut in obese people are significantly impaired. However, protein or amino-acid sensing abilities are retained, due to unknown reasons, which can be utilized to design drugs to promote a greater nutrient sensitivity and incretin release in obese subjects via amino-acid sensing properties.

Thus, the consumption of food, digestion and absorption of dietary components are both influenced by combined effects of host enzymes, digestive by-products and gut-derived signalling molecules (influenced by gut-resident microbes) which synergistically act to influence host absorptive functions. HFD or HCHF with or without fructose increases the rates of intestinal lipid absorption, impacts satiety pathways and thus allows greater metabolic flux into tissues which marks the beginning of nutrient excess conditions. The gut microbial population not only regulates host consummatory pathways and also metabolizes some dietary components to generate bioactive compounds. These bioactive metabolites exert systemic and local effects and support both gut and immune homeostasis, so an understanding of how a diet of HFD or HCHF induces changes in gut microbial populations would be paramount in understanding many recent treatment strategies using probiotics to attenuate HFD-or HCHF-induced metabolic maladies.

2. Gut microbes and their metabolites- A bidirectional dynamic relationship

The gut environment has a rich microbiome predominantly with an anaerobic phenotype due to the anaerobicity of the existing gut lumen. Gut epithelial cells employ mitochondrial fatty acid oxidation with oxidative phosphorylation (OXPHOS) and utilize large quantities of oxygen. The colonic cells also metabolize glucose via anaerobic glycolysis with lactate production (Warburg metabolism) for energy requirements [55]. High oxygen utilization reduces partial oxygen pressure in the gut lumen ("epithelial hypoxia") which propagates the expansion of obligate anaerobic gut microbes [53]. The predominant gut microbiome in the normal human gut are *Bacteroidetes* and *Firmicutes*, accounting for 90% of all microbes, with minor phyla such as *Actinobacteria, Proteobacteria, Fusobacteria,* and

Verrucomicrobia accounting for the remaining 10%. Among Firmicutes, the most abundant genera are *Clostridium*, followed by *Bacillus, Lactobacillus, Enterococcus,* and *Ruminococcus*. Among *Bacteroidetes*, the most abundant genera are *Bacteroides* and *Prevotella,* followed by *Parabacteroides and Alistipes*, and among *Actinobacteria*, the primary genus is *Bifidobacterium* [54]. Among these phyla, specific bacteria are present in different proportions rather than all members, indicating that variation in species populations is possible within one phylum. Certain species present in the healthy homeostatic gut of rodents and humans include members of Firmicutes family such as *Erysipelotricheles, Turicibacter, Roseburia* [55, 56], *Ruminococcaceae (UCG-014*, *UCG-010, NK4A214*, *unclassified), Faecalibacterium (Faecalibacterium prausnitzii)*, *Blautia*, *Gemella*, *Anaerofustis*, *Akkermansia muciniphila (Verrucomicrobia), Romboutsia*, *Coriobacteriaceae_uncultured*, *Catabacter (Christensenellaceae family)*, *Mollicutes RF9*, *Acetanaerobacterium, Clostridiales_vadinBB60_group* [56, 57] and *Eubacterium cylindroides* [56, 58]. The *Bacteroidetes* in the normal gut include *Prevotellaceae*, *Bacteroides (Bacteroides thetaiotaomicron), Prevotella*, and low concentrations of *Intestinibacter (Firmicutes)* [57]. The presence of specific bacteria is not random but is a carefully coordinated player, where each microbial species plays a role in the maintenance of gut homeostasis. Presence of lean gut homeostatic bacteria such as *Akkermansia muciniphila* influences the production of endocannabinoids regulating hedonic food consumption, glucose-energy metabolism, and maintains normalizing the tight junction proteins [59, 60]. *Bifidobacterium bifidum* present in the lean GI lumen upregulates oxidant- scavenging enzymes and anti-inflammatory cytokines such as IL-6 and -10; downregulates the expression of inflammatory genes such as tumor-necrosis factor-alpha (TNF-α) and interleukin-1beta (IL-1β); and promotes the expression of tight junction proteins [59, 60]. This indicates that gut microbes can directly regulate the gut barrier homeostasis.

The gut microbes interact with dietary components, metabolizing them and impacting host metabolism. The importance of specific bacterial species in intestinal homeostasis also stems from the fact that bacterial degradation of dietary components results in the generation of multiple bioactive

molecules with far-reaching systemic influences. These normal anaerobic gut microbes metabolize indigestible carbohydrates by saccharolytic degradation to generate SCFA's, such as acetate, propionate, and butyrate, with negligible SCFA contributions from the metabolism of dietary amino acids [61, 62]. The production of different types of SCFAs and their relative proportions are determined by the gut microbial type and play important roles in the maintenance of normal gut immune balance and luminal anaerobicity by impacting the enterocyte metabolic profile.

2.1 Gut metabolite – short-chain fatty acids: In the normal lean gut, bacterial species, namely, *Rikenellaceae (Bacteroidetes), Christensenellaceae*, *Bifidobacterium (Bifidobacterium adolescentis, Bifidobacterium bifidum)*, *Oscillospira (Clostridia-Firmicutes)*, *Akkermansia*, *F. prausnitzii,* and *Roseburia*, among others, help in the production of SCFAs and maintain low luminal LPS concentrations [63]. SCFAs, such as acetate, butyrate and propionate have the potential to promote the production of cytokines from intestinal mucosal immune cells or enteroendocrine cells, which can then modulate the energy and lipid metabolism of gut epithelial cells and interact with vagal sensory nerves to transmit information to the brain affecting the overall energy balance of the body. The gut microbiome of isocaloric diet-fed people include a population of species that are SCFA producers whereas humans or rodents consuming high-fat and/or high-calorie diets show a reduction in these species (Table 1 shows the few selected microbial species that generate SCFAs in homeostatic gut).

Table 1: Different microbial species found in the gut during lean homeostatic conditions and altered profile associated with high-fat diet consumption

SCFAs produced in gut lumen are maximally absorbed with only 5–10% excreted via feces [97, 98]. Luminal SCFAs can enter colonic cells via transporters, providing energy for 60–70% of their energy requirements, and the unmetabolized SCFAs are then transported into the portal circulation, where they enter hepatocytes. In liver, some of SCFAs are utilized and remaining SCFAs escapes into general circulation at concentrations of 30% (acetate), 9% (propionate) and 2% (butyrate) contributing to energy production and accommodating 5–15% of total body energy requirements [25],

after which they then enter the brain via the blood–brain barrier on the basis of their order of permeability: butyrate > propionate > acetate.

Colonic epithelial cells utilize SCFAs by interacting via G-protein-coupled receptors 41 (GPR 41) and 43 (GPR 43) [99] (also expressed in adipocytes, skeletal myocytes and hepatocytes, [100, 101]) with butyrate being the most potent activator of GPR43, and in the case of GPR 41 the potency of activation is butyrate ≈ propionate > acetate [102]. Another receptor for SCFAs, GPR109a, is found in intestinal cells, immune cells, the brain, the spleen, and adipocytes activated by butyrate [100]. Butyrate, a prominent SCFAs, is utilized by colonic epithelial cells for energy and can activate Peroxisome proliferator-activated receptor's (PPARs) inducing fat oxidation, which helps maintain gut anaerobicity and regulates the differentiation of epithelial cells. Butyrate also stimulates gut hormones reducing appetite [43, 47], activates energy expenditure by stimulating thermogenesis in brown adipocyte [102]. Gut-derived SCFAs promote insulin sensitivity, reduce adiposity and body weight [102-106] and downregulate PPARα, increasing oxidative metabolism in the liver and adipose tissue and thus lowering fat accumulation, and hepatic steatosis and increasing insulin sensitivity [88, 102]. In the hepatocytes, acetate (in small quantities) is diverted toward cholesterol, and fatty acid synthesis; however, major quantities of acetate via systemic circulation enter the brain, regulates the neural circuitry of food intake (appetite) and energy expenditure [25, 102, 107] and protect against obesity [108]. SCFA's like propionate interact with different areas of the brain acts via Free Fatty Acid Receptor 3 (FFAR3) regulating body weight via sympathetic nervous system activity [88, 102] and modulating serotonin production pathways. SCFAs in the brain can also alter the levels of specific neuropeptides and neurotransmitters, for example, in mice acetate affects glutamate/glutamine, and GABA in the hypothalamus and increases anorexigenic actions [109].

SCFAs can also act at epigenomic levels by altering the transcriptional accessibility achieved via histone deacetylase (HDAC) action, where butyrate at high doses promotes histone acetylation and at low doses alters gene expression in the prefrontal cortex. SCFAs also increase insulin levels (via GSIS) independent of blood glucose concentrations, and increase muscular and systemic insulin

sensitivity, and obesity-related reductions in SCFAs promote peripheral insulin resistance and consequently hyperinsulinemia [102]. Some studies have also indicated that SCFA-GPCR interaction is downregulated in the presence of high-carbohydrate meals and that obesity impacts lipogenesis [110], but however one of the accepted hypotheses is dietary alterations may alter microbial populations which can alter the production of SCFAs.

2.2 Gut tryptophan metabolites: Gut bacteria can metabolize dietary tryptophan via multiple pathways, such as the kynurenine pathway, which produces kynurenine and its metabolites regulated by indoleamine-2,3-dioxygenase (IDO), and tryptophan-2,3-dioxygenase (TDO); the production of indole and its derivatives, such as indole acetic acid, lactic acid, propionic acid, and tryptamine by the gut microbiota [111]. The dietary tryptophan can also be metabolized to melatonin, serotonin and its predecessor 5-hydroxy tryptophan (5-HT) in gut and brain, which influences the process of peristalsis, intestinal secretions and absorption [111, 112]. The synthesis of 5-HT is stimulated by butyrate (activate tryptophan hydroxylase) [112] and secondary bile acid (regulate the synthesis and release of 5-HT) with around 95% of the body's 5-HT is stored in ECC cells. Under normal homeostatic gut conditions, the metabolism of tryptophan is skewed toward the synthesis of indole and its metabolites by microbial species such as *Bacteroides* spp. (*Bacteroides thetaiotaomicron, Bacteroides ovatus*) , *Clostridium (Clostridium limosum, Clostridium bifermentans*), *Bifidobacterium (Bifidobacterium adolescentis, Bifidobacterium bifidum), Burkholderia* spp., *Lactobacillus (L. paracasei, L. reuteri),* (stimulated by a high-fiber diet) [113], which reduce kynurenine and serotonin metabolites (dependent on carbohydrate intake) [114, 115]. The SCFA–butyrate also influences this skewness by impairing IDO via signal transducer and activator of transcription-1 (STAT-1)-dependent and -independent mechanisms (via HDAC) [116]. The indole and its derivatives demonstrate a bidirectional relationship with gut microbial populations wherein the presence of bacterial species such as *Clostridium sporogenes* and *Ruminococcus gnavus* increases the conversion of tryptophan to indole metabolites [113], which induces the expression of *Lactobacillus* which further elevates the conversion of tryptophan to indoles [113]. The indole and its metabolites in the gut luminal

environment interact with aryl hydrocarbon receptors (AhRs) which help to maintain gut barrier integrity and regulate gut immune cells such as regulatory T-cells [113] and B-regulatory cells to modulate HFD–induced obesity [117]. In normal/standard chow-fed rodents, microbial degradation of tryptophan also results in the generation of 5-hydroxy indole acetic acid (5-HIAA) through canonical and noncanonical pathways (mediated by *Burkholderia* spp. *(Proteobacteria))* [118]. This 5-HIAA produced in the gut escapes into the systemic circulation, promoting hepatic insulin sensitivity via the AhR-TSC2-mTORC1 mediated pathway [118].

2.3 Other systemic and local effects of gut microbes: The microbial population of the gut is correlated with their functionality; thus certain species are very important under lean conditions. Most microbial populations exert their effects directly via the SCFAs produced, altering host physiological, and metabolic pathways. Some microbes can also modulate systemic effects, like *Prevotella* and *Oscillospira*, which are negatively correlated with body weight [119]. *Prevotella* species are positively correlated with low waist circumference, fat mass, improved glucose tolerance, and insulin sensitivity [119, 120]. Other homeostatic gut bacterial species present in humans and rodents, like *Lactobacillus (Lactobacillus sakei, Lactobacillus mucosae)* and *Bifidobacterium (Bifidobacterium adolescentis, Bifidobacterium animalis)* regulate the host immune response and have antiobesity actions [121]. *Lactobacillus sakei, Lactobacillus mucosae*, and *Bifidobacterium adolescentis* attenuate gut inflammation, obesity, and hepatic steatosis by producing anti-inflammatory IL-10 [121]. The gut microbes also metabolize host-dietary lipids to generate lipid metabolites. The metabolism of lipids such as omega-3, cholesterol, and omega-6 by *Bifidobacterium*, and *Lactobacillus* generates lipid intermediates that promote anti-inflammatory functions, limit oxidative stress that helps to maintain gut homeostasis, and regulate GLP-1 release, influencing glucose homeostasis [122]. The microbial products also regulate enterocyte function; for example, *L. paracasei*-produced lactate inhibits chylomicron secretion and fatty acid oxidation via malonyl-CoA generation, whereas acetate produced by *E. coli* promotes fat oxidation via the AMPK/PGC-1α/PPARα pathway and inhibits chylomicron secretion [123]. The gut microbiome species present in

the normal homeostatic gut, such as *Akkermansia*, also impacts the endocannabinoid system affecting food intake and satiety. *Akkermansia muciniphila*, can also generate a diacyl phosphatidylethanolamine lipid species that can dampen the proinflammatory IL-12/IL-23 response in human monocytes [122]. The roles of *Bifidobacterium longum* and *Bifidobacterium bifidum*, which affects OXPHOS in adipose tissue increase energy expenditure, improve glucose metabolism, reduce hepatic steatosis and regulate intestinal sterol biosynthesis [71] have also been elucidated with marked reduction in individuals with visceral obesity and fatty liver.

The gut microbiome species *Akkermansia muciniphila*, *Clostridium, Ruminococcus, Blautia, Escherichia coli K-12, Enterococcus* and *Lactobacillus* spp. (*Lactobacillus plantarum*) that produce gut serotonin to stimulate enteroendocrine cells, facilitating GLP-1 release to regulate peristalsis and regulate dietary lipid utilization by increasing jejunal lipid absorption rate [124, 125]. Serotonin is metabolized partially in enterocytes to indole-acetic acid, but large amounts of serotonin reach tissues such as the liver, where it promotes gluconeogenesis in fasting states and lipid accumulation in fed states [125]. In brown adipocytes, serotonin inhibits thermogenesis whereas in white adipocytes it promotes lipid storage [125]. In pancreatic tissue, serotonin promotes insulin release and in skeletal muscles, it promotes glucose utilization [125].

These evidence indicate that gut microbes can act directly or via their metabolites to impact host absorptive and gut homeostatic behaviour, thus any alteration in their relative populations might impair gut functionality.

2.4 Effect of dietary changes on gut microbes and their metabolites: The type of diet regulates gut microbial population diversity and strongly correlates with long-term dietary habits. A diet rich in proteins and animal fat increases *Bacteroides,* whereas a low protein and higher carbohydrate diet influences *Prevotella* populations [126]. Alteration in microbial diversity are observed in both short-term and long-term dietary changes as early as 24-48 hours of diet intervention; however long-term dietary pattern changes have more pronounced effects [127].

In cases of low-fat or isocaloric diet consumption, the relative abundance of Lachnospira *(Blautia), Alistipes,* and *Faecalibacterium* are increased, but the abundances of *Roseburia* and *Ruminococcus* are decreased [128]. The relative expansion of *Blautia* species results in the production of acetate and the inhibition of indole metabolite production [129] promoting gut homeostasis. The *Blautia* population was also negatively correlated with visceral fat accumulation in a study conducted among Japanese people [130] and dietary intervention in these obese individuals with a low-calorie, high-protein diet for 3 weeks increased *Blautia* populations [130].

The consumption of a plant- or animal-based diet also induced changes in microbial diversity, with a plant-based diet increasing abundance of *Bifidobacterium* species that enhance glycemic control, possibly due to the action of plant sterols [130]. Long-term plant-based diet consumption results in increased SCFA producers namely *Prevotella* and *Bacteroides* with increased *Prevotella/Bacteroides* ratios, due to increased fibre content [130].

The consumption of substantial amounts of glucose or fructose can also effectively alter the gut microbiome, via the inhibition of *Bacteroides thetaiotamicron*, which prevents the colonization of harmful pathogenic microbes [130]. High fructose reduces the gut microbial populations of *Bifidobacteria*, *Lactobacillus*, *Bacteroides*, and *Ruminiococcus* [130]. The transition of a diet from low-fat to high-fat also induces changes in the gut microbial diversity, where more *Blautia* and *Faecalibacterium* groups were present in the low-fat diet group, whereas in the high-fat diet group, *Alistipes* and *Bacteroides* were increased along with a reduction in *Faecalibacterium* species [130]. In low-fat diet groups, the relative concentrations of tryptophan metabolites such as indole and p-cresols were low, which was negatively correlated with hypertension and cardiovascular disorders [130]. In high-fat diet consumption groups, bacterial populations producing lipopolysaccharides are observed in greater quantities, with a reduction in SCFAs [130]. The type of fat present in the diet can also specifically alter gut microbial populations, with polyunsaturated diets increasing the number of *Isobaculum* species. However, a monounsaturated fat-rich diet increased *Parabacteroides*, *Prevotella, Turicibacter,* and *Enterobacteriaceae* populations [130] in nonobese people, whereas in obese people

it increased *Parabacteroides* populations and decreased *Isobaculum* populations [130]. Therefore, we can safely assume that diet changes can independently induce drastic changes in gut microbial species, so understanding which specific species are altered in HFD or HCHF consumption might be beneficial in understanding microbe-host metabolism interactions.

2.5 High-fat diet-induced alterations in gut microbes and their metabolites: A high-fat diet or western diet reduces the relative population of the *Bacteroidales S24-7 group (Muribaculaceae)*, *Lactobacillus* [131], *Akkermansia muciniphila* [131,132], *Ruminococcaceae Ugg-014* [58, 59, 133-135], *Actinobacteria* [58, 134], *Faecalibacterium prausnitzii* [131, 133, 136], *Christensenella minuta* [137], *Bifidobacterium (Bifidobacterium longum* and *Bifidobacterium bifidum* [132, 138]), and *Blautia* [132, 139]. HFD or western diet also increases the populations of *Desulfovibrionaceae (Bilophila- Bilophila wadsworthia)* [132, 133, 136, 139, 140], *Rikenellaceae* [139], *Lachnospiraceae (Lachnospiraceae_FCS020 group)* [131, 135], *Clostridia (Clostridium_sensu stricto 1)* [135], *Erysipelotrichales* [57, 58], *Alloprevotella* [59], *Bacteroides* [59], and *Alistipes (Alistipi obesi)* [136, 140, 141] compared with rodents and/or humans on standard chow [58, 59, 142], with many of the altered species positively correlating with obesity development [133]. Another study has indicated that a high-fat diet increases relative populations of *Firmicutes, Erysipelotrichaceae* [133], *Proteobacteria, Romboutsia, Turicibacter, Lachnoclostridium, Blautia* [140], *Ruminococcus_torques* [136], *Sutterella, and Escherichia-Shigella* [58, 143]. However, some data also indicate that certain microbial species, such as *Actinobacteria, Deferribacteres, Verrucomicrobia, TM7, Cyanobacteria,* and *Tenericutes* remain unaltered in the context of HFD consumption [144]. Studies also indicate that in obese individuals, an increase in populations of *Bacteroidales,* such as *Lactobacillus (Lactobacillus reuteri), Bacteroides* spp., *Enterococcus* spp., *and Enterobacteriaceae species* are observed [137]. Some studies also indicate that with increasing obesity, relative populations of *Clostridia (Clostridium leptum), Enterobacter, Akkermansia (Akkermansia muciniphila), Faecalibacterium, Oscillibacter* (butyrate producers) [145], and *Alistipes* are reduced [137]. High-calorie diets can reduce populations of *Proteobacteria, Actinobacteria, Lactobacillus*, and *Muribaculaceae*, but the

increased abundance of *Lachnospiraceae*, *Bacteroidaceae*, and *Peptostreptococcaceae* lowering the production of short-chain fatty acids [146, 147].

The wide variation in gut microbial species identified in lean homeostasis guts or cases of high-fat/high-calorie diets stems from the fact that the gut microbial population changes with age, sex [148], number of calories, fasting or feeding gaps, type of diet and immune interactions, thus providing a uniform list of altered microbial profiles is very challenging. The gut microbiome diversity is directed not only by dietary factors but also by host metabolism or pathophysiological states. The study of microbes-diet interaction is widely varied among different reported results because of inter-individual variability in gut-microbial species. The inter-individual differences in microbes and their responses to dietary changes are due to host genomics/genetics which can also influence the host digestive behavior and microbial diversity [149-152]. One of the most common microbial species *Faecalibacterium* was found to be associated with blood group antigens and glucose levels thus indicating a much complex relationship between microbes and host genetics [152]. Future research should be focused on monozygotic and dizygotic twin studies to delineate host genetics and gut microbial signatures.

The reason for high-fat or Western diet-induced gut dysbiosis is not yet apparent, with some studies indicating that lack of fiber rather than a high-fat diet accounts for change in microbial diversity (gut dysbiosis) [153]. However, multiple studies have indicated that factors such as altered bile profiles or mitochondrial alterations may be responsible. One of the studies conducted in humans and rodents revealed that a high-fat diet combined with antibiotics impairs mitochondrial functions in colonocytes, limiting oxygen consumption and reducing gut anaerobicity [154]. High-fat diet consumption reduces colonic endothelial nitric oxide synthase (eNOS) enzyme expression and increases nitrite excretion, thus altering the oxidative balance [59], which may account for oxidative capacity changes in colonocytes. The anaerobicity of the gut is necessary to maintain normal gut

flora, however, reducing this may contribute to the initial phases of dysbiosis. However, scientific evidence is not sufficient at this point.

In rodents and humans, high-fat diet consumption increases luminal bile acid secretion because of its involvement in lipid digestion and absorption. High-fat diet consumption increased the levels of bile acids, namely cholic acid, which stimulated takeda-like G protein-coupled receptor-5 (TGR5) in Paneth cells in the GI tract and reduced the secretion of defensins-5 and -6 [155]. High-fat diet-induced cholic acid also upregulated endoplasmic reticulum (ER) stress, autophagy, and apoptosis in Paneth cells, impairing Paneth cell functions and reducing Paneth cell numbers in the ileum after 2-3 weeks of high-fat feeding [155]. The altered defensin production, coupled with a high-fat diet and high cholic acid-induced enrichment of amino acid metabolism pathways for the gram-negative cell wall (lipopolysaccharide) and reduction in gram-positive cell wall (peptidoglycan) synthesis, may play a role in the alteration of the microbial profile [155].

The degree of saturation of fatty acids in dietary TAGs metabolized by the gut microbiome also regulates microbiome composition, motility, and mitochondrial functions. A diet rich in saturated fats (high-fat diet/western diet) alters microbial populations, increasing *Firmicutes*, decreasing *Bacteroidetes*, and increasing *Fusobacterium, Lachnospiraceae,* and *Escherichia* [156]. Saturated fats also increase hydrogen peroxide production, impair enterocyte mitochondrial functions, and reduce oxidative phosphorylation (OXPHOS) [125, 157]. A reduction in OXPHOS lowers enterocyte oxygen consumption and limits the luminal hypoxia necessary for obligate anaerobic microbe maintenance (in the lean gut), promoting facultative anaerobe growth and altering microbial diversity [158]. Studies have demonstrated that high-fat diet consumption decreases the production of indoles and increases kynurenine metabolites [159], primarily by altering the gut microbial populations of *Bacteroides thetaiotaomicron*, *Clostridium* spp., *Proteus vulgaris*, *Enterococcus faecalis*, and *Bifidobacterium,* which negatively impacts *Bacteroides* spp., *Clostridium, Eubacterium,*

Bifidobacterium and enhances *Escherichia coli*, further reducing the production of indoles and derivatives whereas a high-fiber diet promotes SCFA production in the opposite manner [159-165] .

The consumption of a high-fat diet can dysregulate the gut immune system via an alteration of SCFAs (refer to Table 1 for SCFAs action on immune cell function) and indole derivatives, which impairs immune cell functions. One such impairment happens in the functionality of T-helper cells (Th cells) and T-regulatory cell (T_{reg}) disrupting Th17/T_{reg} balance [166]. High-fat diet-induced populations of *Prevotellaceae, Erysipelotrichaceae* [146], *Roseburia* [167], *Fusobacterium, and Escherichia-Shigella,* are high-intensity LPS producers, increasing gut luminal LPS concentrations [63, 143]. Dysregulated gut immune cells and increased intra-luminal LPS levels increase the likelihood of LPS-mediated inflammatory response [165,166].

High-fat diet intake for 16 weeks reduces the expression of *Mucin2*, and reduces the number of goblet cells, thus effectively reducing mucin production [134]. This reduction in mucin production allows the direct exposure of microbes to luminal epithelial cells, increasing LPS-mediated stimulation [115]. Microbial sterol metabolites also regulate T-cell homeostasis, maintaining homeostatic gut flora, however high-fat diet-induced altered metabolites dysregulate T-cell functionality, predisposing toward inflammation [77].

Western diets have low concentrations of omega-3 and omega-6 fatty acids, reduce populations of *Bifidobacterium, Lactobacillus* all of which increase gut oxidative stress and inflammation, impairing gut homeostasis [77]. In rodents, nitrite supplementation can ameliorate high-fat diet-induced oxidative stress by altering microbial population diversity by increasing *Lactobacillus* and reducing the abundance of *Bacteroidales S24-7 group, Alistipes* [59]. Nitrate supplementation also restores nitric oxide (NO) balance, impacting NO-cGMP dependent pathways and promoting white adipose tissue (WAT) browning [59]. Promoting WAT browning increases energy expenditure, which is obesity-preventive, and a reduction in NO by high-fat feeding reduces energy expenditure, increasing the degree of storage-induced metabolic dysfunction [59]. High-fat feeding reduces *Burkholderia*

species, *decreases* gut 5-HIAA production, and may be one of the plausible mechanisms of hepatic insulin resistance [118].

The consumption of a high-fat diet in humans and rodents, increases levels of conjugated deoxycholic acid metabolites and reduces normal bile metabolites (glycohyocholic acid and β-muriocholic acid), SCFAs (acetic acid, butyric acid, propionic acid and valeric acid) as compared with those in standard chow-fed rodents, indicating a collaborative interrelationship between diet, the microbiome, and bile acid metabolism [142, 168]. A high-fat diet and cholic acid independently also favor the expansion of specific microbial species, such as *Clostridium*, *Ruminococcaceae*, and *Lachnospiraceae*, better tolerators of high-biliary levels [155]. High luminal cholic acid levels can also independently inhibit *Lactobacillus* populations, which can alter the secondary bile acid profile, increasing the bile acid pool via dysregulation of FXR signaling [155], indicating at bile acid-microbiota communication.

Intestinal immunoglobulin-mediated immune responses generally regulate the impact of microbial lipid-driven absorptive behavior, preventing excessive lipid absorption [122]. However, high-fat diet-induced alterations in immunoglobulin profiles account for excessive lipid absorption [122] which then affects mitochondrial respiratory capacity.

In high-fat diet-induced rodent obesity models, the level of intestinal serotonin production was increased, which was inhibited by *Bacteroides vulgatus* [169]. Elevated gut luminal serotonin levels increases lipid absorption rates and also impacts serum serotonin levels, inducing neurological effects such as anxiety and neuroinflammation [169]. In addition to this, studies have also elucidated that microbiota may play a role in host lipid absorption since germ-free rodents fed a high-fat diet show reduced lipid digestion and absorption [170]. A high-fat diet can also alter populations of bacterial species like *Clostridiales* and *Clostridium (*that positively correlate with body weight) and *Bacteroides thetaiotaomicron* (in the human gut) that increases lipid digestion, absorption, and deposition promoting obesity, hyperglycemia [63, 171].

One study done in Mexican subjects with T2DM and MetS demonstrated an increase in microbial species like *Erysipelatoclostridium*, *Shaalia*, and *Actinomyces,* which reduces gut hypoxia, facilitating the growth of harmful bacteria that generate pathogen-associated molecular patterns, stimulating immune reactions [63]. HFD induces bacterial species like *Oscillibacter, Desulfovibrio, and Enterococcus faecalis* that increase luminal reactive oxygen species (ROS) production, inducing enterocyte apoptosis and mitochondrial dysfunction [172]. HFD increases gut oxidants which along with LPS induces proinflammatory cytokine production, which induce TDO (via NF-κB and C/EBPβ) and IDO (via Jak-STAT) [173], leading to increased kynurenine production which then interacts with AhR to stimulate IDO expression further potentiating kynurenine production. The gut luminal elevation of LPS alters the gut immune system via transmigration into the sub-epithelium through disrupted gut barrier, indicating that gut barrier integrity plays an important role in maintenance of normal homeostasis. The gut microbes also play a role in maintaining gut barrier integrity wherein lean gut predominant bacterial species like *Lactobacillus*, *Bifidobacterium, Bacteroidetes*, *Clostridiales*, and *Akkermansia muciniphilia* augment barrier integrity [134, 174]. High-fat diet reduces *Akkermansia muciniphila, Bifidobacterium* populations which then downregulates the expression of tight junctional proteins, altering gut junctional integrity, allowing the transmigration of LPS into general circulation [134]. High-calorie diets rich in sucrose or fructose consumption can also alter the microbial profile, increasing *Proteobacteria*, *Escherichia, Firmicutes,* and *Enterobacteriaceae* and reducing *Akkermansia, Bifidobacterium* impairing gut barrier integrity [174].

The gut microbiome not only regulates local gut homeostasis but also influences systemic pathways like insulin resistance/sensitivity and body weight regulation. A microbial species, *Prevotella copri* (*P. copri*), increases upon exposure to the Mediterranean diet, and promotes weight loss and insulin sensitivity. Similarly, *P. copri* on a Western diet is reduced, increasing weight gain and insulin resistance [63]. Another microbial species namely, *Clostridium* increased in HFD consumption, can influence the metabolism of branched-chain amino acids, increasing their serum concentrations,

which can independently correlate with insulin resistance [175]. The consumption of a Western diet (rich in saturated fats) increases *Clostridium* and *Enterobacteriaceae*, both of which can convert choline to trimethylamine (TMA) or trimethylamine N-oxide (TMAO), promoting cardiovascular dysfunction [158].

The gut microbial species also regulate the relative populations of each other. Microbial species such as *Clostridium butyricum* (butyrate producers) are abundant in the lean gut. However, the administration of a high-fat diet reduces *Clostridium butyricum* populations [176], which affects other butyrate producers like *Lachnospiraceae and Ruminococcaceae* [177]. The administration of *Clostridium butyricum* as a probiotic in rodents alleviates high-fat diet-induced obesity by increasing the levels *of Ruminococcaceae* and *Lachnospiraceae* (only in males) [177]. Obesity associated metabolic disorders have often linked with microbial species such as nonalcoholic fatty liver disease (NAFLD) (*Bacteroides acidifaciens* and *Blautia producta*), obesity (*Lactobacillus, Bifidobacterium*, *Akkermansia*), diabetes mellitus type II (*Blautia, Akkermansia*), dyslipidemia (*Lactobacillus, Bacillus, Bifidobacterium*), hypertension (*Akkermansia, Blautia, Lactobacillus*), and hyperuricemia (*E.coli, Enterococcus*) [132].

The current research focused on the use of specific probiotics containing *Bifidobacterium*, *Akkermansia*, or *Lactobacillus*, have shown promising results concerning the attenuation of obesity or metabolic syndrome [156, 178]. Evidence indicates that *Akkermansia muciniphila* populations are drastically reduced in both human and rodent models of high-fat dietary intervention, and the introduction of Akkermansia species can attenuate aspects of metabolic syndrome [66, 67], highlighting the complex interplay of the gut microbiome and dietary composition in rodent models. Nevertheless, one of the significant drawbacks of probiotics is the use of different enterotypes of bacterial species, and many studies have not elucidated the complete mechanism by which such combinations work. Studies have also focused on fecal microbiota transplantation from nonobese to obese subjects. However, this topic is still controversial because of the different enterotypes

transplanted and inadequate knowledge of microbial interactions between the transplanted and native species.

Thus, we can deduce that maintenance of normal gut flora and gut-microbial metabolites form a highly interactive dynamic system maintaining normal gut health. HFD or HCHF dietary challenges initiate alterations in the gut microbial profile which can impact overall homeostasis (via altered gut-microbial metabolites) and intestinal lipid absorption thus paving the way for systemic maladaptation's. One of the major effectors regulating the human gut homeostasis is the enteric immune system, which involves maintenance of an intact gut barrier and an equally effective gut immunosurveillance and immune response. The gut is regularly exposed to a large variety of new pathogens via contaminated food, water or increased carbohydrate and lipid loads which are all capable of altering the gut microbial populations, makes enteric immune homeostasis a very challenging job. So, an understanding of gut-barrier integrity maintenance and gut immune homeostasis is important to elucidate high-fat and/or high-calorie diet-induced changes, since such alterations are corner-stones in understanding HCHF or HFD-induced inflammation, which can cause multiple metabolic dysfunctions.

3 Gut barrier integrity and homeostasis- A struggle to exist

Dietary constituents, allergens, foreign bacterial cells, toxins, and contaminated food constantly damage intestinal epithelial cells. The intestinal epithelial cells are continuously replaced weekly from the proliferation of stem cells (located in the crypt) stimulated by Wingless (Wnt) signaling (maintained by the CD-44 positive feedback loop) [179]. Replacing gut epithelial cells requires interplay among cell shedding (apoptosis), stem cell proliferation, and differentiation, followed by transit from the crypt base and gap resealing by adherens junctions and tight junctions. Stem and transit cells utilize glycolysis for energy, gradually shifting into fatty acid oxidation with OXPHOS during differentiation and maturation [180]. This shift in metabolism is supported by increased mitochondrial content, which generates ROS and induces epithelial cell senescence and apoptosis,

resulting in shedding [180, 181]. This repeated process of epithelial renewal is critical in maintaining gut barrier.

3.1 Homeostatic (lean) gut and barrier-integrity: The continuous cell layer in lean homeostatic gut allows selective movement of bioactive molecules or microbial byproducts. Many factors help to maintain gut barrier integrity, such as indole metabolites (via AhR), and immune cells (regulatory T-cells (T_{reg}), innate lymphoid cells-3 (ILC3)), and interleukin-22 (IL-22) act synergically via phosphoinositide-3-kinase (PI3K) pathways to stimulate intestinal epithelial cell proliferation, cell replacement, and resolution of damage (repair pathways) [182, 183]. The maintenance of junctional integrity also involves the maintenance of associated proteins like zonulins, occludins, claudins, cadherins to name a few. The lean gut microbes, via stimulation of host cytokines, also boost tight-junctional integrity directly or via its metabolites [181, 182]. Microbial degradation products like butyrate, indole metabolites (via AhR) upregulate the expression of junctional proteins like occludins, claudins, zonulin's; zonula occludens (ZOs) 1,2 and 3; cadherins [182, 185, 186]. The lean gut microbiota, like *Bifidobacterium* and *Akkermansia* also directly promotes junctional integrity [134, 174,186]. The integrity of the intestinal epithelium barrier is also augmented by mucus produced from goblet cells. Mucus production is upregulated by butyrate (via HDAC activity), IgA [187], and lean gut bacteria *Akkermansia* spp. [188]. Intestinal resident Paneth cells produce α-defensins, which are stimulated by butyrate [189], confer pathogen regulation and boost gut barrier function. The microbial degradation of bile acids generates secondary bile acids, which play a role in maintaining gut-tight junctional integrity (ursodeoxycholic acid in murine models [190]). All of these concerted efforts help to maintain an intact gut barrier limiting the exposure of harmful gut bacteria to underlying gut immune cells.

3.2 High-fat diet-induced gut barrier disruption: The consumption of a HFD increases lipid delivery into enterocytes, which induces ER stress, increases ROS production, downregulates tight junction protein expression, and alters junctional actin–myosin contractile properties [191]. Increased intracellular lipids in goblet cells induce oxidative stress, initiating the unfolded protein response

(UPR), and causing mucin misfolding [191]. The high-fat diet-induced reductions in butyrate, indole metabolite, and *Akkermansia muciniphila* populations impair mucin barrier functions. High-fat diet-induced initial disruption of gut barrier integrity allows a greater influx of luminal lipids to the lamina propria, which are transformed into lipid radicals [191]. These lipid radicals generate oxidative stress, impair enterocyte proliferation, disrupt occludin-zonulin-1 interactions, and cause basement membrane damage, creating gaps and increasing permeability [191]. A high-fat diet via microbial perturbation increases kynurenine production, which, along with increased ROS, induces mitochondrial dysfunction [191, 192]. ROS-induced DNA and mitochondrial damage initiate epithelial cell apoptosis, causing initial injury to the epithelial cell layer.

HFD consumption increases bile-tolerant bacteria, alters the bile acid profile, and increases intraluminal concentrations of deoxycholic acid (DCA) and chenodeoxycholic acid (CDCA) [193-195]. The altered secondary biliary metabolites disrupt tight-junctional dynamics [147, 191] via phosphoinositide 3-kinase (PI3K)– mediated zonulin-1 & E-cadherins phosphorylation or epidermal growth factor receptor (EGFR) mediated dissociation of the occludin-zonulin-1 complex [191, 196]. Disruption of junctional integrity further promotes the entry of DCA, CDCA, and LCA into the lamina propria, inducing ER stress and increasing ROS generation, which, along with HFD-induced oxidants [181], increases proinflammatory cytokine TNF-α production [197]. Proinflammatory cytokines impede cell cycle progression by inhibiting cyclin D-Cdk4 expression, preventing the transition from the G_0 phase and disrupting regenerative capacity. A HFD markedly reduces the levels of IL-17, IL-10 (by one week of high-fat diet ingestion) [191, 198], T_{reg} cells, and ILC3-IL22 [194], all of which are necessary for epithelial repair and regeneration. The impaired repair, coupled with HFD-mediated downregulation of junctional proteins, creates a gap in the epithelial linings, increasing intestinal hyperpermeability [191]. The continuation of a high-fat diet propagates continuous damage, accompanied by a failure to repair, leading to a leaky gut [181], allowing the transmigration of gut luminal LPS into the lamina propria, and stimulating the enteric immune system.

4 Gut immune maintenance: A Sentinel

The gut also has a robust immune system that constantly communicates with gut microbial species, regulating immune responses ably supported by microbial products such as SCFAs and indole metabolites.

Maintenance of normal gut immune response: The normal homeostatic gut has a variety of immune cells, such as T-cells, innate lymphoid cells (ILCs), B-cells, and antigen-presenting cells (APCs) that include microfold-cells (M-cells) and dendritic cells. The establishment of neonatal gut microbial population is dominated by SCFA producers influenced by maternal effects and breast milk sets the tone of setting up the enteric immune system [199, 200]. SCFAs producers in the gut modulate and regulate immune cell activation, differentiation, chemotaxis, and recruitment of neutrophils (via HDAC action and regulation of Chemokine (C-X-C motif) ligand -1 and -8 (CXCL1 and CXCL8 chemokines)) [201]. SCFAs can also inhibit monocyte, dendritic cell, and macrophage maturation, impede their antigen capture potential and lower proinflammatory cytokine (TNF & IL-12) production [202, 203]. In the normal homeostatic gut, which is characterized by constant butyrate (SCFA) production, it helps direct T-cell differentiation toward regulatory T (T_{reg}) cell populations [204]. Butyrate acts on dendritic cells via GPR109A receptors, alters tryptophan metabolism and exerts an immunosuppressive effect causing naïve T-cell conversion to FOXP3+ regulatory T cells rather than IFN-ɣ producing cells Th17 cells [204]. Butyrate also increases intestinal IL-10 production, reducing IL-6, promoting T_{reg} cell development and inhibiting proinflammatory Th17 cells [204]. Intestinal epithelial cells produce retinoic acid, which regulates dendritic cell populations that promote T_{reg} cells and promote gut integrity via ILCs (ILC3) [182]. Innate lymphoid cells (ILCs) are a group of cells that are currently identified as important regulators of barrier integrity. There are 3 types of ILCs namely group 1 ILCs (ILC1s and NK cells), which produce interferon-gamma (IFN-γ) and TNF, group 2 ILCs, which produce type 2 cytokines and group 3 ILCs, which produce IL-17, IL-22 [182]. Normal gut contains a high proportion of ILC2 maintained by intestinal epithelial cell-derived cytokines IL-25 and IL-33 that promote ILC2 proliferation facilitating mucous secretion

[182]. Lean gut also has relatively high populations of ILC3 (stimulated by SCFAs and indoles) which produce IL-22 [182], which increases goblet cell mucin production, the synthesis of antimicrobial peptides, epithelial stem cell regeneration, and the fucosylation of epithelial cells promoting barrier functions [205, 206]. IL-22 promotes antimicrobial REGIIIγ and REGIIIb production, and the fucosylation of epithelial cells which limits the growth of certain microbial species thus regulating gut microbial diversity [206, 207]. The neonatal gut undergoes significant changes during initial years of life when the gut develops a tolerance to homeostatic gut microbes. The presence of commensal bacteria prevents the growth of harmful bacteria by nutritional competition [208], which coupled with gut anaerobicity helps in expansion of homeostatic gut bacterial populations. Gut-associated lymphoid tissue (GALT)-resident dendritic cells (DCs) produce IL-23 which in turn stimulates the release of noninflammatory IL-17 and IL-23 release from Th17 cells [207]. Homeostatic gut-resident microbial exposure to DCs activates them, which in turn stimulates innate lymphoid cell type 3 (ILC3) to produce IL-17 and IL-22 [209, 210] and also stimulate T-cell differentiation [211]. IL-17 promotes the expression of the tight junction protein occludin and antimicrobial functions of Paneth cells [207, 212].

DC-ILC3 interactions also promotes anti-microbial peptide and antibody productions which further regulates gut microbial populations [213, 214]. Antigen-capturing microfold-cells (M-cells) stimulate macrophages which in turn promote T cell differentiation into T_{reg} cells, and Th2 cells [182]. The lean gut has a high population of T_{reg} cells that modulate the function of DCS, ILC3s with additional influence from indole-AhR ligand interactions [215-217]. The importance of indole derivatives in the gut arises from their ability to ameliorate intestinal inflammation (via T_{reg} regulation), maintain the T_{reg}/Th17 balance, and suppress proinflammatory cytokine production and increase anti-inflammatory cytokine production [218].

Bile acids in the normal via TGR5 receptor binding promotes monocyte to tolerogenic dendritic cell differentiation and macrophage polarization toward the M2 phenotype [219]. Bile acids via FXR binding inhibit inflammasome activation, promote IL-10 secretion and inhibit proinflammatory

cytokine production [219]. Homeostatic gut microbes generate secondary bile acids which bind to FXR to promote T_{reg} differentiation and prevent caspase-1 and NLRP3 inflammasome assembly [210, 219]. Bile acids also suppress neutrophil transmigration (along with SCFAs) [219], inhibit Th1 differentiation via vitamin D-receptor (VDR) and regulate the Th17 response (delicate balance) which is essential for normal epithelial regeneration due to Th17 mediated IL-17 release [220]. Normal bile acids such as cholic acid via TGR5 inhibit NLRP3, improve gut insulin sensitivity, facilitate mucin production and secondary bile acids inhibit Th17-cells, induce Treg accumulation [221]. Normal lean gut also has a high population of ILC2 maintained by bile acid metabolites, indoles [215, 221, 222]. Eosinophils, another variety of immune cells present in the homeostatic gut help in immune regulation via its interaction with other immune cells via IL-5, -6, -13 and transforming growth factor-beta (TGF-β) [223], maintain barrier integrity by increasing mucus secretion [224, 225] enhancing tissue repair [223]. Eosinophils also aid in destroying parasites/helminths via the production of eosinophil-derived antimicrobial proteins and enzymes and IL-5 [223, 224] thus maintaining gut homeostasis. In the homeostatic gut, B-cells express IL-10, facilitating T_{reg} cell differentiation and inhibiting the production of proinflammatory cytokines [226] (refer figure 2 for normal gut immune cells and their homeostasis).

Figure 2: Maintenance of lean homeostatic gut immune responses and barrier integrity

Figure 2 Legend: Commensal bacteria stimulate IEL-CD+T cells to promote antimicrobial peptide production which regulates microbial populations. γδ-IELs mediate immune surveillance which helps to maintain gut homeostasis via secretion of KGF and IL-22. KGF promotes epithelial repair and regeneration. Normal anaerobic bacteria, and their products stimulate dendritic cells (DCs) in Peyer's patches. Activated DCs stimulate ILC3 to produce IL-17and IL-22. IL-17 and IL-22 help maintain gut barrier integrity and promote anti-microbial REGIIIγ production (via Paneth cells) promoting immune tolerance and regulate microbial populations. Activated dendritic cells stimulate IFNγ+ CD8+ T-cell differentiation into activated T cell, memory T cell, and cytotoxic T cell subsets. Antigen presentation via M-cells stimulates macrophages which then with DCs, and additional inputs from SCFAs, dietary

retinoids activate regulatory T cells (FOXP3+). Normal lean guts have high T_{reg} cells population which inhibit Th1 cells (via TGF-β), regulate Th17 response and promote the Th2 response (anti-inflammatory) maintaining overall healthy immune response. The lean gut has high concentrations of ILC3 stimulated by antigen-DC interaction, T_{reg} cells, butyrate and indole-AhR interactions, which help resolve inflammation and gut barrier homeostasis via IL-22 release. In contrast, follicular–T cells (promoted by the gut microbiota via CD4+ T-cell) promote the B-cell mediated release of immunoglobulins to regulate the gut microbial flora. Normal gut bile acids facilitate mucin production and inhibit Th17-cells, induce Treg accumulation, thus maintaining healthy gut conditions. SCFAs also promote mucin production via histone deacetylase (HDAC) and MAPK/PTEN pathways. Antigen sensing by EECs also stimulates ILC2 to resolve inflammation.

IEL-CD8+T-cells: intraepithelial lymphocyte CD8 T cells, γδ-IELs: γδ-intraepithelial lymphocytes, ILC: innate lymphoid cells (three types ILC1, ILC2 and ILC3), KGF- keratinocyte growth factor, Tfh: Follicular T-cells. [Figure 2 legend ends]

4.1 HFD and gut immune dysregulation: Compared with isocaloric or low-fat fed lean gut individuals, diets that induce obesity or metabolic syndrome are generally rich in high amounts of fats (saturated animal fat) with/without high–carbohydrate and low dietary fibers. Such changes in dietary composition transforms microbes from obligate to more facultative anaerobes with poor diversity induces GALT to mount an immune response. High-fat diet consumption increases LPS producing microbes which coupled with reduced duodenal brush border alkaline phosphatase (ALP) activity (dephosphorylates inactivating LPS) [227], increases intraluminal concentration of LPS. HFD-induced leaky gut facilitates LPS to migrate into gut lamina propria via micelles, activating gut resident immune cells and then traveling to the systemic circulation [228], inducing systemic immune activation.

A high-fat diet upregulates gut epithelial TLR4 receptors, which binds LPS and free fatty acids, producing proinflammatory cytokines TNF-α and NF-κB from intestinal macrophages [229]. High-fat diet induced alteration in microbiota produces more hydrogen sulfide (H_2S) [230], which inhibits

complex IV activity and mitochondrial proteins impeding mitochondrial OXPHOS. Inhibition of OXPHOS generates ROS, inducing mitochondrial dysfunction and mitophagy, which affects cell proliferations [231]. The LPS-TLR4 interaction upregulates the production of proinflammatory cytokines such as TNF-alpha [229], and NO [232]. NO causes protein S-nitrosylation, leading to ER stress and epithelial cell renewal failure [233]. A HFD-induced microbial alteration reduces IL-33 production which depletes intestinal eosinophils [234].

High-fat diet-induced inflammation depletes ILC3 through the transition to ILC1 via increase in IL-23 and IL-1β [235]. Transitioned ILC1 secrete more proinflammatory cytokines, such as IL-22, IL-17 and IFN-γ [235]. IL-17 overexpression recruits neutrophils which disrupt E-cadherins and junctional adhesion molecules leading to elevated gut permeability [143, 235]. ILC1-secreted GM-CSF recruits inflammatory monocytes, initiates inflammation and aids in neutrophil migration, thus modulating inflammation [235]. HFD also can impairs the function of ILC3s via lipotoxicity due to elevated lipid uptake by enterocytes [236]. The hypercaloric diet-induced expansion of ILC1 cells correlate with a reduction in the microbial species of *Akkermansia muciniphila* and expansion of *Bilophila* spp., thus indicating a bidirectional relationship [237]. ILC1 expansion also activates inflammatory signals, increases proinflammatory cytokines, dysregulates gut hormones and induces insulinemia [237]. ILC3 dysfunction and death may also be accentuated by hyperactive tissue-resident mononuclear phagocytes, which mark the initial events in gut inflammation in humans within hours of HFD intake [236].

The number of mucosal-associated invariant T-cells (MAIT cells) required for "necessary inflammation" and repair are reduced in intestine during obesity due to changes in microbial populations [238, 239]. The remaining MAIT cells exhibit an activated phenotype and produce the inflammatory cytokines IFN-γ, TNF-α, and IL-17 [238], causing excessive inflammation [240], impairing tissue repair and insulin resistance. A single-day HFD consumption can reduce the number of small intestinal intraepithelial lymphocytes (IELs) and small intestine–lamina propria lymphocytes

(LPLs) [241]. This reduction in IELs and LPLs may be caused by the lipotoxic effects of saturated and unsaturated fatty acids on these cells [241], impairing antimicrobial peptide production.

HFD ingestion alters the microbial profile that dysregulates microbe–APC interaction [242] and causes expansion of Th1 [243] and Th17 cells [244]. Activated Th1 and Th17 cells stimulate macrophages to release proinflammatory cytokines such as TNF-α, INF-γ, IL-2, and IL-17 [242]. The HFD-induced LPS–TLR4 interaction may also activate the NLRP3 inflammasome, resulting in the production of IL-1β [243]. High-calorie and high-fat (HCHF) diet consumption significantly reduces RORγt+Foxp3-negative Th17 cells and increases Th1 cell expansion [245]. Reduced butyrate or indole in high-fat diet consumption downregulates RORγt- or RORγt+Foxp3+ T_{reg} cells, skewing the balance in favor of an increased Th1/Th17 ratio [245]. The reduction in Th17 cells may also be attributed to the upregulation of *Erysipelotrichaceae* (*Faecalibacterium rodentium* [245]), *Ruminococcaceae*, and *Lachnospiraceae* via a reduction in IL-17 [245]. The reduction in butyrate-producing microbes, along with the transition toward kynurenine synthesis and an altered bile acid profile, all synchronously play a role in dysregulating the gut immune system and reducing IL-22 and IL-17.

In obese mice, IgA production in the colon, mesenteric lymph nodes, and Peyer's patches is reduced [219, 246]. The relative lack of IgA within gut-associated lymphoid tissue [246, 247] in diet-induced obesity may be one of the reasons for the altered gut microbiome in diet-induced metabolic abnormalities. HFD-induced gut barrier and immune disruptions facilitates entry of luminal LPS into systemic circulations via LPS-enriched extracellular vesicle [248], increasing serum LPS concentrations. LPS-TLR4 mediated IL-6, TNF-α activates NLRP3 inflammasomes (costimulated by saturated fatty acids), producing IL-1β, which activates CD8+ T cells and γδ-T cells, increasing IFN-γ production [249]. Systemic LPS activates the immune system producing a low-grade inflammation. HFD consumption increases disrupts intestinal Th1/Th17 balance, which subsequently may infiltrate

to non-lymphoid tissues, particularly adipose tissue, and contribute to the proinflammatory environment [250].

There have also been reports of bacterial metabolite imidazole propionate (a histidine-derived metabolite), which is increased in HFD consumption [251]. The gut-derived imidazole propionate can reach the liver via the portal vein, where it inhibits hepatic insulin signaling via mTORC1-dependent pathways [251].

Thus, we can conclude that high-fat with/without high-calorie diets alter enteric microbial species, their metabolites and immune disbalance that affects the systemic metabolism and immunity, inducing low-grade inflammation. The disrupted gut barrier integrity, along with gut microbial changes and compromised immune system allows, harmful gut-derived biomolecules, to enter into gut epithelium and then systemic circulation. Transmigration of such biomolecules (called pathogen-associated molecular patterns (PAMPs)) such as LPS activates the host immune system and creates an inflammatory environment (endotoxemia).

5 Adipose tissue: Utilization or Stockpile – A tricky conundrum

The process of absorption of dietary components culminates in their utilization and storage. Mammalian adipose tissues are major storehouse of fats which are utilized for energy during periods of starvation or energy deficit. Adipose tissues are divided into white adipose tissue (WAT), brown adipose tissue (BAT), and beige adipose tissue. WAT adipocytes utilize glucose for energy, converts glucose to fat (triglyceride), uptakes dietary TAG (received via chylomicron) and stores TAGs as lipid droplet. WAT is present in the subcutaneous (SAT) and intra-abdominal regions (VATs) [252]. BAT is present from birth in the supraclavicular, axillary, neck, periaortic, paravertebral, perirenal, and mediastinal regions [253]. Brown adipocytes utilize glucose and stored TAGs, dissipates energy through thermogenesis facilitating energy expenditure but decreases with age and remains around deeper organs [253]. Beige adipocyte proportions depend on energy metabolism and is directly proportional to energy expenditure [253]. The adipose tissue is not just an assembly of cells (adipocytes), but they have their niche environment with supporting cells that form the adiponiche.

5.1 Adiponiche: The adipose tissue contains mature adipocytes that store TAGs, the stromal vascular fraction (SVF) and immune cells such as T_{reg} (Foxp3+CD4+) cells, innate lymphoid cells (ILCs), macrophages (M2 polarized), and eosinophils [254]. The SVF consists of adventitial pericytes (CD45-CD31-CD146+), luminal endothelial progenitor cells (CD45-CD31+CD34+), supra-adventitial adipose stromal cells (CD45-CD31-CD146-CD34+), intermediate phenotype cells (CD146+CD34+) [215], fibroblasts, progenitor adipocytes, blood vessels, and extracellular matrix-associated proteins such as collagen [254]. Adipocytes, the SVF, and immune cells constitute adipose tissue microenvironment (adiponiche). The adipose tissue ECM interacts with the adipocyte membrane, stimulating the β1-integrin-ERK pathway, which affects the insulin receptor membrane caveolae, promoting insulin sensitivity [255]. The ECM also contains proteins such as collagen (types I, III, V, and IV), fibronectin, elastin, laminin, nidogen, perlecan, and proteoglycans [255]. Fibronectin-collagen I interaction regulates cytoskeletal organization and adipocyte function; elastin associated with collagen VI maintains adipocyte elasticity [255]. Laminin-collagen IV interaction forms the basement membrane, with additional support from nidogen and perlecan [255]. Proteoglycans such as heparin and chondroitin sulfate are essential for collagen organization and fibril formation [255]. The various components of the adiponiche supports adipocytes, which store and utilize fats making them important contributors in nutrient excess conditions.

5.2 Adipose tissue biogenesis: The formation of adipocytes, holds tremendous importance is terms of energy storage and utilizations, so the relative factors influencing them, opening new avenues for treatment strategies in nutrient excess situations. Adipose tissue biogenesis involves adipogenesis, which includes WAT, BAT and beige formation, white adipose tissue beiging, or the whiting of beige adipose tissue (influenced by adipogenic stimulators such as AMPK). WAT adipocyte differentiation occurs from myogenic transcription factor 5 (Myf5) -/+ progenitor cells dictated by peroxisome proliferator-activated receptor-gamma (PPAR-γ), transcription coactivator CCAAT/enhancer binding protein α or β (C/EBP- α or β), transcription factor-21 (TCF21), bone morphogenic protein-2 (BMP-2), BMP-4 via SMA and MAD related proteins (Smads) [256]. WAT adipogenesis is stimulated by

insulin, insulin-like growth factors (IGFs) via insulin receptor substrate-1 (IRS-1) or protein kinase B-mammalian target of Rapamycin (Akt-mTOR), and is inhibited by AMPK, wingless (Wnt)- beta-catenin, and sonic-hedgehog (Shh) pathway [256]. BAT adipocytes originate from Myf5+ progenitors influenced by BMP-7 [253, 256]. In contrast, beige adipocytes originate from the transdifferentiation of existing white adipocytes by signals such as cold exposure, β3-adrenergic agonists, PPARγ agonists, fibroblast growth factor-21 (FGF21), irisin, and natriuretic peptides but revert to WAT phenotype upon the removal of stimuli [257]. Beige adipocytes express markers for white and brown adipocytes but during white to beige (brown) transition, express brown adipocytes related uncoupling protein-1 (UCP1), a process called beiging [258, 259]. Beiging is associated with increased energy expenditure influenced by environmental, genetic, pharmacological, or physiological signals, such as β-adrenergic stimulation, diet, or exposure to cold [259, 260]. In cases of nutrient excess, WAT beiging is one of the protective mechanisms, with the help of beiging inducers like adipose tissue endothelial cells (AdECs) released vascular endothelial growth factors (VEGF) [257,259, 261, 262]. Other factors that induce beiging include endocytotic TAG degradation by lysosomes, cold exposure, and sirtuin 1 (SIRT1) activation [261, 263]. Factors inducing beiging, stimulate PPAR expression to increase beta-oxidation rates and promote thermogenesis and mitochondrial biogenesis via positive regulatory domain-containing protein-16 (PRDM 16) and peroxisome proliferator-activated receptor-γ coactivator 1-alpha (PGC-1α) [259, 264].

Other than adipocytes, different cellular subtypes identified from human adipose tissue samples include progenitor or stem cells, immune cells, and endothelial cells.

5.3 Adipose tissue endothelial cells and their importance: Adipose tissue endothelial cells that are present around the adipocytes are important regulators of adipocyte biogenesis and functionality. Endothelial cells are subdivided into three types, namely types 1, 2 and 3. Endothelial type 1 cells express genes involved in lipid metabolism and handling (FABP4, lectin galactose binding soluble 1 (LGALS1), CD36, retinol binding protein 7 (RBP7), glutathione peroxidase 3 (GPX3)) [265]. Type 2 cells express endothelial cell markers such as vascular cell adhesion molecule-1 (VCAM1),

intracellular adhesion molecule-1 (ICAM1), von-Willebrand factor (VWF), and tissue factor pathway inhibitor-1 (TFP1) [265]. Type 3 cells express the lymphatic endothelial cell marker lymphatic vessel endothelial hyaluronan receptor-1 (LYVE1) and are predominantly present in visceral white adipose tissue [265]. The endothelial cells (AdEc) lining the blood vessels (which express platelet-derived growth factor) maintain physical proximity with adipose stem cells. Endothelial and stem cell crosstalk occurs via VEGF, adipokines, and physical contact (upregulating Wnt signaling) to regulate adipocyte development and function. In lean, homeostatic adipose tissue, the vasculature is lined by a single layer of endothelial cells quiescent in the metabolic profile. In the presence of adipocyte-derived angiopoietin-2 (Angpt-2), VEGF, leptin, adiponectin these endothelial cells rapidly switch into an angiogenic or proliferative profile [265, 266]. Adipocyte-lined endothelial cells utilize glucose via GLUT-1–mediated uptake followed by glycolysis and the TCA cycle for ATP generation [265, 267]. The dependence on OXPHOS is low, allowing faster energy acquisition and facilitating tissue oxygen delivery and FATP4-mediated transendothelial transport of fatty acids [265, 267]. These endothelial cells utilize glutamine to fuel the TCA cycle with FAO-generating acetyl-CoA which is required to maintain vascular homeostasis, angiogenesis, and normal function [267, 268]. In a well-fed state, FFA from CMs or VLDLs supports endothelial cell functionality [267], indicating a symbiotic metabolic interrelationship between adipocytes and endothelial cells.

5.4 Homeostatic adipose tissue metabolism: The metabolism of adipocytes holds tremendous interest, in the maintenance of normal body weight and improvement of adiposity. During fed states, mature adipocytes hydrolyze TAG from CMs via AdEC-anchored glycosylphosphatidylinositol-anchored high density lipoprotein binding protein 1 (GPIHBP1) associated lipoprotein lipase (LPL) [269]. LPL releases FFAs, which are taken into endothelial cells via CD36 and fatty-acid binding proteins (FABPs) both of which are PPAR-γ regulated, stimulated by Angpt-2 [270–273] or via CD36- Jun N-terminal kinase (JNK)-caveolae mediated exosomes [274, 275]. FFAs are intracellularly bound to FABP 4/5 to maintain FFA entry flux with VEGF upregulating FATP4 [276]. VEGF enhances fatty acid uptake [271] and transendothelial lipid transport into adipocytes via FATP4 and CD36 [271,

277]. FFAs are then compartmentalized to either the ER (for TAG conversion for lipid droplet storage) or the mitochondria for oxidation (FAO) [278].

Dietary glucose enters adipocytes via insulin sensitive GLUT-4 transporter, whose activity is stimulated by insulin (via NO) and insulin-like growth factors (IGFs) [279]. NO also reduces leukocyte recruitment and accumulation and promotes insulin sensitivity [279, 280].

5.4.1 WAT metabolism: Glucose is metabolized to pyruvate, which enters the TCA cycle via acetyl CoA for energy production. TCA-generated reducing equivalents enter the electron transport chain (ETC). ETC activity alters the NAD/NADH ratio that activates SIRT-1, which increases mitochondrial mass, nutrient oxidation, and ATP generation [281]. AMPK activation by adipokines and SIRT1 activates PGC-1α to promote mitochondrial biogenesis, reduce oxidative stress, and boost ETC capacity [281, 282]. Small amounts of glucose-derived pyruvate are diverted towards lactate by insulin-FOXK1/2 signaling pathways, which promote endothelial fatty acid uptake [283-285]. Glucose flux is large, allowing it to enter HMP to produce NADPH needed for reductive biosynthetic pathway such as *de novo* lipogenesis [286] (refer figure 3).

Glucose enters the TCA cycle via acetyl CoA, to form citrate which is exported back to cytosol via citrate carrier in cases of nutrient abundance. In the cytosol, citrate is acted upon by the enzyme ATP-citrate lyase (ACLY) which generates acetyl CoA in cytosol [286]. Acetyl CoA is then converted to malonyl CoA by the enzyme acetyl CoA carboxylase (ACC) which is stimulated by cytosolic citrate [286]. Following this, acetyl CoA condenses with multiple molecules of malonyl CoA via the enzyme fatty acid synthase (FASN) complex to generate palmitic acid (C16). FASN activity requires NADPH which is supplied by HMP shunt pathways [286]. Palmitic acid can be converted to other saturated fatty acids such as stearic acid (C18) via fatty acid elongase enzyme systems [286]. Palmitic acid can also be converted into unsaturated fatty acids such as oleic acid (C18:1, n-9) by desaturase enzyme namely stearoyl-CoA desaturase (SCD) [286]. Insulin-mediated glucose uptake and insulin receptor signalling also promote the activity of ACLY to promote lipogenesis [286]. Glucose entry into adipocytes stimulates carbohydrate-response element-binding protein (ChREBP), which then

stimulates *de novo* lipogenesis [286]. Feeding stimulated insulin secretion also acts on adipocytes to stimulate sterol regulatory element-binding protein (SREBP)-1c which stimulates lipogenic enzymes such as ACLY, FASN and SCD [286]. The actions of ChREBP and SREBP1c are complementary to each other [286]. Insulin also activates liver X receptor (LXR), which is stabilized by glucose levels, also promote lipogenesis [286]. The fatty acids synthesized in adipocytes and fatty acids obtained via chylomicron increase FFA concentrations in adipocytes, which are converted into TAG to prevent lipotoxicity. TAG biosynthesis requires enzymes like glycerol-3-phosphate acyltransferase (GPAT), phosphatidate phosphatase lipin 1(LPIN1) and diacylglycerol acyltransferase (DGAT) [286]. The synthesized TAG is then stored in the form of lipid droplets (LDs) that require participation of proteins called perilipins (PLIN). There are multiples variants of PLINs namely PLIN1–PLIN5, with PLIN1 regulating fasting lipolysis, and PLIN3 participating in LD biogenesis [286]. PLIN5 is associated with BATs where they regulate LD degradation to obtain energy. Given the fact that adipocytes can obtain TAGs from fatty acids derived via *de novo* lipogenesis or dietary chylomicrons, studies reveal that majority (around 80-90%) of TAGs that are in LDs are composed of fatty acids derived from CMs [287]. This begs the question that what is the importance of *de novo* lipogenesis in adipocytes?

Studies have revealed that the process of de novo lipogenesis generates lipid signalling molecules play a major role in regulating autophagy that supports adipose tissue homeostasis [287]. The presence of cytosolic acetyl CoA in de novo lipogenesis promotes the acetylation of histone that plays a role in epigenetic regulation of lipogenic enzymes and facilitates insulin signaling via Forkhead box 1 (FoxO1) protein regulation [288]. Acetylation of histones may also play a role in maintaining cell death-inducing DFF45-like effector proteins -C (CIDEC) activity that stabilizes lipid droplets and promotes insulin sensitivity [287]. Malonyl CoA generated during lipogenesis promotes malonylation of multiple proteins involved in glucose and lipid metabolism [288]. In BAT, malonylation is seen more when compared to WAT in mitochondrial proteins under homeostatic conditions indicating that

malonylation of mitochondrial proteins improves their functionality [288]. In genetically obese rodents, WAT malonylations levels are significantly reduced on mitochondrial proteins [288].

Branched-chain amino acid delivery and insulin-RTK-Akt activation during the fed state activate mTOR signaling cascades [277], stimulating protein synthesis and upregulating SREBP1 expression for sterol synthesis. mTORC1 in WAT regulates adipocyte precursor commitment, adipogenesis, TAG synthesis, and mobilization [289]. mTORC1 regulates thermogenic gene expression and mitochondrial function in BAT and beige adipocytes [289]. *De novo* lipogenesis in WAT improves glucose metabolism and prevents lipotoxicity by stimulating adipogenesis [290]. Activated CIDEC protein regulates lipid droplet size, producing a single large lipid droplet in WAT [291, 292]. Thus, in well-fed state, WAT adipocytes store TAG (synthesized from CM-derived FA) into a single large lipid droplet.

5.4.2 Brown and beige adipocyte metabolism: In brown adipose tissue and beige adipocytes, well-fed metabolism mirrors white adipocytes in terms of chylomicron-mediated FFA delivery and insulin-GLUT mediated glucose uptake. Glucose metabolism via glycolysis and TCA produces NADH which enters ETC but cannot produce ATP due to the presence of uncoupling protein-1 (UCP-1), which promotes thermogenesis. UCP-1 is expressed early during development of brown adipocytes influenced by CREB, C/EBP, PGC-1α, PPAR-γ and cold exposure [293, 294]. In thermoneutral states, multiple factors like leptin, irisin, sympathetic stimulation (via cAMP-PKA) [293, 294], beta-adrenergic receptors, acetylcholine, natriuretic peptides, intrinsic long-chain fatty acids (>8C) in BAT, and cell death-inducing DFFA-like effector A (CIDEA) [295] all promote thermogenesis. In brown and beige adipocytes, glucose also produces acetyl CoA which feeds into *de novo* lipogenesis, which play important roles in BAT and beige functionality. In thermoneutral states, endothelial-derived stem-cell factor, regulates BAT functions via stem-cell factor receptor (c-Kit), which promotes *de novo* lipogenesis and TAG synthesis, which generates palmitoleate (a lipokine), that improves insulin sensitivity, and regulates adipose hypertrophy and macrophage differentiation [296, 297]. Palmitoleate (C16; 1) formed during *de novo* lipogenesis also regulates CIDE protein function,

producing small, multilocular lipid droplets and activating thermogenesis in BAT [295]. In BAT, *de novo* lipogenesis also provides fatty acids for FAO, which promotes thermogenesis [298, 299] whereas in beige adipocytes, *de novo* lipogenesis generates fatty acids that act as endogenous PPAR-γ ligands promoting WAT beiging [300]. The induction of UCP-1-mediated thermogenesis promotes mitophagy, influencing mitochondrial biogenesis [301, 302], which improves cellular thermogenic capacity and energy expenditure.

In beige adipose tissue, thermogenesis can also occur via a UCP-1 independent mechanism. UCP-1-independent pathway is stimulated by the bile-acid receptor TGR5 in an ERK/DRP1-dependent [259] mechanism. The UCP-1 independent mechanism involves increased expression of SERCA2b or RyR2, increasing cytosolic Ca2+ that increases ATP production [301]. The ATP produced converts creatine to phosphocreatine, thus effectively reducing the ATP/ADP ratio and promoting greater nutrient oxidation [301]. The conversion of white to beige adipocytes is one of the protective mechanisms, in nutrient excess as it helps to increase energy expenditure. Beige adipocyte expansion can occur via three mechanisms: *de novo* beige progenitor adipogenesis, the beiging of white adipocytes, and the proliferation of adult beige adipocytes [303]. The beiging of WAT is stimulated by multiple factors such as fasting, exercise, cold stimulus, and PPAR-γ [304]. Beiging of WAT involves the upregulation of UCP1, mitochondrial synthesis and the conversion of lipid droplets from unilocular to multilocular [304]. Recent evidence also suggests that immune mediators such as eosinophils and cytokines (IL-4, IL-13, and IL-5) produced from the lean homeostatic gut promote beiging via M2 macrophages and that thermogenesis promotes M2 polarization indicating a bidirectional signaling [305, 306].

Lipogenesis in mammalian WAT and BAT produces fatty acyl esters of hydroxy fatty acid (FAHFA) [307, 308], especially 5-PAHSA and palmitic acid esters of hydroxy stearic acids (PAHSAs)) namely 9-PAHSA variants which enhance insulin sensitivity (only by WAT derived FAHFAs) by promoting GLUT4 translocation and regulate adipogenesis [309, 310]. In BAT, cold exposure or exercise

increases 12,13-dihydroxy-9Z-octadecenoic acid (12,13-diHOME) production, which promotes FA transporter (CD36) translocation, increasing FA uptake for thermogenesis and WAT browning [311].

5.4.3 Lipid droplet hydrolysis: The breakdown of lipid droplets, is utilizes the same set of enzymes in white, brown and beige adipocytes. The lipid droplet-resident TAG is hydrolyzed by adipose tissue triglyceride lipase (ATGL), hormone-sensitive lipase (HSL), and monoglyceride lipase (MGL) to produce free fatty acids [312]. Fasting upregulates angiopoietin-like protein 4 (ANGPTL4), which inhibits LPL and reduces TAG delivery [313-315], which, along with lowering GLUT 4-mediated glucose delivery, limits *de novo* lipogenesis and TAG accumulation. Fasting-associated elevations of glucagon inhibit SREBP1c (286) with relative absence of insulin, ChREBP the enzymes of *de novo* lipogenesis namely ACLY, ACC and FASN are inhibited (286). Fasting-associated glucagon promotes CLD hydrolysis in adipocytes via the coordinated action of ATGL, HSL, and MAG lipase, which releases FFAs utilized by adipocytes and other tissues, such as the liver and muscles. Moreover, lipolysis-generated arachidonic acid can be utilized for 12-hydroxyeicosatetraenoic acid (12-HETE) synthesis, which enhances GLUT4 translocation, improving insulin sensitivity [309, 316, 317]. Fasting also promotes polyamine secretion by endothelial cells, stimulating adipocyte lipolysis via the Akt–mTOR pathway, fueling endothelial fatty acid β-oxidation (FAO), and promoting vascular growth maintenance [318]. Maintaining lipid droplet size requires a concerted effort and balance between lipogenesis and lipolysis; any failure causes devastating consequences as seen in HCHF or HFD diet consumption. (Refer Figure 3 and 4 for white adipocyte and brown adipocyte metabolism)

Figure 3: Major adipocyte metabolic adaptations in white adipocytes in lean and high-fat fed conditions (normal homeostatic pathways are marked in black, whereas HCHF- or HFD-fed altered pathways are marked in red, red arrows indicates pathways or metabolites reduced or increased in HCHF- or HFD-fed conditions)

Figure 3 Legend: Lipids delivered via chylomicrons or VLDLs upon entry via CD36 and FATPs are compartmentalized for lipid droplet storage or utilization by oxidation. Carbohydrates like glucose enter adipocytes via GLUT-4, participating in glycolysis and the TCA cycle followed by ETC to

generate ATP. TCA cycle generated NADH oxidation upregulates sirtuin 1 (SIRT1) which improves mitochondrial functions. Excess acetyl CoA is diverted to lipogenesis producing FFA, which are esterified to TAG for storage contributing to lipid droplet expansion. *De novo* lipogenesis generates FAHFAs which promotes insulin sensitivity. HCHF or HFD-fed caloric excess promotes greater entry of chylomicron-derived FFAs to adipocytes. Initial glucose-excess promotes DNL, inducing greater synthesis and storage of fats, which inhibits FAO promoting increased TAG storage and lipid droplet (size) expansion. Increased lipid droplet size and increased FFAs promote insulin resistance which reduces glucose entry and DNL. Lipid droplet expansion induces hypoxia, which along with saturate fatty acid induced ER or mitochondrial stress induces white adipocyte death and fibroinflammatory changes.

DNL: de novo lipogenesis, SFA: saturated fatty acids, SIRT1: sirtuins 1, ChREBP: carbohydrate response element binding proteins, FAHFA: fatty acyl esters of hydroxy fatty acid, FAO: fatty acid oxidation, LD: lipoid droplet

Figure 4 : Major adipocyte metabolic adaptations in white adipocytes in lean and high-fat fed conditions (normal BAT and beige homeostatic pathways are marked in black, with blue marked pathways and metabolites being specific to beige adipocytes (not found in BAT). HCHF- or HFD-fed altered pathways are marked in red, red arrows indicates pathways or metabolites reduced or increased in HCHF- or HFD-fed conditions)

Figure 4 Legend: In brown adipocytes lipids delivered via chylomicrons or VLDLs upon entry are utilization by oxidation with some FFA esterified into TAG and stored in lipid droplets which are small and multilocular. Glucose enters brown adipocytes via GLUT-4 (dependent on insulin, which also promotes DNL enzymes) participating in glycolysis with excess diverted to lipogenesis, contributing to lipid droplet expansion under tight regulatory controls. Glucose oxidations generate acetyl CoA in cytosolic mitochondria which enters TCA and ETC but expression of uncoupling protein-1 (UCP-1) (can be upregulated by diet induced sympathetic signaling) results in dissipation of heat and reduces ATP production, allowing greater energy expenditure. Beige adipocytes are

transformed from white adipocytes in nutrient excess or are synthesized from preadipocytes via branched-chain amino acid metabolites like 5OP, MOVA, BHIBA, BHIVA. Diet induced AMPK activation also promotes lipolysis and mitochondrial functionality to provide more fatty acids for FAO and resulting energy expenditure preventing LD expansion. Both brown and beige adipocytes contain another set of mitochondria associated with lipid droplet called peri-droplet mitochondria (PDM), which produce ATP and citrate for *de novo* lipogenesis necessary for BAT or beige maintenance. Beige adipocytes additionally employ a non-UCP-1 -dependent thermogenesis (via creatine phosphate). HCHF- or HFD-induced chylomicron delivers greater cargo increasing FFA concentrations. HFD or HCHF also impairs sympathetic signalling and AMPK activation which impairs WAT beiging. HFD or HCHF also impair both UCP-1- and non-UCP-1-dependent thermogenetic pathways reducing energy expenditure, which increases the shuttling of TAG (derived from chylomicrons) to be packages into lipid droplets to prevent lipotoxicity and increased LD size. Elevated SFAs, induced insulin resistance, ER and mitochondrial stress causing brown adipocyte death and BAT involution. Beige adipocyte energy expenditure, in initial calorie excess is protective, but in chronic cases, beige to white transition due to lipotoxicity further impairs energy homeostasis. DNL: de novo lipogenesis, SFA: saturated fatty acids, SIRT1: sirtuins 1, PDM: peri-droplet mitochondria,, FAO: fatty acid oxidation, SNS: sympathetic nervous system, UCP-1 : uncoupling protein-1, LD: lipoid droplet, 5OP: 5-oxoproline , MOVA: 3-methyl-2-oxovaleric acid , BHIBA: β-hydroxyisobutyric acid , BHIVA: β-hydroxyisovaleric acid

5.5 White adipose tissue maladaptation due to HCHF: Nutrient excess conditions as seen in HFD or HCHF prompt a greater storage of lipids in white adipose tissues due to their greater storage potential and relative ease of storage. In this regard, the metabolism in WAT undergoes drastic alterations in cases of HFD-or HCHF-consumption, which are of particular importance in understanding adiposity. In WAT, an initial high-calorie, high-carbohydrate diet increases *de novo* lipogenesis [286]. Increased intestinal derived chylomicron deliver more FFA to adipocytes facilitated by the upregulation of CD36 [312], which promotes greater fat entry into adipocytes. An increase in adipocyte FFAs

increase promotes TAG synthesis, which are redirected toward lipid droplets for storage to reduce lipotoxicity, increasing lipid droplet size. CIDE-A whose expression can attenuate HFD-induced obesity by limiting lipid droplets, may be downregulated by low circulating intestinal-derived LPS [292, 319]. In high-fat diet ingestion, the intestinal delivery of chylomicrons results in a large cache of intestinally synthesized ceramides, which may induce insulin dysfunction in adipose tissue [320]. An increased carbohydrate load stimulates *de novo* lipogenesis, increasing saturated fatty acid synthesis, during which intermediates are diverted to ceramide synthesis to prevent saturated fatty acid-induced toxicity [321]. Ceramide synthesis is upregulated by dietary fatty acids, and inflammation and downregulated by adiponectin and AMPK [321- 326]. Adiponectin and AMPK are reduced in metabolic syndrome, with concomitant metainflammation collectively increases ceramide biosynthesis and increases adipose concentrations of dihydroceramide (DHC), an intermediate of ceramide synthesis [327-329]. Elevated ceramide levels promote FFA uptake via PKC-ζ mediated upregulation of CD36 [322], further increasing lipid droplet size. Elevated ceramide levels affect membrane dynamics, impair mitochondrial functionality by initiating fragmentation, alter mitochondrial homeostasis via phosphocholine shuttling, and reduce mitochondrial respiratory and oxidative capacity [322, 330-332]. Mitochondrial dysfunction impairs insulin signaling [333], reducing insulin-mediated NO production. Ceramides can initiate ER stress directly or via the unfolded protein response (UPR) [334-336], which also severely impacts the normal functionality of adipocytes. Ceramide-induced mitochondrial alterations reduce mitochondrial functionality and increase ROS production, producing oxidative stress. Together, mitochondrial, oxidative and ER stress culminate in impeding insulin functionality [333]. Lipid-induced changes in mitochondrial functionality increase the production of reactive oxygen species, which, in turn, promote the formation of advanced glycation end products. High carbohydrate or low insulin activity increases glucose levels, which glycosylate extracellulat matrix (ECM) collagen proteins. Glycosylated ECM proteins and advanced glycation end products upregulate the expression of diaphanous-1-protein (Dia 1), a RhoGTPase regulator that affects RhoA, and ROCK expression and promotes insulin resistance

[333]. Metabolic overload and/or ceramides collectively induce adipocyte ER stress, leading to an unfolded protein response (UPR) that activates the JNK, IKK, and NF-κB pathways, further promoting insulin resistance [333]. The UPR also activates inflammasome-producing IL-1β and promotes the production of inflammatory cytokines such as TNF-α, inducing mitochondrial biogenesis impairment and insulin resistance [337]. Insulin dysfunction initiate a reduction in *de novo* lipogenesis, which reduces lipid signalling molecules like FAHFAs, further lowering systemic insulin sensitivity. Insulin and de novo lipogenesis dysfunction, both affect CIDE function, which coupled with increased lipid delivery increases adipocyte dimension mainly by lipid droplet expansion during nutrient overload [286, 338, 339]. During nutrient overload states, high glucose induces epigenomic changes promoting lipid accumulation [339]. CD36/FATP-mediated fat uptake also upregulates PPAR, which increases CIDE-A and G0 switch gene S2 (G_0S_2) [338]. Lipid droplet expansion is also facilitated by insulin dysfunction, since insulin can inhibit oleic acid-mediated CIDE-A expression and GOS2 protein expression (via the inhibition of AMPK and PPAR-α) [338]. In obesity or metabolic syndrome, lipolysis rates are not elevated (except only basal lipolysis), reducing TAG turnover rates [340], which further promotes increased TAG storage. The obesity-associated adipose lipidome shows elevated TAGs with polyunsaturated fatty acids (PUFAs), low saturated fatty acids (SFAs), or mono-unsaturated fatty acids (MUFAs) [316], with few unique lipid metabolites, such as specific 18:2 LPE species, that are elevated in obese people with insulin sensitivity [341]. Studies in metabolic syndrome patients have indicated depot-specific alterations in the lipidome with subcutaneous adipocytes demonstrating elevated long-chain PUFAs (20:4, 20:5, and 22:6), plasmanyl-phosphatidyl choline (PC) and visceral adipocytes showing increased plasmanyl-PE containing 18 carbon acyl chains [311, 318, 342, 343].

A chronic high-calorie, high-fat diet also enhances the lipogenic transcriptome, protein content, and cytoskeleton components, such as filamentous actin, septin, and vimentin in obese individuals, increasing adipocyte size [344-346]. Lipid droplet enlargement reorganizes the adipocyte plasma membrane, where the close approximation of lipid droplets with plasma membrane reduces the

clustered caveolae space, increases pressure and facilitates expansion [347]. Adipocyte lipid expansion creates a mechanical stretch response that induces flattening of caveolae, activating ion channels, upregulation of caveolae-synthesizing proteins, and increased caveolae migration from the cell membrane to the cytosol [347-350]. Upregulation of caveolae-mediated transport increases FFA delivery, promoting size expansion. The flattening of caveolae triggers restructuring of anion channels that destabilizes insulin-PI3K-AKT2 signaling [349, 351, 352]. The lipid droplet-associated protein perilipin 1 (PLIN1) regulates the accessibility of lipases to lipid droplets and is inhibited in individuals with diet-induced obesity, shifting the balance towards lipid droplet expansion [353].

Larger-sized lipid droplets are associated with unhealthy expansion of adipose tissues characterized by altered transcriptome signature [354, 355] in contrast to healthy expansion, which involves multiple smaller-sized lipid droplets [356]. In response to a high-calorie, high-fat diet, white adipose tissue responds by increasing lipid storage capabilities, mainly by either increasing their size (hypertrophy) or the number of adipocytes (hyperplasia). Hyperplasia acts as a protective mechanism to prevent metabolic dysfunction promoting elusive healthy obesity. Hyperplasia involves adipogenesis, a protective mechanism preventing hypertrophy, thus inducing adipogenesis and preventing hypertrophy are key mediators in preventing nutrient excess obesity.

Lipid deposition under nutrient-excess conditions occurs predominantly in white adipose tissue, which is divided into two categories based on anatomical locations: visceral adipose tissue (VAT) and subcutaneous adipose tissue (SAT). Human SAT contains cells with a single fat droplet, whereas perivascular fat pads have adipocytes with multiple smaller droplets [357]. Studies indicate that nutrient excess conditions cause initial fat deposition in SAT cells. The initial fat storage in the SAT causes adipocyte hypertrophy, which is associated with metabolic dysregulation [356, 358]. The adipogenic capacity of SAT is reduced due to insulin dysfunction, and high glucose/FFA-induced ROS, which further promotes SAT hypertrophy as means of accommodating excess lipids [359, 360]. The inhibition of adipogenesis may occur due to inhibition of *de novo* lipogenesis (due to insulin dysfunction) or via high-fat diet-induced epigenetic reduction of H3K23 acetylation [124, 286, 290].

Current studies also predict that high-calorie and high-fat induced obesity promotes senescence on SAT preadipocytes, thus limiting adipogenesis and favouring hypertrophy [361]. The reduced adipogenic potential of SAT are currently being challenged as recent evidence has indicated that SAT adipocytes in obese-prone individuals and their first-degree male relative actually show a different TAG-fatty acid composition with more TAG associated with PUFA which promotes inflammation and reduced insulin signaling [362], which may affect adipogenesis.

The continued expansion of SAT adipocytes requires constant ECM modifications to accommodate increasing size which requires active collagen degradation [363]. The degradation of collagen requires the active participation of macrophages but in hypertrophied adipocytes, macrophage dysfunctions impairs collagen degradation [363]. Undegraded collagen accumulates as short fragments have biological actions, stimulating macrophage proliferation and causing fibroinflammatory changes that limits SAT hypertrophy [363]. The continued storage of TAGs in SATs ultimately reaches the maximum storage capacity [364-366], which mediates the spillover phenomenon, leading to an increasing diversion of TAGs toward VATs for storage.

Recent studies have identified a population of adipose-specific mitochondria associated with lipid droplets called PeriDroplet mitochondria (PDM). These PDM mitochondria's in beige and brown adipocytes synthesize ATP, which negatively correlates with an increase in lipid droplet size, linking lipid droplet enlargement and metabolic dysregulation and autophagy upregulation [367, 368], but research in PDM functions in white adipocytes is still unclear.

Visceral adipocytes undergo constant adipocyte enlargement due to high TAG flux for storage and ECM modification [369, 370]. ECM modifications include increased levels of collagen VI, and fibronectin [369, 371, 372]. Hypertrophic visceral adipocytes have increased matrix metalloproteinase (MMP) activity, which helps in ECM degradation, creating space for expanding adipocytes [373]. Collagen VI cross-linking in ECM, limits adipocyte expansion due to tissue stiffness, thus limiting VAT storage [369]. The hypertrophy of adipocytes causes vascular rarefaction and hypoxia, which might further divert glucose toward lactate production. Hypertrophied adipocytes develop into

insulin-resistant adipocytes with lower vascular density, and a high-fat diet in murine models has been shown to reduce the number of preadipocytes and transcriptionally active adipocytes [374]. This reduction in precursor cell population reduces hyperplasia, a protective mechanism since new adipocytes retain insulin sensitivity [364, 375]. The ability of adipocyte expansion to alter vascular density [376, 377] is well proven in rodent models of obesity, but in human studies, a reduction in oxygen tension is attributed to vascular density rarefication [378, 379]. Hypoxia induces mitochondrial dysfunction, alter the degree of crosstalk between adipocytes and surrounding cells [379, 380]. Hypoxia also upregulates hypoxia-inducible factor-1 alpha (HIF-1α), reducing branched-chain amino acid (BCAA) catabolism which increases plasma BCAAs and PAI-1 levels, which further reduce systemic insulin sensitivity [379, 381]. Hypoxia-induced HIF-1α expression decreases adipocyte maturation [382], enhances profibrotic gene expression, and ECM collagen cross-linking promoting fibrosis. Fibroinflammatory changes increase the production of AGEs, which promotes macrophage infiltration, which in a high-AGE milieu becomes polarized towards the M1 profile [373]. Hypoxia-induced ECM fibronectin expansion also plays a role in limiting adipocyte expansion [373]. Hypoxia via RhoA/ROCK reduces adipogenesis in the initial stages of obesity thus favouring hypertrophy [373, 383, 384]. Increased FFA drive adipocytic DHC synthesis, which suppress VAT expansion, and with collagen deposition and fibrosis limiting adipocyte expansion, FFAs escape into systemic circulation for ectopic lipid deposition [385].

Many studies also indicate elevated adipogenesis in obesity and metabolic syndrome [356]. Adipogenesis impacts obesity or metabolic syndrome since the dysregulated expansion of small adipocytes is associated with decreased insulin sensitivity [387]. Hypertrophic adipocytes are inflammatory in nature, and produce inflammatory cytokines [388], which activate autophagy [389]. In adipose tissue, autophagy plays a vital role in maintaining adipose tissue homeostasis, with excess autophagy being associated with lipodystrophy and glucose intolerance [390]. The process of autophagy requires an abundant supply of phospholipids derived from *de novo* lipogenesis, which induces initial reductions in autophagy inhibiting adipogenesis inducing hypertrophy [391].

Hypertrophic adipocyte accumulated saturated fatty acids induce ER stress response, which coupled with adiponiche ECM remodeling, tissue stiffness, and inflammatory cytokines can upregulate the expression of autophagic genes, promoting adipogenesis [389, 392, 393]. Despite the current findings, the causative factor for increased adipogenesis in metabolic syndrome has not been universally accepted. However, lineage tracing studies have shown that the occurrence of adipogenesis in preadipocytes located in peri-vasculature contributes to adipose tissue expansion in nutrient overload conditions [394]. One of the major factors responsible for autophagy upregulation is the local inflammatory response in adipose tissue [395]. Autophagy impairs PLIN1 in adipocytes, allowing lipolysis enzymes to hydrolyze lipid droplet TAGs from FFAs, which are then released into the systemic circulation, promoting ectopic lipid accumulation [396]. Hypertrophy-induced hypoxia promotes angiogenesis, facilitating increased nutrient supply to cells and inducing HIF-1α activation, which plays a role in ECM modification [397]. ECM modification requires MMPs (MMP-14 namely) which are upregulated in the initial stages of obesity to promote healthy expansion in obesity [398]. Chronic high MMP-14 levels digest collagen VI to produce endotrophin, which stimulates fibrosis and inflammation [398].

Inflammation in white adipose tissue in HCHD fed conditions: Initial insulin resistance-induced upregulation of insulin secretion by pancreatic beta-cells causes hyperinsulinemia and promotes senescence in adipocytes, endothelial cells, T-cell, and macrophages in VATs. Senescence increases cytokine release as part of a senescence-associated secretory phenotype (SASP) [399], regulating apoptosis in senescent cells. High levels of saturated FFAs in hypertrophied adipocytes activate JNK pathway in macrophages, stimulating inflammatory signalling [400]. High systemic LPS (intestine-derived) binds to TLR4 receptors and induces the expression of inflammatory cytokine genes in macrophages. Hypoxia-induced mitochondrial dysfunction promotes increased interstitial lactate levels in obese adipose tissue, which increases insulin resistance [401]. Lactate production in adipocytes is also elevated to help maintain redox balance [402], which then stabilizes HIF-1α and promotes M1 macrophage. M1 macrophage polarization increases IL-1β production, which increases

the number of TLR4 ligands in M1-polarized macrophages and rewires the CD4+ T-cell metabolism [403, 404]. Elevated lactate levels also promote white adipogenesis, further increasing the adipogenic proliferation of cells [402]. Elevated metabolic load in obese white adipocytes, local hypoxia, large TAG stores, and elevated ceramides cause ER stress, which induces adipocyte death via apoptosis (cathepsin or Bax-mediated), mitophagy, or pyroptosis mediated by elevated ROS production [405–407]. Adipocyte death recruit's immune cells (monocytes and macrophages) influencing inflammation. Together, adipocyte death, inflammation, autophagy and elevated lactate may induce adipogenesis, which produces new adipocytes as a means of overcoming the negative of hypertrophic expansion. Since newly formed adipocytes are smaller and have better insulin sensitivity, they take up more glucose and act as additional storage sites, causing their hypertrophy and thus initiating a viscous cycle.

With the incorporation of fructose into the diet, the hepatic metabolism of fructose generates advanced glycation end products (AGEs), such as carboxymethyllysine (CML) [408]. Elevated ROS might induce the activation of the NLRP3 inflammasome, which further increases the production of proinflammatory cytokines [409]. AGEs interact with their cognate receptors for AGEs (RAGE) in various cells, such as adipocytes and macrophages, dysregulate adipokine release, and increase ROS generation (via the AGE-RAGE-Ras-MAPK-NADPH oxidase pathway). AGE-RAGE interactions also enhance proinflammatory cytokine production via NF-κB activation, initiating inflammation [410] and upregulating protein tyrosine phosphatase 1B (PTP1B) and suppressor of cytokine signaling 3 (SOCS3), which inhibit insulin signaling [333, 335]. In metabolic syndrome or obesity-associated inflammation, elevated proinflammatory cytokines such as TNF-α, ROS, and insulin resistance lowers endothelial nitric oxide production, leukocyte adhesion, and platelet aggregation, further increasing inflammatory response, which is ameliorated by dietary nitrate or arginine administration in rodent HFD models [411, 412]. Elevated proinflammatory cytokines in MetS-induced inflammation inhibit the release of iron from macrophages, promoting iron overload in

macrophages in adipose tissue, and leading to the initiation of ferroptosis which changes the macrophage polarization to the M1 phenotype and initiate cell death [413-415].

5.6 Beige and brown adipose tissue maladaptation due to HCHF diet consumption:

The brown adipose tissue are important cells involved in energy expenditure via thermogenesis. So understanding how HCHF or HFD induces thermogenesis impairment may be critical in understanding how HCHF or HFD translates into energy excess states that impair functionality of other tissue like liver, WATs. WAT conversion to beige adipocytes in cases of nutrient excess or beiging stimuluses, also are important in-built protective mechanisms in nutrient excess conditions, an impairment of which might play a role in HCHF- or HFD-induced metabolic alterations.

5.6.1 Beige adipocytes maladaptation: In the initial nutrient overload state, white-to-beige transition is used as a means of improving metabolic health via PPAR expression, which can affect mitochondrial biogenesis [304]. Prostacyclin produced by the endothelium is a potent vasodilator that induces adipogenesis and WAT adipocyte beiging [416]. Exposure of beiging inducing signal, upregulate cyclic-AMP (cAMP) concentrations in WATs [417], which then promotes BCAA metabolism producing metabolites, such as 5-oxoproline (5OP), 3-methyl-2-oxovaleric acid (MOVA), β-hydroxyisobutyric acid (BHIBA), and β-hydroxyisovaleric acid (BHIVA) [418]. These metabolites induce a WAT beiging phenotype with expression of thermogenic genes in naïve or WAT adipocytes [418]. MOVA, 5OP, BHIBA and BHIVA also promote skeletal muscle mitochondrial metabolism influencing energy expenditure [418]. Elevated cAMP also promotes lipolysis, generating FFAs, which undergo beta-oxidation. Beta-oxidation generates acetyl CoA which upregulates UCP-1 expression, promotes thermogenesis, and induces the production of heat and less ATP favoring energy expenditure [417]. Elevated AMPK levels maintain mitophagy and autophagy which improves mitochondrial quality to maintain thermogenic program. Increased glucose entry allows more *de novo* lipogenesis, which supports autophagy and UCP-1 expressions [286]. Increased chylomicron delivery of TAGs and their subsequent breakdown by LPL produces FFAs. FFAs enter beige adipocytes via CD36 and FATP, which along with *de novo* lipogenesis derived FFA are then utilized for TAG

synthesis and packaged into lipid droplets [417, 418]. HCHF diet consumption thus increases lipid droplet size which then creates hypoxia which reduces BCAA metabolism and its metabolites [379, 381]. HFD also independently impairs sympathetic signaling which collectively reduces cAMP concentrations and lipolysis and downregulates UCP-1 expression [417]. HFD induced elevated saturated fatty acids (palmitate) independently can also decrease UCP-1 and AMPK activity, both of which affect thermogenic capacity [417, 418]. Beige adipocytes also employ a UCP-1-independent thermogenesis programme involving the phosphocreatine system, which involves creatine that promotes mitochondrial ATP turnover and induces macrophage M2-polarization [419]. In obesity, phosphocreatine metabolism is altered which changes ATP/ADP ratio inhibiting AMPK [419]. AMPK inactivation impairs mitochondrial functionality and increases the production of proinflammatory cytokine [419]. Chronic HCHF or HFD-diet induces insulin resistance, which reduces de novo lipogenesis, and impairs beige adipogenesis. A diet rich in fats or saturated fats upregulates mitophagy, which when elevated induces whitening in metabolic syndrome, promoting adverse metabolic conditions characterized by increased body fat and insulin resistance [420].

Both beige and brown adipocytes also have peridroplet mitochondria's (PDM) which are associated with lipid droplets. The PDM generates ATP for energy purposes by utilizing glucose and generates cytosolic citrate for *de novo* lipogenesis [417, 421]. PDM-generated citrate and de novo lipogenesis also upregulates CIDE-A expression which allows more lipid accumulation into lipid droplets to prevent lipotoxicity [417, 421]. In obesity, activity of PDM is upregulated leading to increased lipid droplet size which may play a role in alteration of thermogenic potential of beige and brown adipocytes [421, 422].

5.6.2 Brown adipocyte maladaptation: HCHF consumption increases diet-induced intestinal signal molecules like cholecystokinin, secretin, GLP-1, and bile acids, which stimulate central sympathetic nervous system, stimulating cAMP-dependent lipolysis [417]. Lipolysis generates fatty acids for oxidation with thermogenesis dissipating energy as heat [417]. Initial glucose excess, allows more glucose entry into BATs via GLUT-4, which undergoes glycolysis to generate pyruvate, which forms

acetyl CoA in PDM (upregulated), which is exported to cytosol via citrate. Cytosolic citrate breakdown generates acetyl CoA which promotes UCP-1 activity [417]. Glucose intake in BATs also stimulates ChREBP, which mediated *denovo* lipogenesis, which remodels the mitochondria and facilitates thermogenesis. In cases of initial HCHF or HFD consumption, initial stages the rates of thermogenesis and *de novo* lipogenesis are increased increasing the lipid droplet size. But chronic HCHF diet consumption causes insulin resistance which prevents glucose entry and reduces *de novo* lipogenesis. High-fat diet also reduces the sympathetic neuronal receptor sensitivity thus reducing cAMP-PKA stimulation [417]. Increased chylomicrons deliver FFAs to BATs which causes a buildup of saturated fats which inhibits AMPK and UCP-1 [417], followed by conversion of fatty acids to ceramides to prevent lipotoxicity. Ceramides that are positively correlated with obesity and metabolic syndrome when elevated interferes with mitochondrial oxidation and impairs thermogenesis. In addition to this, the adipose tissue turnover rates are increased, which increases the intracellular purine levels, which inhibits UCP-1 activity [424], thus effectively reducing BAT thermogenesis. Reduction in UCP-1 expressions, causing BAT involution [420]. BAT populations normally decline with age, remaining around deeper organs, but in pathological conditions like obesity, there is a drastic reduction in BAT quantity. HCHF-diet-induced ROS damages mitochondrial architecture and preventing the lysosomal clearance of damaged mitochondrial components, impairing thermogenetic efficiency and energy metabolism [425-428]. BAT-resident macrophages maintain normal thermogenesis [425, 428, 429] by degrading oxidized components [430, 431] via CD36 receptors [425], which when fed a high-fat-diet, becomes defective probably due to alternative M1 polarization, suppresses UCP-1 expression [428]. Defective delivery of mitochondrial components to macrophages [428, 430] drives the accumulation of endocytic vesicles containing damaged components, downregulating UCP1 expression [425, 428]. When thermogenesis is inhibited, endothelial cell-derived stem cell factor (SCF) upregulates lipogenic enzymes in BATs, further increasing lipid accumulation and lipid droplet expansion. Constant lipid droplet expansion causes vascular rarefication-induced hypoxia, which inhibits β-adrenergic receptor stimulation [432],

limiting thermogenesis that may promote BAT whitening or brown adipocyte death. Excessive lipid droplet accumulation in BAT induces hypoxia which promotes macrophage infiltration, inflammation [433-437], and downregulate essential genes like PPARγ, SIRT1, and CPT1, IRS-2, and GLUT-4 collectively induce BAT whitening and death [435, 438]. BAT involutions seen in humans during aging (not established in rodents) are attributed to sympathetic impairment, dysregulated hormonal function, adipocyte inflammation, lower UCP1-induced thermogenic capacity, senescence [420, 439]. Chronic high-fat diet feeding induced a decrease in syntaxin-4 expression in BAT, lowering glucose uptake, glycolytic energy acquisition, and mitochondrial respiratory capacity via UCP1 destabilization, thus lowering thermogenic capacity [440]. Reduced ATP production induces pyroptotic death in BAT, causing premature BAT involution [440]. Chronic high-fat carbohydrate consumption reduces UCP1 expression, inhibits VEGF-A functionality, impairs angiogenesis [441]. High-fat induced mitophagy dysregulation [442], mitochondrial fragmentation [443], low mitochondrial DNA copy numbers [444] may also account for limited thermogenic expenditure. The loss of thermogenic capacity of brown adipocytes, severely impacts the host energy expenditure, further promoting the excessive nutrient storage into non-adipose tissues such as liver, cardiac tissue and skeletal muscles.

Adipose tissue maladaptation in HCHF- or HFD-fed scenarios thus involves a concerted dysfunctional hypertrophy and hyperplasia of white adipocytes, coupled with reductions in brown and beige adipocyte populations. Current evidence in rodents indicates that white adipocytes exhibit different subpopulations, one being lipogenic adipocytes (LGAs) which are the smallest cells expressing elevated levels of *de novo* lipogenesis genes, and the second set of cells being lipid scavengers (LSAs) expressing high levels of CD36-like genes for lipid uptake [445]. The last subset of cells is the stressed lipid-scavenger (SLSA) subset, the largest in size among the three types of cells, which express hypoxia- and autophagy-related genes [445]. A high-fat diet increases the relative populations of LSA and SLSA, while reducing LGA, which may explain the reduced *de*

novo lipogenesis in HFD [445]. This observation still needs to be validated in human tissue samples and temporal factors that mediate the switch between cell-types still need to be identified, so that newer therapeutic approaches can be designed to limit white adipocyte expansion.

Chronic HFD or HCHF consumption causes maladaptation, coupled with gut-derived LPS and immune cells intensely stimulate the adipose tissue immune system. The adipose tissue immune system, which is involved in regulating inflammation also needs to be disrupted in cases of HFD or HCHF consumption, to potentiate inflammation.

6 Adipose Tissue Immunosurveillance– Key Players

Adipose tissue immune system is a robust system that response to metabolic load, systemic inflammation and helps to maintain adipose tissue homeostasis. The various types of immune cells in adipocytes include T-lymphocytes (CD4+ and CD8+), B-lymphocytes, macrophages, iNKT cells, and ILCs. These immune cells have specific functions in lean adipose tissue, which are altered in HCHF- or HFD-induced obesity or metabolic syndrome. The major reasons for immune cell function impairment may be due to alterations in cytokine profiles (induced by HFD or HCHF) or gut-derived LPS, all of which affect the metabolism in these immune cells and alter their expected immune functionalities. Inflammatory mediatory and cytokine can alter the metabolic pathways immune cells and impede their functions, so a knowledge of such changes might help in shedding light on specific pathways which can be targeted to reduce adipose tissue inflammation.

6.1 Adipose macrophages and their metabolic alteration in obesity/metabolic syndrome:

Macrophages are important adipose tissue-resident immune cells that are important components of immunity [446]. Under lean conditions, eosinophils, lean adipocytes release IL-4, which induces macrophage polarization toward the M2 anti-inflammatory phenotype [447]. M2-polarized macrophages utilize glucose through the Akt and mTOR pathways with further metabolism via the TCA cycle and OXPHOS to generate energy. FAO supplies additional energy by utilizing fatty acid (derived from fatty acid synthase via FATP1 or TAG lipolysis) oxidation metabolized via OXPHOS for maintenance of the M2 profile [447]. In M2 polarized macrophages, arginine catabolism generates

ornithine, urea, and polyamines to inhibit the expression of inflammatory genes [447]. Nutrient overload with a high-fat and high-calorie diet increases serum LPS and IFN-gamma, which promote macrophage M1 polarization and with increasing obesity, the number of M2 macrophages decrease, with an associated increase in monocyte-derived proinflammatory M1 macrophages [448]. M1-polarized macrophages utilize glucose via glycolysis and the TCA cycle [447, 448]. M1-macrophages utilize arginine to produce NO, which inhibits the OXPHOS system, allowing greater dependence on glycolysis for energy [447]. OXPHOS inhibition causes the accumulation of citrate, which is converted to itaconate [447]. Itaconate and NO inhibit ETC/OXPHOS, supporting M1 polarization [447]. Under normal conditions, macrophages are protected from NO toxicity [449], but it is overwhelmed in individuals with metabolic syndrome or obesity, where elevated nitric oxide can damage the ETC in macrophages. A reduction in the oxidative capacity of the ETC allows glucose entry into the HMP shunt pathway, resulting in the production of NADPH and ribose-5-phosphate [449]. NADPH is utilized to generate more ROS via NADPH oxidase, and LPS-exposure increases itaconate accumulation, which via succinate stimulates ROS production [450]. LPS-exposure also inactivates AMPK, which initiates reverse electron transport (RET), driving ROS production, activating HIF1α, and inducing NLRP3 activation facilitating IL-1β production [451, 452]. Elevated ROS, LPS-induced mTOR, and IL-1β drive inflammation by stabilizing hypoxia-induced HIF1α [452-454]. Macrophages stimulated by LPS, TNF-α or IFN-γ, demonstrates the upregulation of glycolysis and inhibition of OXPHOS, both of which are very important for its proinflammatory activity. The inhibition of OXPHOS allows mitochondrial accumulation of citrate, which is transported to cytosol via mitochondrial citrate transporter [455, 456]. Citrate is processed in the cytosol to generate NO and ROS, both of which promote the activation of macrophages, DCs, and NK cells [447, 455]. Cytosolic citrate, can enter *de novo* lipogenesis and produce arachidonic acid, required for prostaglandin E2 (PGE2) synthesis. Arachidonic acid also mediates LPS-induced IL-1β synthesis [457]. Upregulation of glycolytic enzymes such as pyruvate kinase M2 (PKM2) and hexokinase-1 induces inflammasome activation and IL-1β production [458]. Another enzyme

pyruvate dehydrogenase (PDH) fuels itaconate production from citrate, and glyceraldehyde-3-phosphate dehydrogenase (GAPDH) regulates TNF secretion [458]. Succinate accumulation generates a feedforward loop, informing macrophages and other cells in the local microenvironment about inflammation via IL-1β production [458]. Excess nutrients in combinations with LPS induce *de novo* lipogenesis to produce palmitate, promoting NLRP3 inflammasome activation and IL-1β and IL-18 production. LPS-induced inflammatory cytokines such as IL-1β and TNF reduce insulin sensitivity by downregulating insulin signaling pathways/proteins [333]. In metabolic syndrome, multiple mechanisms, such as FFA-mediated PPAR-gamma signaling, LPS- and/or IFN-gamma-induced expression of TNF, downregulation of proinflammatory mediators, and ER stress in adipocytes, play a role in macrophage polarization [458]. A high-fat diet and associated inflammatory cytokines induce metabolic reprogramming, in macrophages, resulting in an inflammatory phenotype that induces low-grade chronic inflammation.

6.2 Adipose tissue invariant natural killer T-cells: In lean adipose tissue, adipose tissue invariant natural killer T (iNKT) cells produce IL-10 along with IL-2 to support anti-inflammatory T_{reg} cell proliferation and macrophage homeostasis. iNKT cell functionality is regulated by AMPK; however, in HFD- or HCHF-induced obesity, AMPK inactivity/inhibition impairs iNKT physiology [459]. Diet-induced besity reduces the numbers of iNKT cells and remaining cells exhibit increasing pathogenicity due to excess lipid exposure [460]. Adipose-resident iNKT cells play anti-inflammatory and inflammatory roles that are dependent on different subpopulations of iNKT cells. Three subpopulations of iNKT cells have been identified in rodent adipose tissue, one subtype consist of As-iNKT1 cells that produce IFNγ and TNFα. The second subtype is A-iNKT17 cells, that secrete IL-17A, and IL-4, and the third subtype consists of Au-iNKT1 cells with an intermediate Th1- and Th2-cytokine profile [461, 462]. The response to any nutrient or metabolic stimulus, such as high-fat diet, depends on the stimulation of specific cell subtype ratios [461]. Under homeostatic conditions, enlarged adipocytes (if any) express lipid markers (such as ceramide and ceramide metabolites), which via ceramide-antigen presenting cell (APC) interactions activate iNKT cells [461]. Activated

iNKT cells remove oversized adipocytes and stimulates the differentiation of healthy adipocytes by enhancing the proliferation of adipocyte progenitors, also encouraging the synthesis of FGF21 and thermogenesis in adipocytes [463-466]. iNKT cell–macrophage interactions influence the overall inflammatory status and modulate glucose homeostasis via insulin sensitivity/resistance [467]. Under homeostatic conditions, iNKT cells induce M2 polarization via IL-10 [434], but HFD challenge for 4 days (acute) or 8 weeks (prolonged) induces iNKT-macrophage M2 polarization via IL-4 & α-GalCer [467]. Prolonged HFD challenge for 16 weeks causes iNKT-induced M1 proinflammatory macrophage polarization, via α-GalCer [467]. Studies have also revealed that a short-term HFD triggers increased infiltration of iNKT cells in the adipose tissue, which is reversed in long-term HFD feeding [467] as observed in obese adipose tissue, where the number of iNKT cells decreases, inversely correlating with proinflammatory macrophages infiltration [467].

6.3 Adipose dendritic cells: Conventional dendritic cells (cDCs) modulate tissue metabolism and immune homeostasis via adipocyte beta–catenin–mediated IL-10 secretion, recruiting Treg cells and limiting proinflammatory signaling cascades [468]. cDCs promote adipocyte lipolysis and scavenge free lipids, which influence antigen cross-presentation capabilities [468], promoting preadipocyte differentiation and adipose tissue expansion [469]. In obese individuals, dysregulation of the adipokine chemerin results in the recruitment of circulating plasmacytoid dendritic cells (pDCs) into VAT, which accumulate with obesity [470]. The accumulation of adipose-tissue dendritic cells in subcutaneous adipose tissue impairs the expansion of healthy adipose tissue, and glucose sensitivity [471].

6.4 Adipose resident T-cells: Adipose-resident T-lymphocytes are classified into two broad subtypes, namely CD4+ helper T cells (Th cells) and CD8+ cytotoxic T- cells. The T-helper cell subtypes are Th1, Th2, Th17, and T_{reg} cells. Antigenic stimulation promotes the CD8+-T response and induces CD4+ T cells to differentiate into four subsets, including Th1, Th2, Th17, and inducible regulatory T (i-Treg) cells depending on the cytokine milieu [472].

Cytotoxic CD8+ T-cells secrete IFN-γ, which regulates inflammation by killing stressed or dysfunctional adipocytes, contributing to the remodeling of adipose tissue [472]. In metabolic syndrome, cytotoxic T-cells infiltrate adipose tissue, causing increased production of IFN-γ. IFN-γ recruits multiple immune cells, such as macrophages, exacerbating inflammation and insulin resistance [473].

6.4.1 Adipose T-helper cells: Among T-helper cells, adipose resident T_{reg} cells are major regulators of adipose homeostasis. T_{reg} cells include iT_{reg} and nT_{reg}, which inhibit the inflammatory response by secreting IL-10 and TGF-β [474]. In lean states, adipocyte-secreted IL-33 maintains regulatory T (T_{reg}) (CD4+FOXP3+), which induces *Ucp1* expression, promoting thermogenesis to induce browning. T_{reg} cells produce IL-4 and IL-13 to stimulate adipocyte browning, support anti-inflammatory macrophage populations [475], and promote insulin sensitivity [476]. In lean adipose tissues, naïve T_{reg} cells exhibit low dependence on glycolysis, with FAO and OXPHOS providing energy [472, 477]. In lean adipose tissues, upon activation, dependence on glycolysis increases (promoted by mTOR) with FAO (supported by PPAR-γ expression) still being used for energy [472, 477]. In lean adipose tissues, T_{reg} activation increases the conversion of tryptophan to kynurenine, reducing tryptophan bioavailability [478] thus increasing FOXP3 expression to produce suppressive cytokines [477]. Suppressive cytokines such as IL-10, TGF-β, and adenosine (ADO) suppress Th17 differentiation, and regulate Th1 and Th17 responses [479, 480]. An increase in adipose T_{reg} cells plays a role in attenuating adipose inflammation and promoting insulin sensitivity [481].

In obese visceral adipose tissues, plasmacytoid dendritic cells accumulate due to high-fat feeding, producing IFN-α and downregulating the expression of PPAR-γ, which decreases T_{reg} cell numbers [482]. In diseases such as hypercholesterolemia, elevated levels of isobutyric acid and isovaleric acid may regulate T_{reg} activity via mTOR hyperactivity [483-487]. T_{reg} cells in brown and beige adipocytes increases thermogenesis, and T_{reg} cell reduction might affect the thermogenic capacity of brown/beige adipocytes [488].

In individuals with obesity, the concentration of Th1 cells in adipose tissue increases, mainly due to a reduction in T_{reg} cells [489]. In obesity, Th1 cells exhibit an altered metabolic profile characterized by increased glycolytic dependence on energy production. High-fat diet-induced mitochondrial dysfunction in Th1 cells promotes ROS production, altering FAO oxidation, which collectively promotes the production of proinflammatory cytokines such as IFN-γ, IL-1β, and IL-6. These proinflammatory cytokines induce Th17 cell differentiation, resulting in the production of copious amounts of IL-17 [490]. IL-17 has been implicated in the secretion of proinflammatory cytokines, cell death, and autophagy.

Th2 cells are anti-inflammatory and utilize glycolysis and fatty acid oxidation with OXPHOS for energy generation [491]. These cells produce cytokines such as IL-4, -5, and -13 that promote M2 macrophage polarization, interact with T_{reg} cells, and promote proliferation and differentiation of B cells [491]. Th2-produced cytokines regulate eosinophil functions to maintain an anti-inflammatory environment and produce IL-4, which reduces adipocytic lipid accumulation [491]. In obesity or associated metabolic syndrome, the number of Th2 cells in adipose tissue gradually decreases, reducing anti-inflammatory immune cell functions [492].

IL-17 secreting Th17 cells, which are proinflammatory, are generally inhibited in adipose tissue by T_{reg} cells. In cases of obesity-associated metabolic syndrome, a reduction in gut-derived SCFAs, and T_{reg} cells, the migration of gut-derived Th17 to adipose tissue, and an altered adipokine profile induce the proliferation and differentiation of Th17 cells [493]. Differentiated Th17 cells exhibit increased glycolytic dependence on energy, and fatty acid synthesis supporting the production of the inflammatory cytokine Il-17, 21 and 22, which exacerbate insulin resistance and inflammation [493]. WAT-resident T-cells can differentiate into memory cells with obesity-promoting memory T cells, which, upon rechallenge (chronic nutrient excess), respond to infection and promote fat cell necrosis and death [494].

6.5 Adipose B-lymphocytes: B-lymphocytes, important mediators of the immune response, are synthesized in the bone marrow. Immature B cell exit the marrow and travel toward the periphery or

spleen for maturation, where they transform into naïve B cell, naïve activated B cell, germinal center (GC) B cell, and mature memory or plasma B cell. Undifferentiated B cell utilize glucose via glycolysis, the TCA cycle, and OXPHOS for energy production, which is influenced by mTOR and c-Myc. Plasma cell differentiation involves greater glucose utilization and amino acid uptake for protein (antibody) generation, the glycosylation of antibodies, and survival [495]. In obesity, B-cell size is positively correlated with BMI [496], with no observable change in B-cell numbers. In adipose tissue in obesity or metabolic syndrome, T-bet expression in some B cells is induced, resulting in Tbet+ B cell expressing CD11c+. Moreover, some cells retain Tbet- CD11c- B cell characteristics [496]. Tbet+ B cell expansion that occurs in obesity, induces iNKT cell transition to IFN-γ-producing cells [497] exacerbating immune dysregulation. Obesity or insulin resistance in metabolic syndrome is associated with B-lymphocyte dysfunction, with a positive association between B-cells and high-fat-induced changes in adipose tissues [496]. During high-fat diet challenge, epididymal VAT shows the earliest accumulation of B cell, which produce inflammatory cytokines such as IFN-γ and IL-6, TNF-α, and MCP-1 [499]. Current research indicates that two subtypes of B-cells namely B1 and B2 are involved in adipose tissues homeostasis. B1 cells are anti-inflammatory, produce IgM and IL-10, and are reduced in diabetic or obese patients due to dead adipocyte-IgG mediated mechanisms [500, 502]. In comparison, B2 cells are proinflammatory and secrete IgG molecules (IgG2c) and cytokines (TNF, IFNγ, MCP1, IL-6, and IL-8), driving tissue immunometabolic dysfunction [501, 502]. B2 cells are elevated in obesity-associated inflammation with reduced B regulatory cells [501, 502]. Adipose tissue B regulatory cells (B_{reg} cells) are another subset of B cell that help maintain immune homeostasis by producing antibodies and IL-10 (an anti-inflammatory cytokine) [502]. Fasting-induced lipolysis releases fatty acids such as palmitate, which activates TLR4 in B_{reg} cells, inducing IL-10 production and promoting B cell viability [502]. B_{reg} cells inhibit macrophage or dendritic cell antigen presentation, Th1 and Th2 responses, and proinflammatory cytokine production via IL-10 [502]. In individuals with obesity or metabolic syndrome, B_{reg} cells tend to reduce the number of IL-

10 secreting cells, increase autoantibody production, and increase the number of TNF-α and IL-6-producing cells [502].

6.6 Adipose innate lymphoid cells: Innate lymphoid cells are tissue-resident immune cells that play a role in immunity, inflammation, and resolution. There are three types of innate lymphoid cells: ILC1s (T-bet+ group 1 ILCs), ILC2s (GATA3+ group 2 ILCs), and ILC3s (RAR-related orphan receptor- γt+ (RORγt+) group 3 ILCs) [503].

6.6.1 ILC1 cells: ILC1s include cytotoxic natural killer (NK) cells, which are stimulated by proinflammatory cytokines IL-12 and IL-18 to produce IFN-γ and TNF [503, 504]. ILC1s utilize mTOR-dependent glycolytic and OXPHOS systems for energy acquisition [404]. The glycolytic end product pyruvate is converted to lactate (in small amounts) [505], with small quantities of glucose-derived acetyl CoA diverted to the cytosol as citrate, remaining citrate fuels OXPHOS, ATP synthesis [506-508], and cytosolic citrate funneling into fatty acid synthesis. Amino acid glutamine metabolism feeds the TCA cycle, supporting c-Myc [509] and regulating the expression of glucose transporters and glycolytic enzymes [509]. In lean adipose tissue, NK cells kill M2-polarized macrophages after their homeostatic role is complete, thus preventing their transformation into proinflammatory M1 phenotype and maintaining adipose tissue homeostasis [510]. In obese adipose tissue, the number of NK cells increase at the onset of nutrient excess, indicating its role as a stress marker [510]. Large adipocytes, low adiponectin and hypoxia (via HIF-1α) promote NK cell entry into adipose tissue [510]. HFD-challenged adipose NK cells exhibit faster expansion and elevated IFN-γ and TNF production [511]. This hyperactivity may be due to increased expression of NK cell-activating receptor ligands in adipocytes in obesity [510]. Obesity-associated aberrant PPAR expressions, promotes increased NK cell lipid uptake and accumulation, which inhibits mTOR pathway, c-Myc expression inducing NK cell dysfunctions [510, 511]. Hypoxic conditions in large adipocytes can decrease NK cell cytotoxic potential by degrading granzyme B, which impairs M2 macrophage destruction [510]. The lack of turnover of M2 macrophages allows them to remain in proinflammatory milieu of IFN-γ inducing the phenotypic profile switch to the M1 profile

exacerbating inflammation [510]. Increased IL-15 secretion by obese adipose macrophages positively induces NK cell metabolism, increasing proliferation and activation [511, 512]. Studies have also shown that in obesity, adipocyte-secreted IL-12 promotes the proliferation of another subset of adipose-resident ILC1s that are phenotypically and functionally distinct from NK cells [510, 513]. This subset of ILC1s exhibits increased cytotoxicity, produces IFN-γ, drives M1 proinflammatory macrophage polarization, and contributes to insulin resistance and adipose fibrosis through an IFN-γ dependent mechanism [510]. Adipose-resident macrophages also express receptors for binding to NK cells thus initiating a viscous cycle that propagates inflammation [477, 479].

6.6.2 **ILC2 cells:** ILC2s, which are among the primary immune cells in lean adipose tissue are activated by IL-25 /IL-33 [514]. ILC2s utilize glycolysis and FAO for energy via the IL-25 /IL-33-mediated upregulation of mTOR. ILC2s also utilize FAO with arginine metabolism, producing polyamines to stimulate glycolysis and cytokine production [514]. The increased uptake and metabolism of amino acids such as arginine and methionine epigenetically reprogram ILC2s to release IL-4, IL-5, IL-9, and IL-13 [515]. ILC2s promote eosinophil-mediated M2 macrophage polarization [516], beige conversion of white adipose tissue (WAT), and beige fat biogenesis (via IL-4, and IL-13) [517-519] maintaining adipose tissue homeostasis. ILC2s also receive multiple inputs from CNS circuits [520] and can also be regulated via PVN-gonadal glial-derived neurotrophic factor (GDNF) to promote energy expenditure and insulin resistance and limit obesity [520-522]. ILC2s are drastically reduced or depleted in HFD-induced obesity, possibly due to increased epithelial cell-derived alarmins (IL-33), which inhibit or dampen ILC2 functions, resulting in significant weight gain and glucose intolerance [523, 524].

6.6.3 **ILC3 cells:** ILC3s are one of the most understudied immune cells in adipose tissue that impact adipocyte homeostasis through IL-17A and the recently discovered IL-22 [525]. ILC3s utilize glucose and fatty acids in quiescent states, which upon activation shift into enriched glycolytic pathways supported by mTOR [526]. Studies have elucidated the involvement of pyruvate, fructose and mannose metabolism in ILC3 activation [526]. Adipose-resident ILC3s cells are important for

maintaining adipose homeostasis via IL-22, which induces WAT beiging, preventing insulin resistance and obesity [527]. Some studies have indicated an increase in the ILC3 population in obese individuals due to chronic inflammation and/or altered production of adipokines and cytokines [527]. However, considering the role of ILC3s in maintaining tissue homeostasis, it is plausible that activity might be negatively affected in the context of obesity, contributing to the overall proinflammatory state. However, definitive conclusions about the regulation of adipose-resident ILC3s in diet-induced metabolic syndrome are still controversial, and more research is needed, given their dual effect on maintaining homeostasis, and adipose-tissue beiging, and their role in diet-induced obesity.

6.7 Integrated adipose tissue immune response: In summary, in lean adipose tissue, the AT stromal cells produce IL-33 that stabilizes T_{reg} and ILC2s. ILC2s help to maintain eosinophils and M2 macrophage polarization along with T_{reg} cells. Treg cells regulate Th1 and Th17 responses, promoting Th2 response, thus maintaining an anti-inflammatory profile. iNKT-released cytokine influence M2 polarization [466]. T_{reg}-mediated activation of Th2 cells induces anti-inflammatory cytokines to promote M2 polarization and differentiation of basophils and eosinophils. Treg-Th2 interactions also promote the B-cell antibody switch to IgG1 and IgE and inhibit Th1 responses, promoting an anti-inflammatory milieu [491]. With impending obesity and MetS, the FFA-induced lipotoxic response also activated NLRP3 and neutrophil response to induce IL-1β production, which supports the M1 macrophage profile. A concomitant senescence profile reduces the number of pre-adipocytes, drastically lowering T_{reg} cell numbers. T_{reg} cell reduction increased the Th1 and Th17 cell response. Th1/Th17 disbalance along with pathogen-associated molecular patterns (PAMPs), LPS, or FFA induce CD8+ T cell and B-cell activation, initiating the proinflammatory response [528]. Altered levels of adipokines such as leptin and adiponectin seen in metabolic syndrome or obesity may also play a role in promoting Th1 and Th17 cells and inhibiting Treg and Th2 cell populations [491, 528, 529]. The reduction of ILC2s, eosinophils, and elevated ILC1s and iNKT cells initiates/supports M1 polarization of macrophages [529], which produce proinflammatory cytokines.

The increased expression of these proinflammatory cytokines further allows the migration of more immune cells, thus initiating a potentially unending chain of inflammation [529]. With obesity, the spill-over of lipids due to dysregulated storage increases plasma free fatty acid levels, which are taken up by immune cells like T-cells, causing lipotoxic cell death. In obese adipose tissue, memory T cells (CD8+CD44+) are activated in response to LPS or antigens presented by APCs, promoting macrophage recruitment and M1 polarization [530]. This change in polarization reduces M2 macrophages, which coincide with dysregulation of innate lymphoid cells (ILCs) and eosinophils in adipose tissue, and limits WAT beiging. Obesity or metabolic syndrome induced by a high-calorie and/or high-fat diet increases saturated FAs (palmitate) in serum, which bind and activate T-cell receptors [531]. This T-cell activation stimulates the inflammasome pathway and upregulates NF-κB, MAPK, and JAK-STAT pathways, culminating in increased secretion of pro-inflammatory cytokines like IL-1β, 18, 6, TNF-α, IFN-γ and granulocyte macrophage-colony stimulating factor (GM-CSF) exacerbating inflammation [531].

7 Chemical messengers – A game of intricate interplay among organs

Various interactions exist between the ever-altering metabolic cycle in adipose tissue and other organs via endocrine mediators. Adipose tissue has long been considered an endocrine organ that releases chemical messengers, namely leptin, adiponectin, adipsin, omentin, resistin, visfatin, irisin, neuregulin 4 (NRG4), nesfatin-1, meteorin-like protein (METRNL), chemerin, and IL-6. The production of chemical messengers depends on the type of adipocytes–white or brown–and their metabolic activity and overall host metabolic profile. Adipose tissue contains many types of cells with various receptors that can interact with small molecules derived from the gut and other tissues, allowing dynamic modulation of adipose tissue metabolism. The dynamic metabolic profile of adipocytes then regulates the secretion of chemical messengers. The endocrine mediators secreted by white adipose tissues or brown adipose tissue, termed adipokines or batokines, are intricately balanced to maintain homeostasis. Normal gut microbiota, such as *Akkermansia muciniphila,* via microbial degradation products such as SCFAs and other metabolites (biliary metabolites) support

lean adipose tissue adipokines that promote fat oxidation, reduce fat mass, and improve glucose utilization [532]. Chemical messenger benefit thermogenesis, increase energy expenditure, improve angiogenesis, and modulate inflammatory responses by improving anti-inflammatory functions. They can also regulate cardiovascular functions by regulating blood pressure, endothelial functions, vascular tone, and macrophage infiltration, thus modulating atherosclerotic events. Many adipokines are also implicated in neurological functions such as mood, cognition, stress, and neurological disorders like depression and schizophrenia [533]. Under lean, homeostatic conditions, adipose tissue produces a greater quantity of anti-inflammatory chemokines and a low quantity of proinflammatory chemokines [533]. However, their production or secretion is altered in metabolically dysfunctional adipose tissues owing to gross alterations in adipose tissue metabolism. One of the earliest adipokines, leptin is secreted by adipose tissue, and is directly proportional to adipose fat mass, increasing BMI. Leptin regulates immune cell function changes associated with obesity via mTOR regulation, which regulates protein synthesis, ribosome biogenesis, and autophagy, modulating proinflammatory immune cell functions [533]. Adiponectin, another adipokine, downregulated in obesity, stimulates AMP-activated protein kinase (AMPK), which regulates energy homeostasis, and glucose metabolism by promoting catabolism and decreasing anabolism antagonistic to mTOR [534]. Interestingly, adiponectin via the adiponectin-AMPK pathway prevents proinflammatory B-cell activation [535], but adiponectin is reduced with high-fat diet supplementation and obesity [535]. Thus, we can deduce that adipose tissue dysfunction alters the chemokine profile, predominantly increasing proinflammatory chemokine secretion and reducing anti-inflammatory chemokine secretion [536] (**see Table 2 for a list of adipose tissue chemical messengers affecting overall systemic homeostasis**). This alteration in the chemokine profile reduces thermogenesis energy expenditure; increases fat mass, glucose intolerance, insulin resistance, and atherosclerosis, and increases the risk of full-blown metabolic disorders..

Table 2: Adipose tissue messengers with metabolic functionality interlinking different organ-functions and dysregulations in obesity/HCHF-induced metabolic syndrome

8 Liver Handling of Metabolites—A story of Abundance

Liver is a major site of metabolism where multiple metabolic signals provide inputs that affect metabolic changes in both well-fed and starved states. In a well-fed state, the liver receives a surplus of monosaccharides, dietary fats (via reminant chylomicrons), and amino acids. Glucose enters hepatocytes via GLUT-2 transporters and is metabolized via multiple pathways, such as glycolysis (which produces pyruvate), HMP shunt pathways (which generates NADPH and ribose), and glycogenesis to produce glycogen (influenced by bile acids and insulin). Glycolysis-generated pyruvate forms acetyl-CoA, which undergoes the TCA cycle for energy generation. Some quantities of acetyl-CoA get diverted to the cytosol via citrate for fatty acid synthesis by *de novo* involving ACLY, AAC, FASN enzymes producing palmitic acid. Glucose flux upregulates carbohydrate response element binding protein (ChREBP) which upregulates *de novo* lipogenesis enzymes [630]. Palmitic acid may be further elongated or desaturated (by SCD) to form a long chain or unsaturated fatty acids, namely stearic acid (C18) and oleic acid (C18:1, D 9) which are then esterified for conversion into endogenous TAGs. Small amounts of FFA enter into FAO pathways to generate ATP to be utilized by hepatocytes for energy purposes. The lipid storage capacity of the liver is minimal; so, the excess FFAs are re-esterified into TAGs, which are then packaged into VLDL and transported to adipose and other peripheral tissues. Minor amounts of cytosolic acetyl-CoA are used for cholesterol biosynthesis (stimulated by ChREBP) [630], which is then utilized to synthesize bile acids, vitamin D, and steroid hormones (either in the liver or other endocrine organs). After delivery of their TAGs in adipose tissue, the reminant chylomicrons reach the liver, where they are finally metabolized and broken down to FFA and glycerol, which can be utilized for TAG resynthesis. Chylomicron resident cholesterol can be used for the biosynthesis of bile acids, or excess cholesterol can be excreted via bile. With the addition of fructose or sucrose in the diet, fructose metabolism in the liver feeds into the glycolytic pathway via fructokinase action [630]. Fructose metabolism also generates glyceraldehyde and dihydroxyacetone phosphate (DHAP) which can continue into glycolysis or feeds into lipogenesis. Fructose-induced stimulation of carbohydrate response element

(ChREBP) subsequently upregulates *de novo* lipogenesis (via SREBP induction and activation) producing fatty acids which are esterified into TAG synthesis and enhances cholesterol biosynthesis [630]. Fructose metabolism can generate uric acid and advanced glycation end products such as methylglyoxal (in higher doses of fructose), which can directly inhibit AMPK thus reducing energy expenditure [631]. Fructose metabolism-generated AGEs via their receptor for advanced glycation end products (RAGE), disrupt mitochondrial functions, increasing the production of ROS production, which interact with RAGEs, and promote oxidative stress [631]. Mitochondrial ROS and fructose (directly) interact with proteins undergoing molecular rearrangements, resulting in the formation of more AGEs, namely carboxymethyl-lysine (CML) and glyoxal-lysine dimer (GOLD) (via albumin binding) [632]. These AGEs inhibit SREBP cleavage-activating protein (SCAP) degradation, triggering unimpeded SREBP activity that induces lipogenesis.

During fasting, the liver generates glucose via glycogen breakdown and produces glucose via gluconeogenesis, with gluconeogenic precursor obtained from adipocyte-derived glycerol (obtained from TAG lipolysis), amino acids from muscles. The liver also metabolizes FFA obtained from adipose tissue lipolysis via FAO to generate acetyl CoA which enters the TCA cycle and ETC to generate energy.

HCHF consumption increases intestinal lipid absorption, which transfers more TAG cargo to adipose tissue and the liver via chylomicrons. Fructose or sucrose consumption with high-fat augments *de novo* lipogenesis [632], leading to greater lipid output from the liver via VLDL, which again delivers more triglycerides to adipose tissues, which can ultimately increase the serum levels of TAGs, VLDLs, and LDLs. HCHF or HFD induced proinflammatory cytokines, which along with increased oxidative stress induces systemic insulin resistance, which reduced adipocyte *de novo* lipogenesis and glucose uptake. The constant uptake of FFA into adipocytes saturates the adipocytes storage capacity which spills-over excess FFAs and TAG into systemic circulation, allowing FFA load to be delivered to the liver [633]. ANGPTL4 produced by the liver inhibits lipoprotein lipase [634], increasing lipid delivery to the liver. Hepatic CD36 overexpression seen in diet-induced obesity

markedly elevates hepatic free fatty acid uptake [635]. One of pathways that have been identified in hepatic metabolism changes in cases of HCHF or HFD with or without fructose consumption is dysregulation of protein tyrosine phosphatases [636]. In HCHF or HDF or fructose ingestion, the gut barrier dysregulation causes transmigration of LPS which play a key role in activating hepatic protein tyrosine phosphatase receptor type-kappa (PTPRK) [636]. LPS and PTPRK also induce aberrant expression of PPARγ, and HCHF or HFD with or without fructose increase hepatic expression of PTPRK-PPARγ induced by LPS [636]. One of the hypotheses is LPS-induces PTPRK which the upregulates c-Fos and STAT-1 promoting PPARγ expression [636]. PTPRK-PPARγ upregulation in hepatocytes increases the expression of CD36 fatty acid transporter, increasing hepatic FFA uptake [636]. The overexpression of PTPRK-PPARγ increases the glycolytic capacity, inhibits the action of fructose-1,6-bisphosphatase enzyme and increases the concentration of glycolytic intermediates like dihydroxyacetone phosphate (DHAP) and glyceraldehyde-3-phosphate (G3P) [636]. PTPRK upregulation also increases acetyl CoA levels by favoring pyruvate to acetyl CoA conversion by dephosphorylating pyruvate dehydrogenase [636]. Increases expression of PTPRK, also reduces the glucose metabolic flux via HMP shunt pathway thus favoring greater glycolytic fluxes and reduces NADPH further impacting the redox balance [636]. The elevated expression of PTPRK, thus promotes accumulation of acetyl-CoA, which now can exit mitochondria, enter *de novo* lipogenesis. PTPRK overexpression also upregulates the expression of lipogenic enzymes such as ACC and FASN thus boosting greater *de novo* fatty acid biosynthesis.

Excessive *de novo* lipogenesis and CD-36 mediated mediate lipid uptake increase FFA in liver, which are then converted into TAG for VLDL synthesis, but the rate of TAG synthesis far exceeds the rate of VLDL synthesis. These events start an accumulation of FFAs and TAGs in hepatocytes causing lipotoxicity which induce ER stress, mitochondrial dysfunction in hepatocytes [635]. Increased *de novo* lipogenesis generates malonyl CoA as intermediate which inhibits carnitine palmitoyl transferase-1(Cpt1) which further impairs FAO, thus potentiating FFA accumulation [635]. Elevated hepatic FFAs cause lipotoxicity, and hepatocytes attempt to reduce lipotoxicity by elongating and

desaturating saturated fats and eliminating them from the liver via lipoprotein synthesis [635]. To circumvent lipotoxicity, *de novo* lipogenesis-derived lipids are diverted toward ceramide synthesis [635]. Increasing intrahepatic ceramide concentrations initiate ER and mitochondrial stress, which causes hepatocyte death by increasing apoptosis [635, 637]. Hepatic sinusoidal endothelial cells (HSEC) are major regulators of macromolecular transport that play a major role in downregulating proinflammatory cytokines and chemokines and lipoprotein transport [635, 638]. High-fat diet induced lipid overload impairs HSEC functions, causing the upregulation of proinflammatory cytokines; and dysregulated lipoprotein metabolism [635, 638]. Hepatocyte lipid overload-induced apoptotic death and HSEC dysfunction produce endocytotic vesicles carrying large quantities of chemokines (chemokine ligand 10) and ceramides which facilitate monocyte chemotaxis to the liver and activate Kupffer cells [635, 638]. Insulin-induced endothelial NO under homeostatic conditions activates HSECs, which then maintain hepatic stellate cells in a metabolically quiescent state [638]. High-fat diet-induced insulin dysfunction and lipid overload reduce endothelial nitric oxide production which causes HSEC dysfunction ultimately activating hepatic stellate cells [605]. Dysregulated HSEC-mediated proinflammatory cytokines (lipotoxic death also upregulates proinflammatory cytokine) and ceramide reduce FAO and lipoprotein synthesis [635, 638]. Saturated lipid buildup promotes alternative oxidative pathways, such as microsomal and peroxisomal oxidation, leading to increased ROS generation [639], exacerbating hepatocyte death, and ECM remodeling, and causing fibrosis (refer figure 6). A high-fat diet containing saturated fatty acids with or without fructose can impair hepatic lipoprotein biosynthesis which increases hepatic lipid content [640]. A high-fat diet elevated hepatic docosahexaenoic acid which via the retinoid X-receptor (RXR) pathway, increases hepatic fibroblast growth factor-21 (FGF-21) production [641, 642]. FGF-21 stimulates ketogenesis, gluconeogenesis, systemic glucose uptake, and WAT browning, indicating that it is a preventive mechanism to reduce lipid overload [616-619]. HCHF- or HFD-induced ER stress and inflammation in WAT inhibit the browning process [230], inducing hypersecretion of FGF-21 leading to FGF-21 insensitivity and subsequently high levels, as observed in NAFLD and MetS

patients [620, 621]. Nutrient overload including glucose- or fructose-induced adipocyte insulin insensitivity, is circumvented in hepatocytes promoting hepatic glucose metabolism and *de novo* lipogenesis lipid accumulation [636].

8.1 Bile acid metabolism and its functions: The liver also produces bile, containing bile acids and salts which is essential for lipid digestion and absorption. The bile also serves as important means of excreting cholesterol and maintaining cholesterol levels in blood. Bile acids (BAs) are produced from cholesterol by Cyt. P450 enzyme. They are then conjugated with glycine or taurine, forming primary bile acids secreted into the small intestine. Dietary fat induces cholecystokinin (CCK) secretion, which stimulates the secretion of bile containing primary bile acids. Following lipid digestion and absorption, the gut microbiota deconjugates and partially converts primary bile acids in the intestine to secondary bile acid derivatives. Primary and secondary bile acids are reabsorbed from the ileum via enterohepatic circulation (95%) to return to the liver, and the remaining 5% is excreted via feces. Upon re-entry, they are conjugated and secreted through bile, with <10% escaping to systemic circulation. Conjugated bile acids are deconjugated by bile salt hydrolases (BSHs) present in *Firmicutes* (*Lactobacillus* & *Enterococcus*), and *Bacteriodetes* [643], and then converted to ursocholic acid, isocholic acid, ursodeoxycholic acid (UDCA), and isochenodeoxycholic acid by *Bacteroides, Clostridium, Escherichia, Eggerthella, Eubacterium, Peptostreptococcus, and Ruminococcus* [644]. They can also be converted to deoxycholic acid (DCA) and lithocholic acid (LCA) by the action of bile acid-inducible genes expressed by *Firmicutes, Bacteroidetes, Actinobacteria*, and *Proteobacteria* [645, 646]. DCA, LCA, and UDCA can also be converted to oxo-, iso-, allo-, sulphated-, and esterified-BAs by *Clostridium, Peptococcus, Fusobacterium* and *Pseudomonas* [647, 648]. The concentrations of secondary bile acids in the bile pool depend on gut microbial diversity, activity, and gut transit time, with studies revealing that elevated DCA levels in bile increase bile hydrophobicity and toxicity in mammalian cells [649]. Microbial production of unconjugated bile acids can cause intracellular acidification, ETC disruption, DNA damage, ribosome function inhibition, cell membrane damage and protein denaturation, accounting for the antimicrobial

activity regulating gut microbial population diversity [649, 650]. Normal homeostatic-adapted gut bacteria and intestinal epithelial cells are resistant to the toxic effects of homeostatic gut primary and secondary BAs. BAs act as dynamic signaling molecules via the farnesoid X receptor (FXR) and G protein-coupled bile acid receptor 1 (GPBAR1/ TGR5) [651]. BAs can exert antimicrobial effects by inducing intestinal regional variability in antimicrobial peptides through transcription factors and chromatin reassembly via FXR, TGR5, and vitamin-D-receptors (VDRs) [652]. DCA exhibits maximal antimicrobial properties on bacterial membranes and promotes increased gut T_{reg}/Th17 balance [651-656]. In rodent models, cholic acid in the diet causes gut microbial phylum alterations, increasing *Firmicutes* (*Clostridia* and *Erysipelotrichia*) populations, indicating the dynamic relationship between bile acids and bacterial population diversity [657]. Bile acids via FXR and TGR5 regulate energy homeostasis, glucose metabolism, and innate immunity boosting Treg and Th17 cells to downregulate the inflammatory response [658]. The significant effects of FXR stimulation include the regulation of hepatic lipid and carbohydrate metabolism, reducing lipogenesis and gluconeogenesis while promoting β-oxidation and glycogen synthesis [659, 660] (Refer Figure 4). FXR-BA interactions also regulate autophagy under normal homeostatic conditions, but overexpression or hyperstimulation increases WAT mass and induces insulin resistance [661]. The bile acid-FXR interaction in ileal enterocytes releases FGF-15 (FGF-19 in humans), which in hepatocytes regulating bile acid synthesis [662]. Bile acids can also promote intestinal GLP1 release and insulin secretion by pancreatic β cells [662] (Refer figure 4). BAs also upregulate UCP-1-mediated thermogenesis in brown adipocytes [662, 663]. BA-TGR5 interaction in macrophages promotes an anti-inflammatory profile and inhibits NLRP3 and NF-κB activation [664]. Bile acids via bile acid receptors in white adipocytes increase mitochondrial biogenesis, metabolic rates, respiratory capacity, energy consumption, mitophagy, and mitochondrial fission, improving overall positive mitochondrial health [664, 665]. BA activity on adipocytes includes increasing glucose uptake, increasing insulin signaling via Akt phosphorylation, increasing GLUT4 expression, and increasing systemic insulin sensitivity [664, 665]. BAs also suppress macrophage infiltration and increase the

production of leptin, FABP4, and adiponectin [653]. Bile acids in systemic circulation regulate dyslipidemia via FXR by increasing HDL uptake and LDL clearance by stimulating LPL [666].

8.2 Bile acid dysregulation due to HFD or HCHF: Western diets increase the population of 7-alpha-dehydroxylase-containing bacteria and increase DCA levels in the bile acid pool [667]. In addition, hydroxyl groups at positions 3, 6 (C6, observed in MCA), 7, and 12 on the steroid backbone affect both their solubility and hydrophobicity [668]. HFD- or HCHF-induced gut dysbiosis alters the composition of the gut microbiome and bile acid metabolites [660, 667]. There is much variability in the results of bile acid profiles and their alterations in MetS or T2DM. However, one common fact is that total bile acid concentrations are elevated in individuals with diet-induced MetS or T2DM with specific alteration observed in the elevation of DCA, CA, and CDCA, which is attributed to the expansion of *Proteobacteria* and *Enterobacteriaceae* and altered *Firmicutes, Bifidobacterium,* and *Clostridium* clusters. This altered gut microbiota increases luminal primary bile acids, reducing secondary bile acids (mainly LCA) and sulfated bile acids [669-671]. This altered BA profile impairs the maximal stimulation of FXR, which is required for the metabolically healthy functionality of bile acids. However, chronic high-fat diet consumption increases intraluminal and fecal BA levels by enriching hydrophobic BAs such as DCA and CDCA. DCA and CDCA induce intestinal hyperpermeability by altering tight junctions via breakdown of tight junctions or via ROS-PI3K-mediated disruption of zonulin, β-catenin, occludin, and E-cadherin [147, 191, 196, 672] (refer figure 5 for HFD- or HCHF-induced biliary metabolism and its effect on gut homeostasis). Elevated DCA in HCHF diets also impacts the population of goblet cells, reducing their populations and reducing mucin production via an FXR-dependent mechanism [673, 674].

Figure 5: Bile metabolism and its regulatory effects on hepatic glucolipid regulation and gut homeostasis (Normal lean gut metabolism of bile acids and their effects are marked in black. HCHF- or HFD-fed altered gut metabolism of bile acids and their effects on hepatic glucolipid pathways are marked in red, red arrows indicates pathways or metabolites reduced or increased in HCHF- or HFD-fed conditions)

Figure 5 Legend: Cholesterol derived bile acids are synthesized in the liver and are secreted into the intestine, where they are modified by gut bacterial enzymes to produce secondary derivatives, that promote gut barrier integrity and immune homeostasis and return to the liver affecting glucolipid metabolism. Some bile acid species escape into the general circulation although major bile species are re-secreted into the bile. In cases of high-fat diet ingestion, an altered gut microbiome alters secondary bile metabolites producing hydrophobic bile that disrupts epithelial integrity, and immune homeostasis and increases hepatic gluconeogenesis and *de novo* lipogenesis.

CA: cholic acid, CDCA: chenodeoxycholic acid, DCA: deoxycholic acid, LCA: lithocholic acid, TGR5: Takeda G-protein coupled receptor 5, FXR- farnesoid X receptor, FGF-fibroblast growth factor, SHP-small heterodimer partner, CYP7A1: cholesterol 7-alpha hydroxylase, FGFR4: fibroblast growth factor receptor-4, DNL: *de novo* lipogenesis, SREBP1c: sterol regulatory element-binding protein 1c, GSK3β: glycogen synthase kinase 3β, EFGR: epidermal growth factor receptor, PI3K: Phosphoinositide 3-kinase [Figure 4 legend ends]

8.3 HFD- or HCHF-induced hepatic inflammation:

The homeostatic gut microbes metabolize BAs to produce secondary BAs which modulate gut immune response, maintain T_{reg}/Th17 balance, but HCHF-or HFD-induced gut dysbiosis alters microbial populations. Gut dysbiosis alters BA metabolites, disrupts immune balance favoring inflammation and increases hepatic LPS exposure. Elevated levels of CDCA and DCA [676] exert a pro-inflammatory effect on neutrophil recruitment and cytokine release from Kupffer cells, initiating the inflammatory response and can induce hepatic CXCL1, CXCL2 chemokine expression in an FXR-independent manner. The normal homeostatic gut metabolite SCFAs maintain gut barrier integrity, but HCHF- or HFD-induced reduction in SCFA lowers gut barrier integrity, increasing hepatic LPS exposure. Butyrate from the gut also exerts anti-inflammatory effect on the immune environment of the liver by stimulating T_{reg} cells and inhibiting Th1/Th17 cells. SCFA also reduces the production of proinflammatory cytokines, promoting the production of anti-inflammatory cytokines and alleviating hepatic inflammation. SCFAs, via their cognate receptors (GPR41 and

GPR43), prevent hepatic lipid accumulation and increase insulin sensitivity, reducing hepatic lipid deposition which is impaired in HFD or HCHF consumption. HCHF or HFD reduces homeostatic gut microbial metabolism of tryptophan into indole and its derivative namely indole-3-propionic acid, which has anti-inflammatory potential, reducing hepatic inflammation by modulating the hepatic immune response [676-678]. The production of serotonin, which is increased in HFD- or HCHF-fed subjects can cause gut hyperpermeability and initiate hepatic inflammation [679]. The adipose-derived chemical messengers such as adiponectin, irisin help maintain normal hepatic functionality, where adiponectin promotes hepatic insulin sensitivity, reduces gluconeogenesis and *denovo* lipogenesis [540, 541] and irisin increases hepatic insulin sensitivity and promotes M2-macrophage polarization, thus limiting hepatic inflammation [582, 586-589].

In high-fat diet consumption, altered bile-acid metabolites, tryptophan, SCFAs, indoles, serotonin levels increase the chance of hepatic inflammation. HFD- or HCHF-induced gut barrier disruption allows the translocation of lipopolysaccharides and gut-derived immune cells initiates an immune response in liver. HCHF or HFD with or without fructose induces increased lipid accumulation in hepatocytes, which initiates hepatocyte ER stress. Systemic insulin resistance increases hepatic *de novo* lipogenesis which increases FFA and ceramide concentrations. Ceramides induce ER stress and mitochondrial dysfunction, further impairing FAO, which accentuates FFA accumulation. Alternative FA oxidation pathways generate ROS, further reducing mitochondrial functionality. Mitochondrial dysfunctions downregulate autophagy, which further impairs hepatic repair [635]. Intrahepatic elevated ceramide concentrations promote hepatic *de novo* lipogenesis via SREBP1, which increases lipid storage, promote NLRP3 activation promoting inflammation [681]. Ceramides induced biosynthetic capacity initiates ER stress, maintains PERK, cathepsin B/D production and regulates autophagy [335, 336, 682]. The activation of NRLP3 (producing IL-1β), increased TNF-α cytokines (HFD-induced inflammation) along with gut-derived LPS-mediated hepatic M1 macrophage polarization reduces hepatic Treg cells via macrophage Notch1-exosomal miR142-3p dependent pathway [683].

The reduction in hepatic Treg activity increases CD8+ T-cell [760] which are pro-fibrotic in nature further increases the risk of hepatic fibrosis [684]. HFD diet also reduces the concentrations of hepatic natural killer T cells (NKT) [684, 685] which are anti-fibrotic immune cells. The involvement of other immune cells has also been predicted in cases of hepatic fibrosis such as Th17, Th1, Th2, MAIT cells although evidence in HFD- or HCHF-induced hepatic fibrosis is still not completely elucidated but they play an important role in hepatic fibrosis [684].

The increases levels of inflammatory mediators, increased ROS production and mitochondrial dysfunction promotes hepatocyte death and stimulates proinflammatory immune cells. The presence of inflammatory mediators such as LPS, proinflammatory cytokines, oxidative stress, lipotoxic hepatocyte death, shifts the balance in favor of inflammation which activates Kupffer cells and hepatic stellate cells, (Refer figure 6 for details on altered HFD-fructose- or HCHF-fructose-induced hepatic metabolism and activation of Kupffer and stellate cells).

Figure 6: Pathophysiology of steatohepatitis induced by high-calorie high-fat diet with fructose consumption

Figure 6A: Altered hepatocyte metabolism induced by HCHF- or HFD-fructose diet where increased influx of lipid species is coupled with increased uptake of FFAs and glucose. Increases hepatic lipid lipogenesis increases hepatic lipid storage. High fructose consumption-induced lipogenesis also increases FFA production. DNL inhibits FAO thus preventing FA degradation contributing to FA storage. Ceramide production in hepatocytes happens to reduce lipotoxicity, induce hepatocyte ER stress which leads to hepatocyte death. Increased saturate fatty acids like palmitate stimulate inflammasome to produce IL-1β.

Figure 6B. with which further promotes hepatic *de novo* lipogenesis inhibits lipolytic pathways promoting greater lipid storage, promotes NLRP3 activation.

Figure 5B: Gut-derived LPS (due to disrupted gut barrier), gut-derived immune cells, hepatocyte death, LPS-mediated M1-macrophage activation, increases ROS all of which activate Kupffer cells which produce more proinflammatory cytokines.

Figure 6C. Ceramides induced biosynthetic capacity initiates ER stress, which induces hepatocyte ballooning via unfolded protein response (UPR). This coupled with activated Kupffer cell released proinflammatory cytokines cause stellate cell activation, which promotes deposition of ECM materials like collagen, fibronectin via sonic-hedgehog (Shh) mediated pathway.

LPS: lipopolysaccharides, ROS: reactive oxygen species, ACLY: ATP-citrate lyase, ACC: acetyl CoA carboxylase, FASN: Fatty acid synthase, SDH: succinate dehydrogenase, DHAP: dihyroxyacetone phosphate, Gln: glutamine, Glu: glutamate, α-KG: alpha-ketoglutarate, α-SMA: alpha-smooth muscle actin, Col: collagen, YAP-1: yes-associated protein-1

[Figure 6 Legend ends]

The activation of hepatic Kupffer followed by stellate cell activation [635, 660, 686-691] promotes hepatocyte death with increased deposition of ECM materials such as collagen, and fibronectin, causing fatty steatosis [684] with ballooned hepatocytes (UPR mediated) leading to irreversible nonalcoholic steatohepatitis (NASH) or metabolism-associated fatty liver disease (MAFLD) which progressively reduces hepatic functionality.

9 Metabolic syndrome and hypertension – Cause of concern

One of the other significant complications of metabolic syndrome is systemic hypertension, which is characterized by elevated systolic blood pressure (SBP) and diastolic blood pressure (DBP), increasing the risk of adverse cardiovascular events. Blood pressure regulation is a closed system with feedback loops that correct any change in blood pressure from normal limits. Blood pressure regulation involves three dynamic systems: vascular tone and endothelial functionality, the sympathetic and parasympathetic nervous system, and the renin–angiotensin II–aldosterone mechanism. There are sex-based differences in blood pressure regulation attributed to the presence of male-specific sex hormones, namely testosterone, and the female sex hormone estrogen [692]. There are also indications that circadian rhythm, quality of sleep, and exercise all play a part in blood pressure regulation. In normal homeostatic subjects, SCFAs produced by the intestinal microbiota activate the cognate receptors GPR41 and olfactory receptor 78 (Olfr78), which can regulate blood

pressure. Acetate (one of the SCFAs), binds to GPR41 in vascular endothelial cells and induces calcium release, promoting vasodilation [693]. SCFAs can also directly modulate sympathetic nervous activity via GPR41, thus reducing blood pressure [693]. SCFA exerts anti-inflammatory effects through the modulation of T_{reg} action, reducing proinflammatory cytokine production, which improves endothelial functionality and maintains insulin sensitivity [693]. Insulin-stimulated nitric oxide release plays a significant role in maintaining vasodilation and blood pressure [694]. SCFAs, especially butyrate, reduce the levels of ROS, improving endothelial function [693]. SCFA, acetate via GPCR ligands, suppress IL-1 and renin–angiotensin–aldosterone system, reducing blood pressure [695]. Microbiome-produced indole metabolites such as indole-3-acetic acid and indole-3-propionic acid improve vascular endothelial cell functionality and reduce blood pressure [696]. The adipokines/batokines generated by WAT or BAT can also regulate endothelial functionality and thus blood pressure. In peri-vascular adipose tissue, one of the earliest discovered adipokines, adiponectin, can increase vasodilation via nitric oxide synthesis, which is mediated by the adiponectin-AMPK-eNOS pathway [697], and regulates blood pressure. Leptin, another adipokine proportional to fat mass, can influence the sympathetic nervous system [698].

Hypertension involves an increase in both SBP and DBP, which can be attributed to reduced vasodilation, increased vascular tone, increased peripheral resistance, and sustained activation of the sympathetic nervous system or renin-angiotensin II system. The consumption of HCHF- or HFD-with fructose alters the normal homeostatic profile of multiple organs all of which culminate in causing hypertension. HFD-or HCHF-fructose consumption induces gut dysbiosis and alters the profile of gut microbes metabolites such as bile acids, tryptophan metabolites, and SCFAs, especially butyrate. HFD- or HCHF-fructose diet induces gut microbial changes, which increase gut TMAO production which independently can induce vascular dysfunction and promote hypertension [158]. HCHF- or HFD-mediated adipose hypertrophy generates hypoxia and increased adipocyte vascular stromal cell lactate production, which when increased impairs vascular tone in inter-muscle arterioles, inducing endothelial dysfunction [699].

HCHF-or HFD-induced adipocyte expansion reduces levels of adiponectin, and increases levels of leptin, resistin, TNF-α, and visfatin, which can upregulate the expression of angiotensin-II [700], thus activating the renin-angiotensin system, and can also increase the sympathetic activity (mainly by leptin) [538]. The HCHF- or HFD-fructose induced inflammation can increase the serum levels of proinflammatory cytokines like TNF-α, IL-1β, and gut-derived toxins like LPS which can stimulate an inflammatory environment and stimulates ROS production (by NADPH oxidase system), inducing inflammation (via TLR4), which induces endothelial dysfunction, causing hypertension [695].

HCHF- or HFD- causes a reduction in the production of SCFAs, indole derivative and increasing kynurenine and serotonin production, all of which induce vascular dysfunction and promote hypertension [695, 696].

The presence of fructose in the diet complicates this issue by increasing the production of fructose-induced radicals and uric acid, which independently or collectively predisposes to the development of hypertension [701]. Activation of the renin-angiotensin system promotes vasoconstriction, increasing vascular tone. High-fat diet-induced insulin resistance and fructose-mediated uric acid reduces endothelial nitric oxide concentrations, preventing vasodilation. The prevalent vasoconstriction with vascular dysfunctions, loss of vasodilatory properties together play a role in increasing peripheral resistance and vascular tone, causing hypertension (refer figure 7 for pathophysiology of hypertension).

The existence of hyperglycemia due to insulin resistance increases advanced glycation end (AGE's) products, which coupled with elevated oxidative stress promotes endothelial dysfunction. AGE interaction with receptor for AGEs (RAGE) further increase the expression of VCAM-1, selectins, PAI, and increases endothelial permeability (refer figure 6 for more details of AGE-RAGE interaction in hypertension) increases the risk of cardiovascular problems by promoting LDL oxidation-induced atherosclerosis [702] (Refer Figure 7). Elevated ceramide levels may also play a role in cardiomyocyte death, causing heart dysfunction or failure [702]. The presence of dyslipidemia with increased LDL, the risk of ectopic lipid deposition in the heart, and elevated ROS levels predispose

the subjects to an atherosclerotic event primed by a hyperactivated immune system. ROS and proinflammatory cytokine-mediated cardiomyocyte damage may further impair cardiac functionality, causing cardiomegaly and increasing the risk of hypertension and cardiovascular failure.

Figure 7: Pathophysiology of hypertension and cardiovascular issues in individuals with diet-induced metabolic syndrome

Figure 7 legend:

Figure 7A: HCHF- or HFD-fructose induced metabolic abnormalities in the gut increases proinflammatory cytokines, TMAO production. Adipocyte hypertrophy and altered adipokines induced by HCHF- or HFD-fructose upregulates angiotensin-II expression with increased spill-over of lipids increasing plasma lipids (namely FFAs, VLDLs, LDLs) concentrations and reducing HDL concentrations. Adipose tissue hypertrophy also increases proinflammatory cytokine production. Increase fructose intake and subsequent hepatic metabolism increases uric acid which affects vascular properties. HCHF- or HFD-fructose induced insulin resistance causes hyperglycemia and increased advanced glycation end products (AGE).

Figure 7B: Increased FFAs, and FFA-induced ROS induce vascular dysfunctions which coupled with lower vasodilation due to lower nitric oxide (NO) concentration impairs vascular functions. AGEs interact with their receptor (RAGE) to work via PLC-ERK pathways to induce vascular fibrosis increasing vascular tone and peripheral resistance, which induces hypertension. AGE-RAGE interactions also induce epithelial permeability which along with oxidative stress increases the risk of LDL oxidation and atherosclerotic event. Dyslipidemia induced ectopic lipid deposition (cardiac cells), hypertension, atherosclerosis all cumulatively increases cardiovascular disease risk.

PLC: phospholipase C, ERK: extracellular signal-regulated kinase. AP-1: activator protein-1, TGFβ1: transforming growth factor beta-1, PI3K: Phosphoinositide 3-kinase, Rho: Rho GTPases, TMAO: trimethylamine N-oxide

10 Epigenome and metabolic Syndrome- Cause or Effect- An improbable question to answer

The effects on dietary alteration on metabolic pathways is well-known and we can see how increased fats or carbohydrates, or fructose can alter metabolic pathways via regulation of enzymes or cellular signalling pathways. However, the diet-genome interaction is quickly gaining importance, studies focussing on how different types of diets alter the relative concentrations of plasma or cellular metabolites which can alter genomic expression of genes. These types of alterations affect the chromatin network and mRNA expressions collectively altering the expression of genes. The changes are termed epigenomic changes defined as modification of gene expressions without altering the DNA sequence. The importance of epigenome and metabolome has come to the forefront in the past decade, where much change in the metabolic profile is attributed to epigenomic changes or vice-versa induced by nutrition, lifestyle, or environmental interaction. Studies have illuminated the metabolic reprogramming in diseases like cancers, and the impact of metabolites on the activity of chromatin-modifying enzymes has also been identified [703]. The epigenetic regulation of cellular functions can be achieved by two broad mechanism- one being alteration of chromatin network via histone modifications and the other using non-coding RNAs (namely micro-RNAs (miRNAs) and long non-coding RNAs (lncRNAs)).

10.1 Epigenomic regulation via histone modifications: Histone modifications involve a wide array of biomolecules which may be attached to histone to alter the chromatin structure. The various moieties can modulate chromatin network involve addition or removal of methyl or acetyl groups, ubiquitin-like modifier (SUMO) protein (SUMOyl groups) or prenyl groups to name a few. Majority of the chromatin modifiers are either obtained from diet or are produced by endogenous metabolism.

In macrophages, homeostatic IL-4 stimulation, signals the Akt/mTORC1 pathway to induce ATP citrate lyase, which increases cytosolic acetyl-CoA, enhancing histone acetylation and the expression of IL–4 supporting genes [704]. Obesity-induced increase in acetyl-CoA promote protein acetylation and posttranslational protein modifications [705], which result in the transcription of proinflammatory genes [706]. In obese mice and human patients, elevated acetyl-CoA can also alter the levels of lysine deacetylases (KDACs) [707] in adipocytes, which explains the alteration in sirtuin 1 (SIRT1) (Class

III KDACs) that impair adipose tissue homeostasis. Studies have also indicated that, in obese individuals, elevated proinflammatory cytokine downregulate SIRT1 in adipocytes initiating insulin dysfunction [708]. However, in contrast to that in dendritic cells, SIRT1 downregulation in obese individuals induces kynurenine metabolism in T-cells, favouring an increase in Th1 cell populations [708]. SIRT1 expression is negatively correlated with BMI, macrophage infiltration and proinflammatory gene expression [707, 709, 710]. Other KDACs that are altered in obese adipocyte include, KDAC5 and 6 (Class II KDACs), which are downregulated in WAT causing increased proinflammatory adipokines expression and impaired glucose uptake [705]. The epigenetic changes induced by metabolites illuminate nutrient–epigenome interactions in metabolic syndrome, interlinking calorie overnutrition or high-fat diet-induced metabolic maladies with sirtuins.

Epigenomic modifiers such as HDACs also regulates homeostatic functions, for example HDAC3 activity is required to mediate cold-induced thermogenesis in BAT through upregulation of mitochondrial functions, whereas in WAT, HDAC 3 ablation can induce WAT browning [711]. Intestinal HDAC3 interacts with the gut microbiome to regulate intestinal lipid uptake, and ablation of intestinal HDAC3 attenuates high-fat diet–induced elevated lipid absorption [712]. This finding also indicates that the action of chromatin–modifiers is anatomical location specific, with varied functionality in each organ indicating that newer drug approaches can target location specific epigenetic enzymes for metabolic well-being.

10.2 Epigenomic regulation via non-coding RNAs: The other mode of epigenomic regulation involves regulatory RNAs, which includes miRNA and lncRNA produced by cells or tissues under homeostasis or in response to altered metabolic conditions. Many studies have described multiple regulatory RNA alterations in diseases, such as hyperinsulinemia, T2DM, obesity, or hypertension. However, most of the results are in individual metabolic maladies, yet we still lack a unified catalog of regulatory RNAs that would be altered in metabolic syndrome, a conglomeration of all the symptoms. Serum or plasma miRNAs are derived mainly from circulating exosomes, which are lipid bilayer-covered vesicles released by tissue containing a cargo of mRNAs, miRNAs, rRNAs,

lncRNAs, DNA, lipids and proteins (which include CD36, CD9, CD81, heat-shock protein-70 (Hsp70), Hsp90, common cytoskeletal proteins, albumin, and major histocompatibility compex-I (MHCI)). The cargo content depends on the metabolic status of the tissues, where altered metabolic reprogramming in MetS/obesity/T2DM can also alter the exosomal components, such as the miRNA or lncRNA subtypes.

Adipose tissue is one of the primary sources of exosomes with cargo component that regulate metabolic pathways, which can vary according to lean or obese states. In lean obese adipose tissue, a large population of adipose-derived stem cells that produce exosomes containing high concentrations of miR-21 (which promotes angiogenesis via HIF-1α, Akt, ERK, and SDF-1) and miR-10a (promotes T_{reg} and Th17 differentiation) [713, 714]. Lean adipose tissue also contains a large population of M2 macrophages, which produce exosomes containing miR-690 [715], enhancing insulin sensitivity in HFD-fed mice. WAT also produces exosomes containing miR210/92a, which increases FGFR1 expression in BAT, affecting thermogenesis under hypoxic conditions [716]. WAT-derived exosomes from lean mice can act at the hypothalamic level, reducing appetite and body weight in HFD-obese mice [717]. In contrast, BAT exosomes contain miR-132-3p, which reduces hepatic lipogenesis by reducing SREBP-1 [718], and BAT-derived exosomes attenuate HFD-induced metabolic syndrome in animal models [719], which indicates that robust crosstalk between tissues via exosomes can affect energy homeostasis. Adipose tissue exosomes can also modulate macrophage activation and the production of proinflammatory cytokines such as IL-6 and TNF-α via increased obese adipose exosomal miR-34a's, which inhibits M2 polarization and positively correlates with insulin resistance [720]. High-fat diet–induced obese visceral adipocytes secrete exosomes that are rich in miR-132/212 levels, which increases the risk of atherosclerosis by promoting endothelial apoptosis, and vascular smooth muscle proliferation indicating that a high-fat diet can decisively alter exosomal cargoes [721].

Circulating miRNAs derived from endocytotic vesicles are important mediators of crosstalk, and miR-155 has recently been identified as a predictive biomarker for T2DM development in obese

populations [722]. Obese adipose tissue releases exosomes rich in miR-155 that promote proinflammatory cytokine such as IL-6 and induce insulin resistance [722]. WAT-derived exosomes from obese individuals show an altered miRNA cargo profile with elevated levels of miR-222, and 27a and reduced levels of miR-141-3 [723-725]. miR-222 promotes insulin resistance in skeletal muscles and the liver, and miRNA-27a downregulates PPAR-γ in skeletal muscle, inducing insulin resistance, whereas miR-141-3 promoted glucose uptake and boosted insulin sensitivity in liver via inhibition of PTEN [724, 725]. These results indicate that adipose-derived alterations in exosomal cargo may be the real culprits involved in inducing system insulin resistance long before lipotoxicity induces insulin resistance. The prevalent downregulation of sirtuins in BAT in individuals with obesity reduces thermogenesis, but the circulating exosomal cargo concentrations of sirtuins are also reduced which may explain the endothelial dysfunction observed in high-fat diet–induced obese subjects [726, 727]. The downregulation of sirtuins may also be partially responsible for the altered exosomal cargo compositions observed in cases of obesity [728]. There are also reports that insulin-insensitive obese adipocytes release Shh (sonic-hedgehog)-containing exosomes that induce M1 macrophage polarization and exacerbate inflammation [729]. In rodents, miR-33 deletion can cause weight gain and insulin resistance irrespective of normal chow or high-fat diet feeding, indicating that its agonism could be good target in maintaining insulin sensitivity even in cases of HFD [730].

In this contecxt, adipose tissue is not the only source of exosomal miRNAs; other tissues can also impact metabolic programming. Lean hepatocytes release exosomes with miR-130a-3p, which targets adipocytes to inhibit adipogenesis (by decreasing FASN, and PPAR-γ), and increasing insulin sensitivity by increasing GLUT-4 translocation [731]. miR-130a-3p which also plays a cardioprotective role by reducing TNF-α, IL-6, VCAM-1, ICAM-1 and E-selectin expression is downregulated by hyperglycemia and obesity [732, 733]. Another study demonstrated the importance of pancreatic beta-cell exosome-derived miR-26a, which improves insulin sensitivity, beta-cell function and reduces glucose-stimulated insulin secretion (GSIS) but is downregulated in obese humans and rodents due to overnutrition induced beta-cell hypertrophy [734]. The exosomes in MetS

patients also exhibit elevated levels of miRNAs like hsa-miR-122-5p, hsa-miR-124-3p, hsa-miR-222-3p, hsa-miR-194-5p, hsa-miR-101-3p, hsa-miR-19b-3p, hsa-miR-27a-3p, hsa-miR-149-5p [735]. The importance of miR-143-3p, which is positively associated with IR, has also been described where high serum levels of miR-143-3p increase the risk of development of MetS by six times as compared to usual or lower miR-143-3p levels [736]. One of the recent studies in the Qatari population with MetS has identified downregulation of miR-153-3p (sphingolipid metabolism), miR-182-5p (negative correlation with T2DM duration), and miR-433-3p (HbA1c related) [737]. The role of lncRNAs has also investigated in metabolic syndrome, where the lncRNA-XIST/PTEN axis was found to be reduced while miR-214-3p was elevated in the peripheral mononuclear cells of MetS patients compared with those of standard controls [738].

Even though much information exists about miRNAs and lncRNAs, still a composite map of miRNAs, lncRNAs and their metabolic targets and their alterations in metabolic syndrome that can be extrapolated to the pathophysiology of the symptoms is deficient. The link between metabolism and epigenomic alterations helps us venture into new therapeutic intervention avenues.

11 Conclusions and future perspectives: There are many studies on singular pathophysiologies, such as obesity, insulin resistance, and dyslipidemia. However, a complete pathophysiological roadmap explaining the causes and effects of metabolic syndrome is necessary to develop new therapeutic approaches. Multiple reviews with the keywords "metabolic syndrome" and "review" have been published recently. However, they still unable to present a pathophysiological representation, with some focusing on intestinal endotoxemia, nonalcoholic (metabolic) fatty liver disease, or oxidative strategies to target metabolic syndrome. Some studies have presented an adipocentric view illuminating adipose tissue maladaptation, whereas few have discussed the cardio-renal-hepatic axis in the pathophysiology of metabolic syndrome. Our review focuses on a multicentric view of how a high-fat- or high-calorie high-fat-sucrose diet facilitates faster absorption of nutrients, which stresses the intestinal homeostatic partners and leads to changes in the gut microbial populations. We also highlight how high-fat-induced mitochondrial perturbation induces gut dysbiosis and the aftermath of

this gut dysbiosis on the altered metabolism of gut metabolites such as short-chain fatty acids, indoles, and bile acids, which are predominantly anti-inflammatory.

The focus on enterocyte lipid absorption via lipase enzymes also prompts us consider probiotic prescriptions that can be used to regulate host-lipid absorption. This review also highlights the use of probiotics containing *Bifidobacterium or Akkermansia* to alleviate diet-induced intestinal maladaptation. This review also discusses how these gut-derived metabolites increase insulin sensitivity, promote glucose utilization, and reveal how an altered gut dysregulates insulin action and glucose metabolism. This review also discusses how increased lipid delivery to adipocytes exceeds the storage capacity of adipocytes while inducing immune-metabolic profile changes in adipose-resident immune cells, which was not discussed in earlier reviews. We also focus on how differential autophagy regulation may be important for tissue repair, regeneration, and maintenance of tissue homeostasis. This review also reviews on how different pathophysiological mechanisms in diet-induced obesity can affect insulin sensitivity and its effect on vascular metabolism. We also discusses different cells or organelles in adipocytes like the peri-adipocytes and peri-droplets mitochondria associated with lipid droplets, opening new avenues on how lipid-droplet-associated mitochondria affect adipose homeostasis. The review also focuses on different metabolic profiles observed in white, brown, and beige adipose tissues, focusing on how a high-fat diet induces alterations, allowing a better understanding of adipose tissue in metabolic syndrome.

Ceramide synthesis happens to circumvent lipotoxicity, which has been shown to induce insulin resistance, but recent studies indicate that ceramide synthesis precedes high-fat diet-induced adiposity or hyperglycemia in primates. Saturation of storage capacity in adipocytes leads to the ectopic storage of lipids in nonadipose tissue, like the liver, heart, and blood vessels, which impacts their functionality. Our review also sheds light on the hepatic handling of nutrients in lean and overfed conditions and how chronic overfeeding ultimately increases hepatic lipid storage, which initiates fatty liver. We also look at how immune cell interactions mediate the progression of fatty liver to

steatohepatitis. The reviews list a few miRNAs (exosomal and circulating) altered in cases of high-fat diet consumption. However, scientific data on non-coding RNA altered in metabolic syndrome, which were not present in earlier reviews interlinking specific miRNA profiles with their metabolic profile observed in metabolic syndrome, are scarce.

The future implications of this review should focus on how we can modulate the gut microbiota to reduce dietary lipid absorption or find newer targets that alter the junctional integrity of lymphatic channels to modulate lipid absorption. Future directions should focus on factors promoting depot-specific autophagy regulations and how mitophagy targets can be initiated into treatment to increase brown or beige adipocyte activity and metabolic health. Multiple regulatory controls, such as RNA interference, complicate human systems. The current data on miRNAs, although they exist, are insufficient due to the difficulty in extracting exosomes. However, future research should focus on tissue-specific exosomal cargo analysis in the context of a high-fat diet, which will help us design better treatment strategies involving miRNAs. Information about lncRNAs is very scarce, and the use of a multicentric approach across different geographical locations in metabolic syndrome might help researchers understand how long noncoding RNA plays a role in metabolic syndrome.

Thus, this review aims to increase awareness of dietary impacts on gastrointestinal physiology and how these impacts contribute to the pathogenesis of metabolic syndrome and provide additional information about designated targets that would be useful for reducing the incidence of diet-induced maladies.

Acknowledgement

The authors acknowledge the support of Manipal Academy of Higher Education (MAHE), Manipal literature access for data collection. The authors acknowledge the support of Manipal Academy of Higher

Education (MAHE) Manipal for procuring tool for determining the overall similarity index.

Authors' contributions

All authors conducted data research for the article, significantly contributed to the discussion of the content, and reviewed and edited the article prior to submission. BD and AT contributed equally for designing the outline of the article, collecting the data, writing the manuscript, and authoring the article. APR and SUK contributed to the overall design, corrections, proofreading and coauthorship of the article.

Funding

Open access funding was provided by Manipal Academy of Higher Education, Manipal. The authors declare that there is no funding for the manuscript.

Data availability

No datasets were generated or analyzed during the current study.

Declarations

Ethics approval and consent to participate

Not applicable.

Consent for publication

Not applicable.

Competing interests

The authors declare no competing interests.

Gut Microbial Species	Functions in lean homeostatic gut	Altered In
Bacteriodetes and *Firmicutes*	↑ *Bacteriodetes/Firmicutes* ratio	HFD diet ingestion ↑*Firmicutes (*↑ *Erysipelotrichi* populations cause gut inflammation*) and Bacilli*), ↓*Bacteriodetes* [64]
Akkermansia muciniphila	↑ In lean gut maintains mucin production, acetate producers, regulates anti-inflammatory cytokine IL-6 and IL-10, increases tight junction proteins [65]	↓ or ↑ In HFD [66, 67] (but supplementation reduces adiposity in HFD)
Bifidobacterium (Bifidobacterium	Acetate producers from pyruvate [69],	↓ Bifidobacteria in obesity or HFD [71, 72]

longum and *Bifidobacterium bifidum)* [68]	*Bifidobacterium* regulates gut amino acid and bile acid metabolism [70]	
Lactobacilli (*Lactobacillus paracasei*) [68]	Lactate producing species [69]	↓ in HFD, Lactobacillus intervention alleviated HFD-induced obesity [73]
Parabacteroides [74]	SCFA producers (acetate, propionate & butyrate), Anti-inflammatory, Succinate producer, promotes tight junction stability [74]	*Parabacteroides* alleviates HFD-induced obesity & insulin dysfunctions [74, 75]
Clostridium, Lachnospira, Anaerotruncus and *Streptococcus* spp. [76-78]	SCFA producers [78]	*Anaerotruncus & Lachnospira* increases in HFD or obesity [79, 80]
Bacteroidetes (*Prevotella bivia, Alistipes indistinctus, Prevotella corporis*) [78]	Propionate producers from succinate [78, 81]	Reduced in HFD induced obesity [82]
Veillonella spp [78]	Propionate producers from lactate via succinate [78]	Increased in obesity [83]
Megasphaera, Lachnospiraceae, and *Coprococcus* [78]	*Megasphaera* – Propionate producer *Lachnospiraceae*, *Coprococcus*- propionate and butyrate producers [78]	*Megasphaera elsdenii* is reduced in HFD [84] , *Lachnospiraceae* is increased in HFD [85] Coprococcus attenuates high-fat diet induced inflammation, reduced in high-fat diet & NAFLD [86]
Roseburia (Roseburia inulinivorans), Ruminococcus and Odoribacter [78, 87, 88]	Propionate producers [78]	*Roseburia* reduced in HFD or obesity, *Odoribacter* increased in HFD or obesity, *Ruminococcus* (controversial) [87]
Eubacterium rectale, *Faecalibacterium prausnitzii* and members of the clostridial cluster I, III, XV, and XVI [78, 88].	Butyrate synthesizers [78, 89]	*Faecalibacterium prausnitzii* is reduced in HFD or obesity [90] Clostridium has dual role with *Clostridium butyricum* [91] increased in obesity *and C. tyrobutyricum* is obesity protective [92] Eubacterium rectale reduces in HFD [93]

Fusobacterium spp., *Acidaminococcus, Spirochaetes*	Butyrate producers from amino acids like lysine, glutamate, and 4-aminobutyrate pathways [88]	*Fusobacterium* & *Acidaminococcus* increased in obesity or HFD [87, 94, 95]
Clostridium	*Clostridium butyricum* high in lean gut-produces butyrate [78]<br>↓ in lean gut	↑ in obesity or HFD [79], *Clostridium butyricum* attenuates HFD-induced inflammation [96]

Table 1: Different microbial species found in the gut during lean homeostatic conditions and altered profile associated with high-fat diet consumption

Signal	Released by	Target Organ	Action	Expression in Obesity/metabolic syndrome/T2DM
Leptin	WAT (mainly), BAT (regulated by glucose and insulin) [537]	CNS, Hepatocytes, β-cells	• ↓Food intake (hypothalamic actions), affects hedonic eating centers, ↑ Energy expenditure (by stimulation of sympathetic nervous system) [538]	Increased (but inactivity or lowered sensitivity) [537-539]

			• Promotes glucose uptake and thermogenesis (via thyroxine upregulation in BAT [538, 539]<br>• Inhibits Insulin-stimulated glucose uptake in WAT [506]<br>• Increases lipolysis in WAT [538, 539]<br>• Increases insulin release via hypothalamic-pituitary axis (adipoinsular axis) -bidirectional connections with insulinemia increasing leptin secretion [537, 538]	
Adiponectin	WAT (inversely proportion to fat mass) (secretion higher in female than male probably due to estrogen effects on adipose tissue) [540]	Heart, Brain, Liver, Skeletal muscles, pancreatic cells	• Activates AMPK (viaAdipoR1 receptor) and promotes glucose uptake and FAO in muscles [540-542]<br>• Increases mitochondrial related genes, reduces oxidative stress [541]<br>• Reduces hepatic gluconeogenesis, lipid synthesis and reduces risk of NAFLD [540, 541]	• Decreases in obesity and metabolic syndrome patients due to oxidative stress, insulin resistance, TNF [540, 543, 545]<br>• High-fat diet in rodents reduce

			• In skeletal muscle, promote glucose uptake, ↑ FAO, ↑ Insulin sensitivity (via Ca^{2+}-AMPK or AdipoR2-PPAR pathways) [540, 542]<br>• Maintains blood pressure, cardioprotective (increases cardiac OXPHOS) [540]<br>• Promote adipogenesis, increase adipocyte number, adipocyte browning (maybe?) [543].<br>• Promotes insulin synthesis and exocytosis [542, 544]	adiponectin mRNA [541]<br>• Metabolic syndrome patients with high BP females have greater reduction in adiponectin than males [541]<br>• Reduction increases metabolic syndrome incidence [545, 546]
Resistin	Both WAT and BAT in rodents, and leukocytes in humans [547, 548]	Liver, Brain, Adipocytes, monocytes	• Upregulates pro-inflammatory (IL-6, TNF-α) gene expression in monocytes, regulate monocytes/macrophage function, increase pro-inflammatory cytokine like IL-6, IL-12 via MAPK signaling pathway [547-549]<br>• Participates in cellular stress response [548]	High-fat diet and obese rodents show high resistin levels [548]<br>LPS in rodent models increase serum resistin [548]<br>Increased in obesity (strongly corelate with insulin resistance only in obese,

			• Corelates with insulin resistance, resistin downregulation improve insulin sensitivity [547, 548]	T2DM patients with hyperresistinemia) [549, 550]
Omentin-1	Visceral WAT (Stromal Vascular cells) , mesothelial cells, vascular cells [551]	Liver, Brain, Adipose tissue, Endothelium, Heart	• Promotes vasodilation (via NO), anti-atherosclerotic, improves cardiovascular functions [552, 553-557]<br>• Anti-inflammatory, ↓ NF-κB, ↓TNF-α, ↓IL-6 [552]<br>• Reduces oxidative stress, mitochondrial dysfunction in LPS-stimulates macrophages [552, 553]<br>• Increase insulin sensitivity via PPAR-γ [552, 554, 556]<br>• Promote glucose uptake, ↑ FAO (via AMPK) [552-558]<br>• Hypothalamic effect on feeding/satiety neurons (CART. CRH) [552]	Decreased in metabolic syndrome, negative correlation with body mass, insulin resistance [552, 554, 555, 557-559] Elevated Insulin and glucose reduce Omentin-1 secretion [554]

Adipsin	Adipose tissue [559]	Pancreatic β-cells, Heart, Liver	• Preserves beta-cell function in mice and prevents T2Dm in humans [560]	Decreased in T2DM and obesity [550, 561], higher expression on SAT compared to VAT [562]<br>Increased adipsin seen in acquired partial lipodystrophy [563], in T2DM [562]<br>Higher in patients with Obesity and T2DM [564]
Visfatin (extracellular)	Macrophages, chondrocytes and amniotic epithelial cells and adipose tissue	Liver, Muscles, Kidneys	• Pro-angiogenic, Proinflammatory via NF-κB stimulation [565 –569] can induce endothelial dysfunction<br>• Induces hepatic insulin resistance [569]	Increased in metabolic syndrome [570]
Retinol binding protein-4 (RBP-4)	Hepatocytes and adipocytes in mice [571]	Liver, Macrophages, Adipocytes	• Impair adipose insulin signaling via macrophage activation (NLRP3 inflammasome in macrophages) [571-574]<br>• Promotes lipolysis in adipocytes and induced hepatic insulin	Elevated in metabolic syndrome independent of gender, positively associated [571]

			resistance by SOCS3 [571, 572]<br>• Induces insulin resistance in skeletal muscles via IRS1 inhibition, and increases glucose production in the liver by gluconeogenesis and adipose tissue insulin resistance [571-573]	
Monocyte chemoattractant protein–1 (MCP-1)	Macrophages, endothelial cells, epithelial cells, fibroblasts, smooth muscles [575]	Immune cells, Liver, Skeletal muscles	• Potent chemotactic factor for monocytes [575]<br>• Induce insulin resistance in liver and skeletal muscle [575]	Increases in T1DM, obesity and insulin resistance [575] No significant change in children with obesity, T1DM [576]
TNF-alpha	Multiple immune and non-immune cells [577]	Liver, Adipocytes, Brain, Heart, Pancreas, Skeletal muscles	• Proinflammatory [577]<br>• Mediates insulin resistance [578]<br>• Promotes carbohydrate dysregulation, reducing glucose clearance, promotes FAO [578]	High-fat diet increases TNF-α [578, 579] Increased in obese, metabolic syndrome [580]
Irisin	Skeletal muscles, Pancreatic beta-cells, WAT, myocytes [581, 582]	Multiple tissues, osteoblasts, adipocytes	• Promotes WAT beiging [582-585] and increase BAT thermogenesis<br>• Regulates Feeding behavior [582]	Correlation with metabolic syndrome is unclear but positive association

			• Reduces hepatic insulin resistance, body weight, and blood glucose levels, inhibits gluconeogenesis, and stimulates glycogenesis [582]<br>• Reduces pro-inflammatory cytokines and increases anti-inflammatory [586-588]<br>• Mediates macrophage proliferation and induces M2 polarization [589]	between irisin levels and fasting blood glucose, HOMA-IR and waist-to-hip ratio (WHR) [590].
Neuregulin-4	BAT [591]	BAT, other adipocytes, Liver [592]	Increases BAT thermogenesis and attenuates hepatic steatosis, inhibits inflammation, and improves glucose metabolism [592-595]	Lowe circulating levels in metabolic syndrome [596]
Nesfatin-1	Hypothalamus [597]	Hepatocytes, BAT	• Activates AMPK, attenuates PPAR-γ- and reduces the expression of lipogenesis-related genes [597]<br>• Activates BAT-UCP1 facilitating thermogenesis [597]<br>• Induces satiety, reduces food intake [597, 598]	Reduced in metabolic syndrome, biomarker for pre-diabetes [599-601]

			• Improves glucose metabolism [598]	
Chemerin	Liver, Adipose tissue (BAT) and placenta [602]	BAT, Endothelial cells, Macrophages, female reproductive system, lung, muscle tissues, endocrine tissues	• Chemerin overexpression impairs homeostasis, induces glucose intolerance and negative regulator of thermogenic beige fat [603]<br>• Correlates positively with LDL and negatively with HDL [602]<br>• Chemerin induces vasoconstriction via cAMP reduction, increased ERK1/2 and ROS [602]	High-fat diet in rats and mice, generally upregulates chemerin [604] High glucose upregulates chemerin in mice and humans [605] Chemerin: HDL ratio can be a marker for metabolic syndrome [606]
Galanin	Skeletal and heart muscle, adipose tissue and pancreas [607]	Brain, Pancreas, adipose tissue, skeletal muscles	• Show paradoxical action where initial stimulation of galanin improves insulin sensitivity but constant high circulating levels induce resistance [607]<br>• Regulates insulin resistance, hypertension and metabolism [608]<br>• High levels impair glucose tolerance [607]	Increased in metabolic syndrome [599]

			• Increases GLUT-4 expression and reduces insulin secretion from pancreatic islet cells [608]<br>• Regulates appetite via CNS interactions [608]	
IL-6	Released by inflamed adipose tissue (macrophages) [609, 610]	Hepatocytes, neutrophils, monocytes, macrophages, granulocytes, Kupffer cells, eosinophils, T regulatory cells (Treg), memory $CD4^+$ T cells, naïve T cells, dendritic cells, basophils, naïve $CD8^+$ cells, and memory $CD8^+$ cells	• In acute inflammation, has a protective role in resolution by stimulating acute phase proteins [610]<br>• Chronic inflammation (unclear role) [610]<br>• Glucose metabolism (good/bad) – maintains glycemic control in response to high-fat diet (but chronic high-fat diet?) (debatable) [610]<br>• Improve glycemic control in macrophages and hepatocytes of lean and obese rodents [610]	IL-6 increase predisposes individuals to diabetes and metabolic syndrome [609] Increased in metabolic syndrome and obesity [611]
Meteorin like protein (METRNL) (Metrnl, Meteorin-β, Subfatin, and Cometin)	Adipose tissue (BAT), skin, and mucosal barrier tissue [612]	BAT, endothelial cells	• Improves insulin sensitivity [612]<br>• Promotes BAT thermogenesis [612]<br>• Promotes adipose tissue browning [612]	It is a circulating biomarker of BAT activity [614] that negatively corelates with BMI, insulin

			• Ameliorates LPS-induced endothelial cell inflammation via AMPK and PPAR-γ [613]<br>• Stimulate an increase in eosinophils in adipose tissue and M2 macrophage polarization [613, 614]	levels, HOMA-IR [615]
FGF-21	Liver (mainly), adipose tissue (both BAT & WAT), heart, skeletal muscle and the kidney (fasting inducible messenger) [616]	Adipose tissue, liver and muscle [616]	• Upregulates fatty acid β oxidation, ketogenesis and gluconeogenesis (in fasting conditions in rodents) in adipocytes, liver and muscles [616-619]<br>• Induce adiponectin production [616, 619]	Supplementation reduces fat-mass in high-fat diet fed rodents [616] Elevated in children with metabolic syndrome [620] and in ischemic heart diseases associated with obesity [621] may enhances inflammation via NLRP3 inflammasome [622] .
Asprosin	WAT during fasting [623]	Liver, Skeletal muscle, Pancreas	• Regulate (increases) appetite (hypothalamic AgRP neurons) (controversial still) [623]	Reduced in metabolic syndrome [615], Reduction of ASP protects against hyperinsulinemia

			• Plays a role in glucose metabolism and insulin resistance by regulating beta-cell function and survival [623-626]<br>• Overexpression of asprosin in scWAT reduced browning and asprosin decreased during the browning process of white adipose tissue [627]	stimulated by exercise [627-629]

Table 2: Adipose tissue messengers with metabolic functionality interlinking different organ-functions and dysregulations in obesity/HCHF induced metabolic syndrome

MS REVIEW PAPER Revision 2

ORIGINALITY REPORT

8%	5%	6%	1%
SIMILARITY INDEX	INTERNET SOURCES	PUBLICATIONS	STUDENT PAPERS

PRIMARY SOURCES

1	www.mdpi.com<br>Internet Source	1%
2	www.frontiersin.org<br>Internet Source	<1%
3	www.ncbi.nlm.nih.gov<br>Internet Source	<1%
4	www.nature.com<br>Internet Source	<1%
5	Swasthika Gurjar, Ramanarayana Bhat A, Raghavendra Upadhya, Revathi P. Shenoy. "Extracellular vesicle-mediated approaches for the diagnosis and therapy of MASLD: current advances and future prospective", Lipids in Health and Disease, 2025<br>Publication	<1%
6	worldwidescience.org<br>Internet Source	<1%
7	pmc.ncbi.nlm.nih.gov<br>Internet Source	<1%
8	W. V. Trim, L. Lynch. "Immune and non-immune functions of adipose tissue leukocytes", Nature Reviews Immunology, 2021<br>Publication	<1%

9	core-docs.s3.amazonaws.com<br>Internet Source	<1 %
10	George A. Bray, Claude Bouchard. "Handbook of Obesity - 2 Volume Set", CRC Press, 2019<br>Publication	<1 %
11	www.science.gov<br>Internet Source	<1 %
12	eprints.nottingham.ac.uk<br>Internet Source	<1 %
13	"2015 ACR/ARHP Annual Meeting Abstract Supplement", Arthritis & Rheumatology, 2015.<br>Publication	<1 %
14	www.science.org<br>Internet Source	<1 %
15	lpmarid.wordpress.com<br>Internet Source	<1 %
16	"Abstracts", Diabetologia, 2005<br>Publication	<1 %
17	Oumaima Anachad, Amine Taouil, Wafaa Taha, Faiza Bennis, Fatima Chegdani. "The Implication of Short-Chain Fatty Acids in Obesity and Diabetes", Microbiology Insights, 2023<br>Publication	<1 %
18	"42nd EASD Annual Meeting of the European Association for the Study of Diabetes", Diabetologia, 2006<br>Publication	<1 %
19	Richard N. Re. "Molecular Mechanisms in Hypertension", CRC Press, 2019<br>Publication	<1 %

20	era.ed.ac.uk<br>Internet Source	<1%
21	Raj K. Keservani, Alka Lohani. "Nutraceuticals in Obesity Management and Control", Apple Academic Press, 2025<br>Publication	<1%
22	Hardeep Singh Tuli, Ginpreet Kaur, Satwinderjeet Kaur. "Gut Microbiota - Characterization and Food Conversions to Therapeutic Implications", Jenny Stanford Publishing, 2025<br>Publication	<1%
23	Submitted to King's College<br>Student Paper	<1%
24	Wang, Jingxuan. "The Effect of Deoxynivalenol on Intestinal Homeostasis Studied by New Approach Methodologies.", Wageningen University and Research<br>Publication	<1%
25	www.theijoem.com<br>Internet Source	<1%
26	Geissler, Catherine, Powers, Hilary. "Human Nutrition", Human Nutrition, 2023<br>Publication	<1%
27	jcdr.net<br>Internet Source	<1%
28	Isaias Dichi, Andrea Name Colado Simao. "Nutritional Intervention in Metabolic Syndrome", CRC Press, 2019<br>Publication	<1%
29	pubmed.ncbi.nlm.nih.gov	

Internet Source

<1%

30	www.jove.com<br>Internet Source	<1%
31	Jin Young Huh, Yoon Jeong Park, Mira Ham, Jae Bum Kim. "Crosstalk between Adipocytes and Immune Cells in Adipose Tissue Inflammation and Metabolic Dysregulation in Obesity", Molecules and Cells, 2014<br>Publication	<1%
32	link.springer.com<br>Internet Source	<1%
33	Almudena Gómez-Hernández, Natalia de las Heras, Beatriz G. Gálvez, Tamara Fernández-Marcelo et al. "New Mediators in the Crosstalk between Different Adipose Tissues", International Journal of Molecular Sciences, 2024<br>Publication	<1%
34	Tomislav Bulum. "Nephroprotective Properties of the Glucose-Dependent Insulinotropic Polypeptide (GIP) and Glucagon-like Peptide-1 (GLP-1) Receptor Agonists", Biomedicines, 2022<br>Publication	<1%
35	www.hindawi.com<br>Internet Source	<1%
36	Heinrich, Victoria Ann. "Developing the Microbiome-Metabolome as a Targeted Therapy for Obesity-Associated Asthma", University of Pittsburgh<br>Publication	<1%

37 Khawaja Husnain Haider. "Handbook of Regenerative Medicine - Stem Cell-Based Approach", CRC Press, 2025
Publication
<1%

38 Yanli Miao, Haojie Qin, Yi Zhong, Kai Huang, Caijun Rao. "Novel adipokine asprosin modulates browning and adipogenesis in white adipose tissue", Journal of Endocrinology, 2021
Publication
<1%

39 spotidoc.com
Internet Source
<1%

40 www.biosino.org
Internet Source
<1%

41 hdl.handle.net
Internet Source
<1%

42 www.akinik.com
Internet Source
<1%

43 www.researchgate.net
Internet Source
<1%

44 Kristyna Brejchova, Laurence Balas, Veronika Paluchova, Marie Brezinova, Thierry Durand, Ondrej Kuda. "Understanding FAHFAs: From structure to metabolic regulation", Progress in Lipid Research, 2020
Publication
<1%

45 Mary T. Keogan, Eleanor M. Wallace, Paula O’Leary. "Concise Clinical Immunology for Healthcare Professionals", Routledge, 2006
Publication
<1%

46 Patrick Schrauwen. "High-fat diet, muscular lipotoxicity and insulin resistance", Proceedings of the Nutrition Society, 2007 <1%
Publication

47 Yao Lu, Lining Chen, Yingying Lin, Yafei Zhang, Yuqi Wang, Weiru Yu, Fazheng Ren, Huiyuan Guo. "Fatty acid metabolism: The crossroads in intestinal homeostasis and tumor", Metabolism, 2025 <1%
Publication

48 Yongfei Wang, Zikai Dong, Ziyi An, Weilin Jin. "Cancer cachexia: Focus on cachexia factors and inter-organ communication", Chinese Medical Journal, 2023 <1%
Publication

49 Zadra, Giorgia, Cornelia Photopoulos, and Massimo Loda. "The fat side of prostate cancer", Biochimica et Biophysica Acta (BBA) - Molecular and Cell Biology of Lipids, 2013. <1%
Publication

50 www.trademarkelite.com <1%
Internet Source

51 "Posters (Abstracts 301-2389)", Hepatology, 2018 <1%
Publication

52 A.M. Schreiber. "General and Comparative Endocrinology - An Integrative Approach", CRC Press, 2023 <1%
Publication

53 Kevin K. W. Wang, Zhiqun Zhang, Firas H. Kobeissy. "Biomarkers of Brain Injury and Neurological Disorders", CRC Press, 2019 <1%

Publication

54 Submitted to Queen's University of Belfast — <1%
Student Paper

55 Victor R. Preedy. "Protein Intake in Health and Disease", CRC Press, 2025 — <1%
Publication

56 Yang, Zuqin. "Deciphering the Role of NFATc1 in Allergic Asthma: A Molecular and Immunological Perspective Focusing on CD4 and CD11c Cells", Friedrich-Alexander-Universitaet Erlangen-Nuernberg (Germany) — <1%
Publication

57 theses.ncl.ac.uk — <1%
Internet Source

58 Helmut Sies. "Oxidative Stress and Inflammatory Mechanisms in Obesity, Diabetes, and the Metabolic Syndrome", CRC Press, 2019 — <1%
Publication

59 J-P Montani. "Ectopic fat stores: housekeepers that can overspill into weapons of lean body mass destruction", International Journal of Obesity, 12/2004 — <1%
Publication

60 Lackey, Denise E., and Jerrold M. Olefsky. "Regulation of metabolism by the innate immune system", Nature Reviews Endocrinology, 2015. — <1%
Publication

61 Marcela Sene-Fiorese, Fernanda O. Duarte, Flavia R.R. Scarmagnani, Nadia C. Cheik et al. "Efficiency of Intermittent Exercise on — <1%

Adiposity and Fatty Liver in Rats Fed With High-fat Diet", Obesity, 2008
Publication

62	Sapna Sharma, Anand Prakash Singh. "Traditional Foods - Impact on Gut Health", CRC Press, 2025<br>Publication	<1 %
63	Wenlu Yu, Chunxiu Li, Deju Zhang, Zhangwang Li et al. "Advances in T Cells Based on Inflammation in Metabolic Diseases", Cells, 2022<br>Publication	<1 %
64	atrium.lib.uoguelph.ca<br>Internet Source	<1 %
65	journalce.powerpak.com<br>Internet Source	<1 %
66	journals.lww.com<br>Internet Source	<1 %
67	pdffox.com<br>Internet Source	<1 %
68	repositorioinstitucional.ceu.es<br>Internet Source	<1 %
69	ttu-ir.tdl.org<br>Internet Source	<1 %
70	Submitted to Galen College<br>Student Paper	<1 %
71	academicstrive.com<br>Internet Source	<1 %
72	australiaweightloss.com<br>Internet Source	<1 %

73	findresearcher.sdu.dk<br>Internet Source	<1%
74	Sang Mun Han, Eun Seo Park, Jeu Park, Hahn Nahmgoong et al. "Unique adipose tissue invariant natural killer T cell subpopulations control adipocyte turnover in mice", Nature Communications, 2023<br>Publication	<1%
75	Tanriver, Y., and A. Diefenbach. "Transcription factors controlling development and function of innate lymphoid cells", International Immunology, 2014.<br>Publication	<1%
76	benthamscience.com<br>Internet Source	<1%
77	dadospdf.com<br>Internet Source	<1%
78	dokumen.pub<br>Internet Source	<1%
79	escholarship.org<br>Internet Source	<1%
80	journals.biologists.com<br>Internet Source	<1%
81	smw.ch<br>Internet Source	<1%
82	www.abacademies.org<br>Internet Source	<1%
83	www.annualreviews.org<br>Internet Source	<1%

84	G. Gordon Gibson, Brian G. Lake. "Peroxisomes - Biology and Importance in Toxicology and Medicine", CRC Press, 2019<br>Publication	<1%
85	Tonelli Enrico, Valerio. "Beyond the Back in Chronic Low Back Pain: Investigating Gut Microbiome and Plasma Cytokine Associations With Pain and Disability.", University of Pittsburgh<br>Publication	<1%
86	jamanetwork.com<br>Internet Source	<1%
87	www.endocrine-abstracts.org<br>Internet Source	<1%
88	www.scielo.br<br>Internet Source	<1%
89	Caroline Padro Dietz, Amber Luong. "Innate Lymphoid Cells: The Innate Counterpart to T Helper Cells", S. Karger AG, 2016<br>Publication	<1%
90	D'Aquila, Theresa, Yu-Han Hung, Alicia Carreiro, and Kimberly K. Buhman. "Recent discoveries on absorption of dietary fat: Presence, synthesis, and metabolism of cytoplasmic lipid droplets within enterocytes", Biochimica et Biophysica Acta (BBA) - Molecular and Cell Biology of Lipids, 2016.<br>Publication	<1%
91	Janis Baines, Madeleine Ball, Danielle Gallegos, Jonathan M. Hodgson et al. "Food & Nutrition - Food and Health Systems in Australia and New Zealand", Routledge, 2020	<1%

Publication

92	Mohammed Gulzar Ahmed, Sumel Ashique, Arshad Farid, Gokhan Zengin. "Nanotechnology in Wound Healing - Innovations, Applications, and Future Perspectives", CRC Press, 2025<br>Publication	<1%
93	Yeganeh Mehrani, Solmaz Morovati, Fatemeh Keivan, Soroush Sarmadi et al. "Dendritic Cell-Based Cancer Vaccines: The Impact of Modulating Innate Lymphoid Cells on Anti-Tumor Efficacy", Cells, 2025<br>Publication	<1%
94	Zhuoqi Li, Ziyu Gao, Tao Sun, Shipeng Zhang, Shengnan Yang, Meilin Zheng, Hui Shen. "Meteorin-like/Metrnl, a novel secreted protein implicated in inflammation, immunology, and metabolism: A comprehensive review of preclinical and clinical studies", Frontiers in Immunology, 2023<br>Publication	<1%
95	bsdwebstorage.blob.core.windows.net<br>Internet Source	<1%
96	cardiovascres.oxfordjournals.org<br>Internet Source	<1%
97	ovarianresearch.biomedcentral.com<br>Internet Source	<1%
98	pubblicazioni.unicam.it<br>Internet Source	<1%
99	www.biorxiv.org<br>Internet Source	<1%

100	www.coursehero.com<br>Internet Source	<1 %
101	A. C. Heijboer. "Gut?Brain Axis: Regulation of Glucose Metabolism", Journal of Neuroendocrinology, 12/2006<br>Publication	<1 %
102	Alvin Berger, Matthew A. Roberts. "Unraveling Lipid Metabolism with Microarrays", CRC Press, 2004<br>Publication	<1 %
103	Bosma, M., L.M. Sparks, G.J. Hooiveld, J.A. Jorgensen, S.M. Houten, P. Schrauwen, S. Kersten, and M.K.C. Hesselink. "Overexpression of PLIN5 in skeletal muscle promotes oxidative gene expression and intramyocellular lipid content without compromising insulin sensitivity", Biochimica et Biophysica Acta (BBA) - Molecular and Cell Biology of Lipids, 2013.<br>Publication	<1 %
104	Casimir C. Akoh, David B. Min. "Food Lipids - Chemistry, Nutrition, and Biotechnology, Third Edition", CRC Press, 2019<br>Publication	<1 %
105	Dan Tan, Meric Erikci Ertunc, Srihari Konduri, Justin Zhang et al. "Discovery of FAHFA-Containing Triacylglycerols and Their Metabolic Regulation", Journal of the American Chemical Society, 2019<br>Publication	<1 %
106	Fay Paxton. "Foundations of Naturopathic Nutrition - A comprehensive guide to	<1 %

essential nutrients and nutritional bioactives", Routledge, 2020
Publication

107	Inge Kortekaas Krohn, Suzanne M. Bal, Korneliusz Golebski. "The role of innate lymphoid cells in airway inflammation", Current Opinion in Pulmonary Medicine, 2018<br>Publication	<1 %
108	Jeff L. Staudinger, Kristin Lichti. "Cell Signaling and Nuclear Receptors: New Opportunities for Molecular Pharmaceuticals in Liver Disease", Molecular Pharmaceutics, 2007<br>Publication	<1 %
109	Jie Mei, Maria Sörhede-Winzell, Charlotte Erlanson-Albertsson. "Plasma Enterostatin: Identification and Release in Rats in Response to a Meal", Obesity Research, 2002<br>Publication	<1 %
110	Jing Wu, Hang Cheng, Tete Li, Helei Wang et al. "Correlation Between Immune Lymphoid Cells and Plasmacytoid Dendritic Cells in Human Colon Cancer", Research Square, 2020<br>Publication	<1 %
111	Karine Clement, Thorkild I. A. Sorensen. "Obesity - Genomics and Postgenomics", CRC Press, 2019<br>Publication	<1 %
112	L. Lin. "Adipocyte Expression of PU.1 Transcription Factor Causes Insulin Resistance through Up-regulation of Inflammatory Cytokine Gene Expression and ROS Production", AJP Endocrinology and Metabolism, 03/27/2012	<1 %

Publication

113 Leslie C. Costello. "Metabolic Transformations of Malignant Cells: An Overview", General Methods and Overviews Lung Carcinoma and Prostate Carcinoma, 2008 <1 %
Publication

114 Mozaniel Santana de Oliveira, Leo M.L. Nollet. "Bioactive Compounds - Identification and Characterization of their Food and Pharmacological Potential", CRC Press, 2025 <1 %
Publication

115 Qian Li, Kirsty L. Spalding. "The regulation of adipocyte growth in white adipose tissue", Frontiers in Cell and Developmental Biology, 2022 <1 %
Publication

116 Ross S McInnes, Gregory E McCallum, Lisa E Lamberte, Willem van Schaik. "Horizontal transfer of antibiotic resistance genes in the human gut microbiome", Current Opinion in Microbiology, 2020 <1 %
Publication

117 Sunit K. Singh. "Human Respiratory Viral Infections", CRC Press, 2014 <1 %
Publication

118 Sushil Sharma. "Charnolophagy in Health and Disease - With Special Reference to Nanotheranostics", CRC Press, 2021 <1 %
Publication

119 William Insull. "Clinical Utility of Bile Acid Sequestrants in the Treatment of <1 %

Dyslipidemia: A Scientific Review :", Southern Medical Journal, 03/2006
Publication

120	Yaser Mohammed Al-Worafi. "Handbook of Complementary, Alternative, and Integrative Medicine - Education, Practice and Research: Volume 5: Disease Focused Efficacy and Safety Profiles: Psychiatric, Infectious, Skin, Hematologic, Oncologic, Urologic and Special Population Diseases/Disorders", CRC Press, 2025<br>Publication	<1 %
121	Yingjian Hou, Jing Li, Shuhuan Ying. "Tryptophan Metabolism and Gut Microbiota: A Novel Regulatory Axis Integrating the Microbiome, Immunity, and Cancer", Metabolites, 2023<br>Publication	<1 %
122	Zepp, Jarod A.. "The Regulatory Roles of Traf4 IL-17 Receptor Mediated Signaling and Disease Pathology.", Case Western Reserve University, 2020<br>Publication	<1 %
123	acervodigital.ufpr.br<br>Internet Source	<1 %
124	downloads.hindawi.com<br>Internet Source	<1 %
125	hal.sorbonne-universite.fr<br>Internet Source	<1 %
126	jspac.etb.iau.ir<br>Internet Source	<1 %

mdpi-res.com

127 Internet Source <1%

128 theses.gla.ac.uk
Internet Source <1%

129 www.cdilabs.com
Internet Source <1%

130 www.intechopen.com
Internet Source <1%

131 www2.mdpi.com
Internet Source <1%

132 "Integrated Physiology/Obesity", Diabetes, 2016
Publication <1%

133 Bryan Tungland. "Short-Chain Fatty Acid Production and Functional Aspects on Host Metabolism", Elsevier BV, 2018
Publication <1%

134 Helmut Brade, Steven M. Opal, Stefanie N. Vogel, David C. Morrison. "Endotoxin in Health and Disease", CRC Press, 2020
Publication <1%

135 Lee Armstrong, Colin E. Willoughby, Declan J. McKenna. "Targeting of AKT1 by miR-143-3p Suppresses Epithelial-to-Mesenchymal Transition in Prostate Cancer", Cells, 2023
Publication <1%

136 Papageorgiou, Katerina. "Impact of Adipose Tissue on Endothelial Cells: Effect of Metabolic Disorders.", Universitat de Barcelona (Spain)
Publication <1%

137	Peter Parham, Peter Parham. "The Immune System", Garland Science, 2019<br>Publication	<1 %
138	Pudupakkam K Vedanthan, Harold S Nelson, Shripad N Agashe, PA Mahesh, Rohit Katial. "Textbook of Allergy for the Clinician", CRC Press, 2021<br>Publication	<1 %
139	"2017 Annual Meeting of the American Society for Bone and Mineral Research, Colorado Convention Center, Denver, CO, USA - September 8-11, 2017", Journal of Bone and Mineral Research, 2017<br>Publication	<1 %
140	Alexander Tschoner, Wolfgang Sturm, Julia Engl, Susanne Kaser et al. "Retinol-binding Protein 4, Visceral Fat, and the Metabolic Syndrome: Effects of Weight Loss", Obesity, 2008<br>Publication	<1 %
141	Arnold, Megan Ann. "Maternal Immune Activation Alters Behavior of Adult Offspring: Sex-Dependent Impairment and Attenuation by Glycan", Auburn University, 2023<br>Publication	<1 %
142	Dudakov, Jarrod A., Alan M. Hanash, and Marcel R.M. van den Brink. "Interleukin-22: Immunobiology and Pathology", Annual Review of Immunology, 2015.<br>Publication	<1 %
143	Johanna K. DiStefano. "Angiopoietin-like 8 (ANGPTL8) expression is regulated by miR-	<1 %

143-3p in human hepatocytes", Gene, 2019
Publication

144	Miriam Bettinetti-Luque, Laura Trujillo-Estrada, Eduardo Garcia-Fuentes, Juana Andreo-Lopez et al. "Adipose tissue as a therapeutic target for vascular damage in Alzheimer's disease", British Journal of Pharmacology, 2023<br>Publication	<1%
145	Sachin K. Jain, Ravikant Gupta, Sudha Vengurlekar. "Nutraceuticals and Obesity - Strategies for Weight Management", Routledge, 2025<br>Publication	<1%
146	Stefano Fiorucci, Silvia Marchianò, Ginevra Urbani, Cristina Di Giorgio, Eleonora Distrutti, Angela Zampella, Michele Biagioli. "Immunology of bile acids regulated receptors", Progress in Lipid Research, 2024<br>Publication	<1%
147	Sun, Shengyi, Yewei Ji, Sander Kersten, and Ling Qi. "Mechanisms of Inflammatory Responses in Obese Adipose Tissue", Annual Review of Nutrition, 2011.<br>Publication	<1%
148	Yadav, Reddeboina Ashok. "Combined Effect of Emblica officinalis Fruit Extract with Oral Hypoglycemics in Diabetic Neuropathic Pain", Rajiv Gandhi University of Health Sciences (India), 2023<br>Publication	<1%

Exclude quotes	On	Exclude matches	Off
Exclude bibliography	On		
